# Differential gene expression during floral transition in pineapple

**DOI:** 10.1002/pld3.541

**Published:** 2023-11-14

**Authors:** Robert E. Paull, Najla Ksouri, Michael Kantar, Dessireé Zerpa‐Catanho, Nancy Jung Chen, Gail Uruu, Jingjing Yue, Shiyong Guo, Yun Zheng, Ching Man Jennifer Wai, Ray Ming

**Affiliations:** ^1^ Tropical Plant & Soil Sciences University of Hawaii at Manoa Honolulu Hawaii USA; ^2^ Laboratory of Genomics, Genetics and Breeding of Fruits and Grapevine, Experimental Aula Dei‐CSIC Zaragoza Spain; ^3^ Department of Plant Biology University of Illinois at Urbana‐Champaign Urbana Illinois USA; ^4^ Center for Genomics and Biotechnology Fujian Agriculture and Forestry University Fuzhou China; ^5^ State Key Laboratory of Primate Biomedical Research, Institute of Primate Translational Medicine Kunming University of Science and Technology Kunming Yunnan China; ^6^ Department of Horticulture Michigan State University East Lansing Michigan USA

**Keywords:** AGAMOUS, APETALA1/FRUITFULL (AP1/FUL), ethylene response transcription factors, floral transition, flower regulators, Flowering Locus T, Trans‐Cis motifs

## Abstract

Pineapple (*Ananas comosus var. comosus*) and ornamental bromeliads are commercially induced to flower by treatment with ethylene or its analogs. The apex is transformed from a vegetative to a floral meristem and shows morphological changes in 8 to 10 days, with flowers developing 8 to 10 weeks later. During eight sampling stages ranging from 6 h to 8 days after treatment, 7961 genes were found to exhibit differential expression (DE) after the application of ethylene. In the first 3 days after treatment, there was little change in ethylene synthesis or in the early stages of the ethylene response. Subsequently, three ethylene response transcription factors (ERTF) were up‐regulated and the potential gene targets were predicted to be the positive flowering regulator CONSTANS‐like 3 (CO), a WUSCHEL gene, two APETALA1/FRUITFULL (AP1/FUL) genes, an epidermal patterning gene, and a jasmonic acid synthesis gene. We confirm that pineapple has lost the flowering repressor FLOWERING LOCUS C. At the initial stages, the SUPPRESSOR OF OVEREXPRESSION OF CONSTANS 1 (SOC1) was not significantly involved in this transition. Another WUSCHEL gene and a PHD homeobox transcription factor, though not apparent direct targets of ERTF, were up‐regulated within a day of treatment, their predicted targets being the up‐regulated CO, auxin response factors, SQUAMOSA, and histone H3 genes with suppression of abscisic acid response genes. The FLOWERING LOCUS T (FT), TERMINAL FLOWER (TFL), AGAMOUS‐like APETELAR (AP2), and SEPETALA (SEP) increased rapidly within 2 to 3 days after ethylene treatment. Two FT genes were up‐regulated at the apex and not at the leaf bases after treatment, suggesting that transport did not occur. These results indicated that the ethylene response in pineapple and possibly most bromeliads act directly to promote the vegetative to flower transition via APETALA1/FRUITFULL (AP1/FUL) and its interaction with SPL, FT, TFL, SEP, and AP2. A model based on AP2/ERTF DE and predicted DE target genes was developed to give focus to future research. The identified candidate genes are potential targets for genetic manipulation to determine their molecular role in flower transition.

## 1 INTRODUCTION

Pineapple flower induction was initially observed in fields exposed to smoke. Later, it was recognized that ethylene gas, a component of smoke, was involved in forcing flowering (Bartholomew, 1977, 2014; Burg & Burg, 1966; Lin, Zhong, & Grierson, 2009). The pineapple inflorescence has upward of 200 spirally arranged flowers on a spadix/rachis that reverts to a vegetative crown on top of the fruit (Okimoto, 1948). Each flower is subtended by a bract and has three sepals and three petals in the outer two whorls, six stamens in the outer two whorls, and a central pistil with three fused carpels. This flower is similar to the ancestral monocot flower minus the two outer perianth whorls (Hu et al., 2021; Remizowa et al., 2010; Sauquet et al., 2017; Smyth, 2018). This ancestral nature would be expected to involve similar MADS‐box genes in inflorescence and flower development (Callens et al., 2018). The ABCDE model groups genes that are responsible for the development of specific floral organs (Meyerowitz, 1997; Weigel & Meyerowitz, 1994). Except for those A function genes that are APETALA2 (AP2), the others are MIKC‐type MADS‐box transcription factors (Callens et al., 2018; Schilling et al., 2020). The same groups of genes have been confirmed in grasses (rice, maize) (Chongloi et al., 2019; Wu et al., 2017; Yoshida & Nagato, 2011), pineapple (Lv, Duan, Xie, Liu, et al., 2012; Wang, Li, et al., 2020), and ornamental bromeliad ( *Aechmea fasciata* ) (Li, Wang, et al., 2016). For pineapples, recent flowering research has included changes in plant growth regulators (Liu et al., 2011) and gene expression (Li, Wu, et al., 2016; Liu & Fan, 2016; Liu, Liu, et al., 2018), although the molecular mechanism behind flower induction is still unclear.

An ethylene response pathway for flower induction has been proposed based on genetic analyses of Arabidopsis mutants sensitive to ethylene (Ma et al., 2014). The first step is the binding of ethylene to its receptors (Chang & Stadler, 2001). Five ethylene receptor genes have been identified in Arabidopsis—ETR1, ETR2, ERS1, ERS2, and EIN4 (Chen et al., 2005; Hua et al., 1998; Kendrick & Chang, 2008; O'Malley et al., 2005; Schaller & Kieber, 2002)—and four are predicted in pineapple (Li, Wu, et al., 2016). In the absence of ethylene, receptors actively suppress the ethylene response pathway; binding of receptors to ethylene removes this suppression (Wen et al., 2015). The five Arabidopsis receptors are involved in ethylene signaling with overlapping roles in response regulation (Hall & Bleecker, 2003; Hua et al., 1995, 1998), with different receptor subfamilies having unique functions (Binder et al., 2004, 2006; Hall & Bleecker, 2003; Kevany et al., 2007; Liu et al., 2010; O'Malley et al., 2005; Plett, Cvetkovska, et al., 2009; Plett, Mathur, & Regan, 2009; Qu et al., 2007; Seifert et al., 2004; Wang, Cui, et al., 2013; Wilson et al., 2014; Xie et al., 2006). In tomato, specific ethylene receptors mediate fruit ripening; other receptors had little effect (Kevany et al., 2007; Tieman et al., 2000). The four ethylene receptors in pineapple (Li, Wu, et al., 2016) suggest that pineapple induction may involve specific receptors.

In pineapple planting regions, natural flowering on short days with cool nights is a production problem (Bartholomew et al., 2003; Bartholomew & Sanewski, 2018; Friend & Lydon, 1979; Gowing, 1961). Natural flowering in Hawaii results in a harvest from May to July harvest, with increased labor due to lack of synchrony (Zhu et al., 2012). Once reproductive development begins, it cannot be stopped (Bartholomew et al., 2003). Therefore, to produce fruit in every month of the year, ethylene or ethephon (which degrades to produce ethylene) has been widely used to induce flowering in pineapples (Bartholomew et al., 2003). Although there has been much progress in understanding the use of ethylene in the field to induce flowering, there is limited understanding of the physiological and molecular pathways involved in this vegetative‐to‐flower conversion.

The gaseous plant hormone ethylene, with its related acetylene, was the first commercially used plant growth regulator (Bartholomew, 2014; Rodriguez, 1932). Ethylene is an important regulator of numerous plant growth and development functions, including flower development and fruit ripening (Abeles et al., 1992; Ma et al., 2014; Wen et al., 2015). Ethylene plays a role in the regulation of flower timing, but its effects appear to vary (Ma et al., 2014). In Arabidopsis, ethylene promotes floral transition; however, the wild type treated with ctr1 and 1‐aminocyclopropane‐1‐carboxylic acid (ACC) showed delayed flowering (Ogawara et al., 2003), suggesting that ethylene inhibits Arabidopsis flowering (Achard et al., 2007). The reverse response is observed in rice, where overexpression of OsETR2 reduces ethylene sensitivity and delays the floral transition, whereas suppression of OsETR2 by RNAi enhances ethylene sensitivity and accelerates flowering (Wuriyanghan et al., 2009). Overexpression of mutants with loss of function of OsCTR2 and osctr2 delayed flowering, and ethylene represses the floral transition in rice (Wang, Zhang, et al., 2013). Though recognized in the 1930s as inducing the vegetative‐to‐flowering transition in pineapple, the underlying molecular mechanism remained unknown.

**FIGURE 1:**
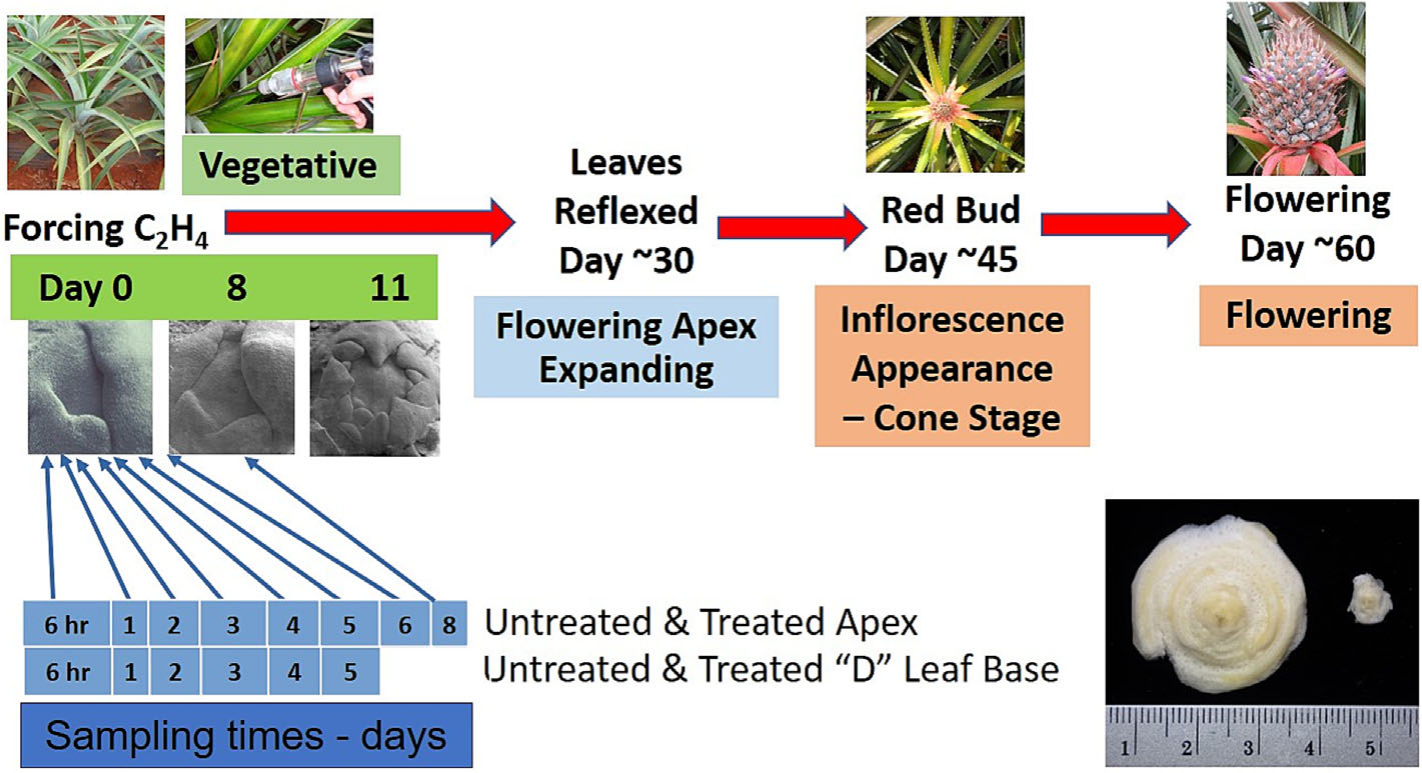
Graphical depiction of the change in flowering and when the RNA sampling occurred. At day 0 forcing induces flowering and the apex transition from vegetative to reproductive occurs in during the first week. Following the apex transition, flower and fruit development occurs normally. Lower right photographs indicated on the left the size of the apex after initial trimming and on the right the apex actually used to extract RNA. Apex micrographs of days 0, 8, and 11 after induction were kindly provided by Dr. Duane Bartholomew.

Here, we report gene expression in the first 8 days after flower induction (forcing) with ethephon during the critical phase when the apex changes from a vegetative to a floral meristem (Figure 1). In the first 3 days after ethephon treatment, no dramatic changes in ethylene synthesis, ethylene receptors, or early stages of the ethylene response pathway were found. Similarly, in these first 3 days, GA 2‐oxidase involved in GA degradation was not detected with no change in DELLA gene expression, implying that the GA pathway was not directly involved in this flowering transition, as reported in Arabidopsis (Bao et al., 2020). Changes in the expression of specific ethylene response transcription factors (ERTF) of the AP2/ERTF family occurred and the expression increased for genes associated with floral induction and later with the development of the floral meristem.

## 2 MATERIALS AND METHODS

### 2.1 Plant material

A uniform plot with pineapple plants weighing 1.5 to 2 kg was selected in a commercial field (Cultivar “MD1,” PRI 73‐50) at Dole Plantation, Wahiawa, Hawaii (21° 31′ 52.6 N; −158° 03′ 35.3 W) 6 weeks before commercial forcing. The plot was divided into three replications with two treatments (control and treated) and ~50 plants in each treatment block. Water (10 ml) as control treatment or ethephon (50 mg ai in 10 ml) (Ethrel, Rhone‐Poulenc, AG Company, North Carolina) as flower induction treatment was injected into the center of the plant between 7:00 and 7:20 AM (Figure 1). From 8:30 to 8:45 AM, three plants were harvested from each replication in the water control and ethephon‐treated plants at each sampling time after treatment. Four grams of the “D” leaf base (most recently matured leaf) and the other leaves of the uprooted plants were trimmed in the field from the cut stem. The trimmed leaf bases and the trimmed stem were chilled and immediately returned to the laboratory on ice at 10 AM for further processing. The bases of the trimmed leaves were removed from the apex of the apex of the stem to expose the stem (Figure 1), and <1 g of the apex was taken from each stem and frozen in liquid nitrogen. This physical processing of the leaf bases and apex, especially the care needed to carefully remove the leaf bases from the shoot apex of the three control and treated plants of each of the three replications, was completed by 2:00 PM and reported here 6 h after ethephon or water control treatment. Subsequent leaf samples at the apex and “D” were taken on 1, 2, 3, 4, and 5 days and the apex also on 6 and 8 days after treatment (Figure 1) between 8:00 and 8:30 AM and processed by noon in the laboratory. Leaf bases were included as a control to allow comparison with non‐apex tissue, because leaf primordia remained on the excised apex. All samples were stored at −80°C until ground into powder in liquid nitrogen for RNA extraction.

### 2.2 RNA and miRNA extraction and library construction

#### 2.2.1 RNA extraction

Total RNA was extracted from the apex and leaf bases using the Qiagen RNeasy Plant Mini Kit (Qiagen, #74904) following the manufacturer's protocol. DNA was removed with the DNA‐free DNA Removal Kit (Life Technologies, #AM1906M). Three biological replicates were sequenced for each sampling stage with three apices or leaf bases in each replication.

#### 2.2.2 Sequencing

Total RNA (2 μg) was used for the preparation of the mRNA‐Seq library using the TruSeq® Stranded mRNA LT kit (Illumina, USA) according to the manufacturer's protocol. The size of the RNA‐Seq library was evaluated by electrophoresis (1 μl of sample + 1 μl of loading dye 6X, 1% Agarose, TBE 1X buffer, 30 min, 60 V), and 1 μl of sample was used for quantification with a Qubit® Fluorometer (Invitrogen, USA) using the DNA HS assay kit. The multiplexed pooled libraries were sequenced on an Illumina HiSeq4000 with a read length of 50 nt. Small RNA libraries were prepared from total RNA using the NEBNext Multiplex Small RNA Sample Kit for Illumina (E7300, NEB, Ipswich, MA) following the manufacturer's protocol. The libraries were quantified by qPCR and sequenced in each lane with a read length of 50 nt.

#### 2.2.3 Data analysis

Raw sequencing reads were quality assessed using FastQC (v0.10.1) (Bolger et al., 2014). The adapters and low‐quality reads were then removed using Trimmomatic (v036). The high‐quality reads were aligned with Hisat2 (v2..5) with the pineapple genome assembly “Acomosus_321_v3.fa.gz” downloaded from Phytozome V12 (Xu et al., 2018). SAM files (sequence alignment map format) were converted to binary format (BAM files) using SAMtools (v1.2). Reads aligned with exons were counted using featureCounts (v1.6.0) and summarized by gene ID. For counting, the annotation file “Acomosus_321_v3.gene_exons.gff3.gz” of Phytozome was transformed from gff3 to gtf using gffread (v0.9.8). This gtf file was used to count the number of aligned reads as previously described (Ming et al., 2015).

### 2.3 Differential expression analysis

Raw counts of mapped transcripts were converted to CPM values (counts per million) using the featureCounts function of the edgeR package (Robinson et al., 2010). Genes that did not have at least one CPM in more than three libraries were excluded from the analysis. This number was set to be equal to the number of replicates for each treatment. Subsequently, the counts were normalized in R using TMM normalization and log2 CPM values were obtained (Table ST1). Pairwise differential expression at each sampling was performed using edgeR (Robinson et al., 2010) from the OmicsBox bioinformatic platform (BioBam Version 2.1.10; https://www.biobam.com/omicsbox) (Table ST2). Because no counts were found in the control treatment for some genes, no filtering was applied. The pairwise comparison approach is analogous to the Fisher exact test.

### 2.4 Annotation

BLASTp NCBI database Model organisms (Landmark)‐higher plants (Taxid 3193) was used with stringent criteria of (|log2 fold change| > 2) and FDR adjusted *p*‐value of less than .05 (*Q* < .05) were considered to exclude differently expressed genes (DEG). The DEG KEGG reference pathway map and function were determined with KEGG website (https://www.genome.jp/kegg/) (Table ST2). InterPro Classification Accession number, annotation, and GO for DEGs were determined with the OmicsBox bioinformatic platform (BioBam Version 2.1.10; https://www.biobam.com/omicsbox) (Table ST2 and Figure SF1). To validate our expression data, published transcriptome data for the pineapple flower and the first two stages of fruit development were downloaded from the Pineapple Genomics Database http://pineapple.angiosperms.org/pineapple/html/index.html (Xu et al., 2018). The first stage of fruit development in that dataset was in the half‐flower stage, and the second stage was early vegetative growth. Genes when described by other authors were indicated in brackets after the pineapple genome ID.

### 2.5 Transcription factor binding motifs

#### 2.5.1 Prediction of DNA transcription factor motifs

A set of 41 transcription factors (TF), especially AP2/ERTF genes that showed temporal differential expression, were evaluated for their potential DNA binding motifs using the footprintDB database (Sebastian & Contreras‐Moreira, 2013). TFs bind to short sequences known as TF‐binding sites (TFBS). The different sites recognized by a TF were summarized as motifs. Therefore, a motif is a “*consensus*” sequence of multiple aligned binding sites. The corresponding peptide sequences of each TF were submitted to footprintDB, where a BLASTP search was performed against the 3D footprint library (http://floresta.eead.csic.es/3dfootprint/download/list_interface2dna.txt). The best candidate motifs were selected in *Arabidopsis thaliana* based on their BLAST *E value* generally below .01 and the best interface similarity.

#### 2.5.2 Positional distribution of transcription factor binding sites

To verify the positional distribution of the TFBS in the proximal promoter region of the *Ananas comosus* genes, position‐specific scoring matrices of all candidate DNA motifs were scanned along the upstream interval [−1,500 bp, +200 bp] around the TSS (transcription start site). Scanning analysis was performed using the matrix scan tool of the regulatory sequence analysis tools (RSAT:Plants, http://rsat.eead.csic.es/plants/) (Turatsinze et al., 2008). The Markov chain of order 1 (*m* = 1) was used as a background model and the *p*‐value <1E‐4 as a cutoff to retain the high‐scoring sites.

#### 2.5.3 Prediction of pineapple genes that are potentially regulated by transcription factors

In addition to the characterization of regulatory elements (motifs), the identification of genes regulated by one or more transcription factors was carried out. To do so, the TFBSs were scanned along the region −1500 bp, +200 bp of all genes in the pineapple genome using a Markov model of order 2 and a *p*‐value <1E‐6. To retain the most relevant predictions, the target genes were selected based on the positional distribution of each motif. To shortlist pineapple genes potentially regulated by AP2‐ERTF, only those having AP2‐TFBS around the region interval [−400, −200 bp] upstream of the start codon were considered.

#### 2.5.4 Phylogenetic analysis of AP2/ERTF

The pineapple genome database referenced above was searched for all members of the AP2/ERTF family. The BLASTP in NCBI was used to screen the predicted amino acid sequences. Pineapple DE AP2/ERTF genes were BLASTP against the Arabidopsis AP2/ERTF PlantTFDB database at http://planttfdb.gao-lab.org/prediction_result.php (Jin et al., 2017). Sixteen AP2 ANT groups were detected and 12 were expressed, whereas the four AP2–AP2 groups were expressed. Sixty AP2/ERTF in various groups were predicted, and of these, 47 were expressed (Table ST5). Family and group based on Nakano et al. (2006).

### 2.6 Network and temporal analysis

The gene coexpression network was inferred using GWENA (Lemoine et al., 2021). This software is a modified version of WGCNA (Langfelder & Horvath, 2008), which includes methods for visualizing coexpression networks, network modules, hub gene detection, and differential coexpression. The network was visualized in the R program (R Core Team, 2021), and the weight cutoff was established at *a* < .01. The network is a scale‐free weighted gene network with multiple nodes representing genes and connected by edges.

To detect significant temporal expression changes and significant contrasts between the treated and control “maSigPro” (Bioconductor) with GLM in OmicsBox (BioBam v2.1.10) was used (Nueda et al., 2014). The software applies a two‐step regression strategy to find genes that show significant expression changes over time and between experimental groups. The initial number of total characteristics was 27,024, and the identified DE characteristics (DEG) (False Discovery Rate < .05) was 15,895. Genes with significantly different expression levels were classified into 15 groups according to the dynamics of change (Table ST2).

### 2.7 Small RNA

Small RNA sequencing profiles were processed as previously reported (Zheng et al., 2016). Briefly, the three “adapters” of the reads were cut from the raw sequencing reads. Unique small RNAs along with count values were obtained after redundant sequences. The unique small RNAs were aligned with mature miRs in miRBase (v22) (Kozomara et al., 2018) to identify sequenced miRs and their raw count values. The raw count values were then normalized by calculating their reads per 10 million sequencing tags. The average RPTM of the miRs are summarized in Table S7. Pairwise differential expression of every gene expressed gene (27,024) at each sampling time was determined based on “edgeR” (Bioconductor) (Robinson et al., 2010) in OmicsBox (BioBam version 2.1.10). To detect significant temporal expression changes and significant differences between treated and control, “maSigPro” (Bioconductor) was used that incorporated GLM into OmicsBox (BioBam Version 2.1.10) (Nueda et al., 2014).

## 3 RESULTS

The scope and depth of the transcriptome database required that criteria be established to expedite interpretation. Criteria to categorize differentially expressed (DE) genes were ethylene synthesis and response genes, published genes on the role of ethylene in flowering in model systems, MADS‐box genes known to be involved in floral identity and flowering, auxin, GA, and JA genes associated with flowering and small RNAs. Results SR1 presents results not primarily regarded as directly involved in the meristem transition to a floral state. This analysis includes DEGs associated with cytokinin synthesis and response, abscisic acid, LATE EMBRYOGENESIS ABUNDANT (LEA) genes, nodulin‐related, stress‐related, sugar metabolism, OVATE, and light‐dependent short hypocotyl, protein turnover and interactions, regulation of transport gradients, sugar metabolism, and transporters, and our network (Figure SF5) and temporal analysis (Table ST2). All expressed genes and their orthologs (Table ST1) as Log_2_ mean and standard error for the tissue of the apex and leaf bases at all sampling times, and the DEGs determined by edgeR can be found in Table ST2.

### 3.1 Flower field induction effectiveness

Three weeks after ethephon treatment (“forcing”) and a fortnight after our last sampling was completed, the control and ethephon‐treated plants remaining in the field were evaluated. Of the 58 ethephon‐treated plants remaining (~14 plants/replication) all showed reflexing of the leaves, an early sign of flowering. At 8 weeks after ethephon treatment, the plants in the field test blocks showed the “red bud” stage of early visible flower development, whereas the untreated plants (*n* = 48; ~12 plants/replication) showed no leaf reflex and no “red bud” flowering (Figure 1). There was a significant difference between the control and treated plants (Wilcoxon‐test, *p* < .001). These results confirm the effectiveness of the ethephon treatment in inducing the shoot apex to floral transition.

### 3.2 Sequencing, assembly, and annotation

The overall aligned read percentage ranged from 69.3% to 84.2%, with an average of 76.6%. The number of raw reads was 544,154,716 with 11.3% unassigned, 9.58% unassigned without features, and 2.59% unassigned due to ambiguity. There was a range in the number of reads per sample (1,699,238 to 27,965,655) (Figure SF2). The mean and standard error for all samplings, treatment, and apex and leaf base tissue can be found in Table ST1.

### 3.3 Global analysis of differential expression profiling

At each sampling stage, differences in gene expression were measured relative to the untreated control. During the eight apex sampling stages, 7961 genes were found to exhibit differential expression after the application of ethephon. These DEGs were up‐ or down‐regulated in one or more sampling stages and in at least one tissue (apex, leaf base) (Table ST2). 1002 DEGs were classified as hypothetical proteins (12.6%), 249 genes were annotated as proteins with domains of unknown function (DUF) (3.1%), and another 150 as uncharacterized proteins (1.9%). During the 8 days of sampling, four trends were observed: (1) some genes showed a rapid increase of more than two times within 6 h of treatment, often followed by (2) a decrease. Other genes showed (3) increased after three to 4 days, whereas another group of genes (4) decreased throughout the sampling period or at some stage in the middle of the sampling period. Expression during the first 3 to 4 days was considered critical, as these genes played a central role in ethylene response from the application to the initiation of genes that were potentially involved in the conversion of the apex from the vegetative to the floral state.

**FIGURE 2:**
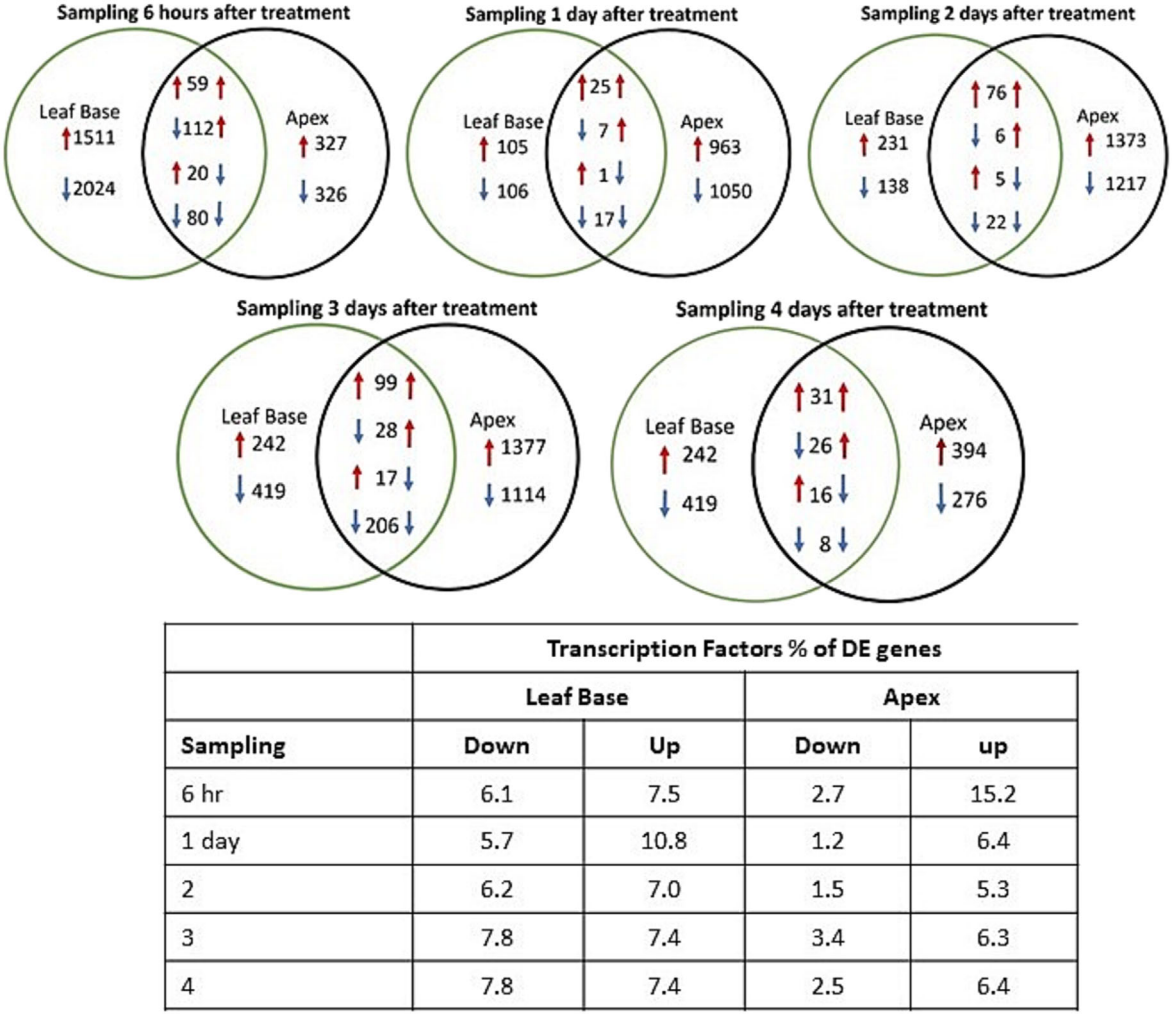
Overlap in genes differentially expressed (DE) up and down between the apex and leaf base tissues at 6 h and 1, 2, 3, and 4 days after flower induction with ethephon. The table gives the percentage of DE genes that were transcription factors. Data were derived from Table S2.

Genes up‐regulated ≥2‐fold in the apex or base tissue at 6 h and 1, 2, 3, and 4 days after flower induction showed minimal overlap (Figure 2). Six hours after treatment, the number of DEG was six times higher in the leaf base than in the apex with a two‐fold higher percentage of transcription factors (7.5 vs. 15.2%). The 271 DEGs that were expressed differentially in both tissues contained 59 up‐regulated genes and 80 down‐regulated genes in both tissues, 112 down‐regulated genes down‐regulated in the leaf base and up‐regulated in the apex, and only 20 genes showed the reverse response. In subsequent samplings, DEGs were five to 10 times more prevalent in the apices than in the leaf bases, and the overlapping DEGs differed (Table ST2). Furthermore, this change in the expression pattern occurred on day 1, suggesting that once the change from vegetative to floral apex begins, an increasing number were DE in the apex. A higher percentage (two‐ to four‐fold) of genes expressed at the treated apex were transcription factors on days 1 through 4. On day 1, only 25 genes were up‐regulated and 17 down‐regulated at both the apex and leaf bases, with the only gene appearing on days 1, 3, and 4 being MADS‐box transcription factors down‐regulated in both tissues (Aco014671) (Table ST2). At the leaf bases, the percentage transcription factors of DEG were similar except on day 1 when there was a two‐fold increase in DEG up‐regulated. In addition to transcription factors, genes expressed on day 1 in apex and leaf base tissues included a calcium exchanger (Aco004292), a sucrose phosphate synthase (Aco017378), a 2‐oxoglutarate (2OG) and Fe (II)‐dependent oxygenase superfamily protein (Aco003280), and one unknown (Aco010021) occurred in the overlap. In both tissues, a SAUR‐like auxin‐responsive protein (Aco026644) was expressed on day 1, and a AP2/ERTF cytokinin response factor 2 (Aco010738) was expressed on day 2 (Table 1). Among DEGs in the treated and untreated apex or in the treated and untreated leaf base, no genes were DE at all stages and similar numbers occurred at each stage except for leaf bases on day 3.

**TABLE 1:** Differential expression (edgeR *p* ≤ .05) of AP2/ERTH ethylene‐responsive transcription factors of the apex and leaf bases after ethephon treatment, and their Arabidopsis phylogenetic group (Nakano et al., 2006).

Gene	Arabidopsis Group	Apex 0.25	Leaf 0.25	Apex 1 day	Leaf 1 day	Apex 2 day	Leaf 2 day	Apex 3 day	Leaf 3 day	Apex 4 day	Leaf 4 day	Apex 5 day	Apex 6 day	Apex 8 day
Aco000576	VI	Up												Down
Aco001190	Ib	Up	Down	Up		Up		Up		Up		Up	Up	
Aco001318	Xb	Up		Up		Up	Up		Down	Down	Down			Down
Aco001600	IVa								Down		Down			Down
Aco001844	VIIa	Up	Up	Up				Up				Up	Up	
Aco002673	IIb							Up						
Aco002699	VIIa			Up	Down				Down		Down	Up		
Aco002824	Ib	Up		Up		Up		Up				Up		Down
Aco003196	Xa		Down					Down	Down	Up	Down			Down
Aco004208	VIIIa			Up		Up		Up						Down
Aco005324	IXa		Down											
Aco005509	Ib		Down	Up		Up	Up		Down		Down			Down
Aco006004	IIIe			Up				Down						
Aco006567	VIIIa			Up		Up		Up	Down	Up	Down	Up	Up	
Aco006883	IXc			Up		Up								
Aco008323	VIIIa			Up		Up				Up		Up		Down
Aco009511	VIIa					Down		Down						
Aco010430	IXc							Down	Down		Down			
Aco010545	VIIIa			Up		Up		Up				Up		Down
Aco010738	VI			Up		Up		Up						
Aco011669	IXa			Up				Up				Up		
Aco012157	VIIa		Up			Down		Up						
Aco012562	VI		Down	Up		Up								
Aco012835	IIIe		Down					Down						Down
Aco012858	IXb			Up				Up				Up		
Aco012860	IXa													Down
Aco016346	Ia	Up	Down	Up	Up	Up				Up				Down
Aco017803	Xa		Down					Down						
Aco018023	IIc		Down						Down		Down			
Aco018224	Xc		Down					Down		Up				Down
Aco018980	IIb	Up	Up											Down
Aco021063	VIIa							Down						
Aco022517	IIIc								Down		Down			

*Note*: Aco000347, Aco006074, and Aco010738 best hit were as cytokinin response factor 2, and Aco000576 and Aco012562 were cytokinin response factors 4.

### 3.4 Differentially expressed genes (DE)

#### 3.4.1 Ethylene‐related genes

**FIGURE 3:**
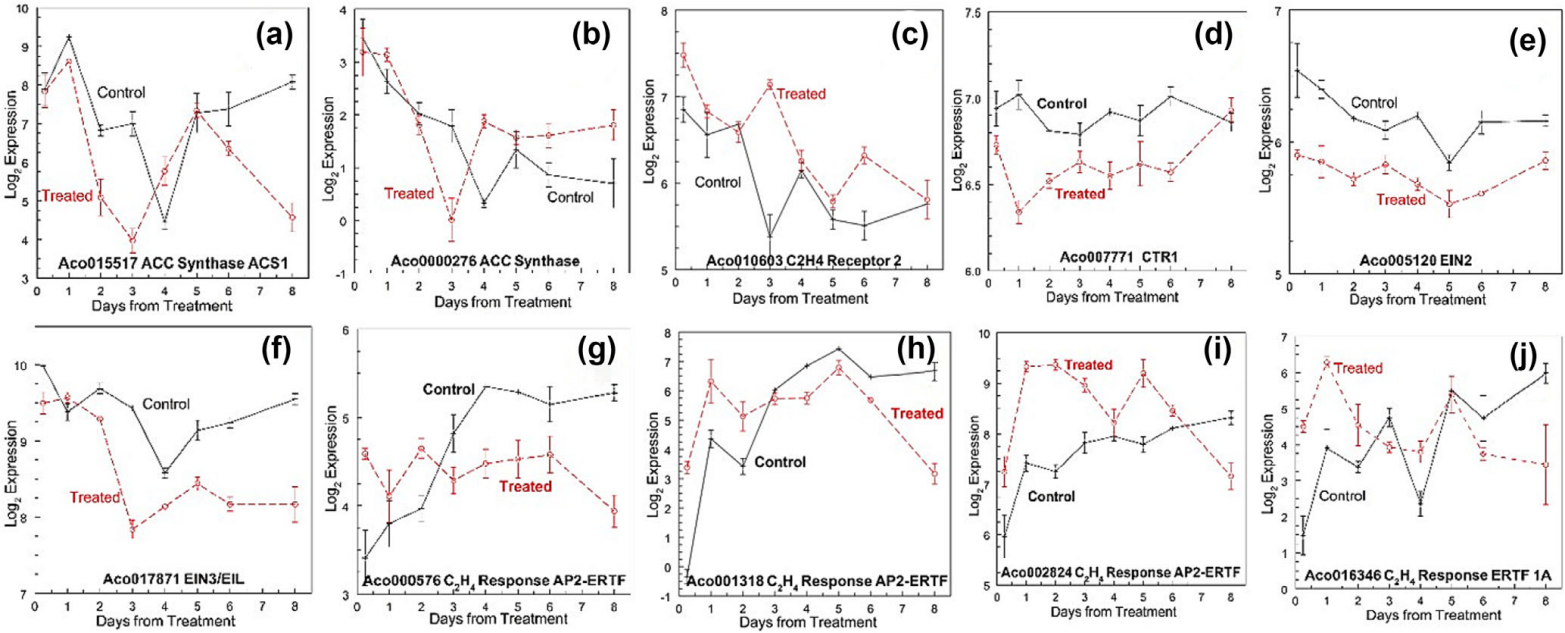
Gene expression related to ethylene response genes. (a) Aco015517 Acc synthase (ACS1), (b) Aco0000276, ACC synthase, (c) Aco010603 Ethylene receptor, (d) Aco007771 CTR1, (e) Aco005120 EIN2, (f) Aco017871 EIN3/EIL, (g) Aco0000576, Ethylene response transcription factor AP2‐EREBP, (h) Aco001318 Ethylene response transcription factor AP2‐EREBP, (i) Aco002824 Ethylene response transcription factor AP2‐EREBP, and (j) Ethylene response transcription factor 1A. Log_2_ means + standard error, *n* = 3.

Three 1‐aminocyclopropane‐1‐carboxylate synthase (ACS) genes were expressed: Aco015517 (AcACS1) (Figure 3a), Aco000276 (AcACS2) (Figure 3b), and Aco028694. The ACS2 gene (Aco000276) and the third ACS gene (Aco028694) were not DE at the leaf base or apex during sampling. The ACS gene described as ACS1 (Aco015517) (Figure 3a) was negatively regulated at 6 h and on days 3 and 4 at the leaf base and negatively regulated at the apex on days 2 and 3 and again on days 6 and 8.

Four 1‐aminocyclopropane‐1‐carboxylate oxidase (ACO) genes showed differential expression (Aco001358, Aco003285, Aco005735, and Aco015240). Aco001358 at the apex was down‐regulated on day 3 and up‐regulated from days 4 through 8, and down‐regulated in the leaf base at 6 h (Table ST2). At 6 h, Aco005765 was up‐regulated, and Aco03285 was down‐regulated only at the leaf base. Aco015420 was up‐regulated at the apex at 6 h and down‐regulated on day 2, and in the leaf, it was up‐regulated at 6 h and 1 day after treatment.

Five pineapple ethylene receptors (Aco002499, Aco006353, Aco010452, Aco010603, and Aco24515) were expressed (Table ST1), but only Aco010603 was DE (Figure 3c), up‐regulated at 6 h and down‐regulated at day 2 in the leaf bases, and up‐regulated in the apex on day 3.

The predicted ethylene response pathway protein kinase gene CTR1 (Aco007771) (Figure 3d) and a possible EIN2 (Aco005120) (Figure 3e) were not DE in either tissue. Three EIN3/EIL genes (Aco001697, Aco015335, and Aco017871) were DE. Aco001697 was up‐regulated only at the apex on day 3. Aco015335 was up‐regulated at 6 h and down‐regulated at 2 days at the leaf bases. Aco017871 was down‐regulated on days 3, 6, and 8 at the apex (Figure 3f).

Eighty‐eight AP2/ERTF were predicted for pineapple, of which 63 were expressed during the apex transition. Phylogenetic analysis grouped 12 of the genes expressed as AP2 ANT and four as AP2/AP2 (Table ST5). Forty‐seven were ethylene‐responsive transcription factors (ERTFs) that were expressed at the apex and/or leaf base, of which 33 ERTFs were DE (Table 1). Of particular interest were the seven ERTFs up‐regulated in the axis within 6 h after treatment (Aco000576, Aco001190, Aco001318, Aco001844, Aco002824, Aco018980, and Aco016346), some of which were also up‐regulated in the leaf base. These seven ERTFs were phylogenetically grouped into Arabidopsis/Rice clusters VI, Ib, Xb, VIIIa, Ib, IIb, and 1a, respectively (Table 1). During the 8 days of sampling, Aco000576, Aco001318, and Aco002824 (Group VI, Xb, and Ib, respectively) were expressed and up‐regulated only in the apex (Figure 3g,h,i). Aco000347 (Group VI) was expressed at very low levels but was not DE at the treated apex, although the expression tended to be greater than in the control. The homologs Aco010738 and Aco000347, both grouped in group VI in Arabidopsis, were described as cytokinin response factors 2. ERTF (Aco016346, Group 1a) was also rapidly up‐regulated within 6 h (Figure 3j). Most fold changes in DEG were generally higher in the first 4 days, with apex ERTFs up‐regulated and leaf base ERTFs often down‐regulated. ERTF Factor 13 (Aco022651) tended to be higher at the treated apex from day 5 onwards.

#### 3.4.2 Auxin transport and response

The three auxin influx transporters (Aco004405 [AcABCg33], Aco030876, and Aco031846 [AcABCG32]) were first up‐regulated in the leaf base at 6 h and then at the apex at day 1, remaining elevated at the apex for most of the samplings (Table ST2). The seven efflux carriers showed very different patterns of differential expression. Aco000734, which in the leaf base was up‐regulated at 6 h and then down‐regulated at day 1, was down‐regulated at the apex on day 3. Aco005423 was only up‐regulated at the apex on day 2, and Aco009213 was down‐regulated at the leaf base on 6 h and the apex on day 1 and then up‐regulated at the apex on days 4 and 6. Aco007145 was up‐regulated in the leaf base on day 2 and in the apex on day 3. Aco011167 was down‐regulated at the apex on day 1 and day 2. Aco011298 was up‐regulated at the apex on days 3, 6, and 8, and Aco24698 was up‐regulated in the leaf base on day 2 and in the apex on day 3.

Of the two auxin response factors, Aco009671 was up‐regulated on days 2 and 3 at the apex alone, and Aco009779 was up‐regulated at the leaf base at 6 h and at the apex on days 3 and 4 (Table ST2). The auxin‐associated dormancy factor Aco002473 decreased and tended to be lower in the treated than in the control throughout the sampling period.

#### 3.4.3 Synthesis, perception, and response

**FIGURE 4:**
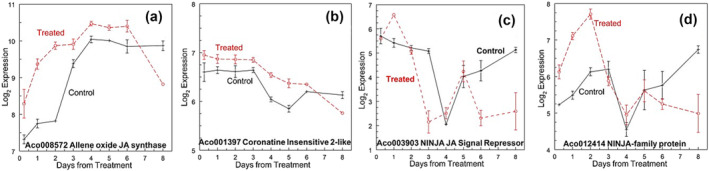
Jasmonic acid synthesis, perception, and response. (a) Aco008572 Allene oxide JA synthetase, (b) Aco001397 Coronatine‐insensitive 2‐like, (c) Aco003903 NINJA‐family protein, and (d) Aco012414 NINJA‐family protein. Log_2_ means ± standard error, *n* = 3.

The jasmonic acid (JA) synthesis gene, allene oxide synthase (Aco008572) (Figure 4a), increased rapidly at the apex only, immediately after treatment. The potential receptor, the coronatine‐insensitive 2‐like receptor (Aco001397), did not show differences in expression between the treated and untreated apex (Figure 4b). A NINJA gene that suppresses JA signaling (Aco003903) (Figure 4c) was up‐regulated on day 1 and then down‐regulated on day 3 at the apex and leaf base. Another NINJA protein (Aco012414) was differentially up‐regulated on days 1 and 2 only at the apex (Figure 4d). Five TIFY genes were DR up‐regulated 6 h after treatment (Figure SF1). A TIFY gene (Aco009689), named for a highly conserved zinc finger domain, was up‐regulated in the leaf base on days 1, 3, and 4 and in the apex on days 1 through 3 (Table S2).

#### 3.4.4 DELLA‐GA genes after induction

**FIGURE 5:**
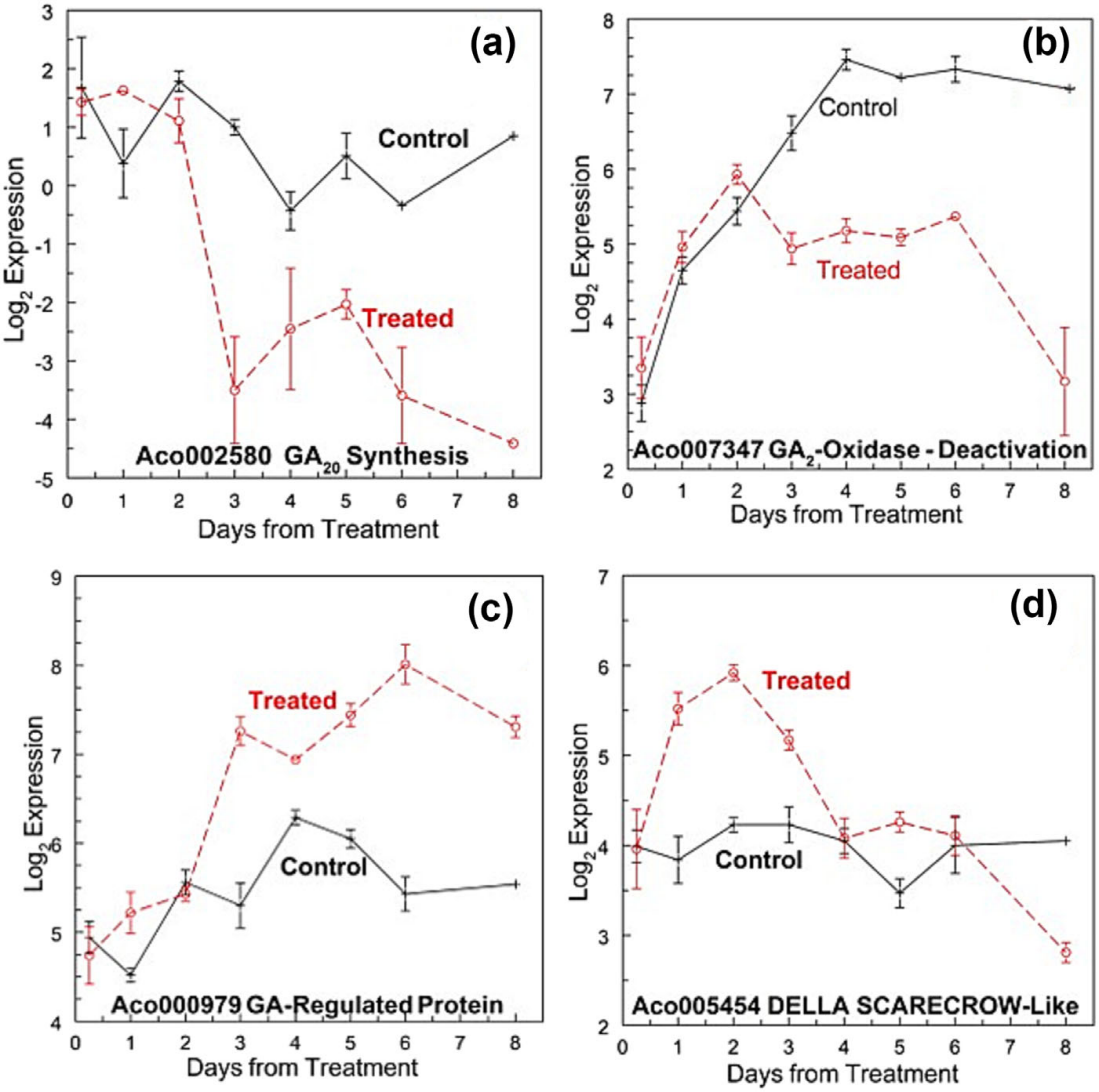
GA synthesis, degradation receptor, and DELLA genes. (a) Aco002580 GA20 synthesis, (b) Aco007347 GA‐2 oxidase deactivation, (c) Aco000979 GA‐regulated protein, and (d) Aco005454 DELLA Scarecrow‐like 21. Log_2_ means ± standard error, *n* = 3.

The breakdown of gibberellin and DELLA has been implicated in the flowering of Arabidopsis flowering, although our data did not support this conclusion for pineapple. Two genes involved in GA synthesis (GA20‐oxidase) at the apex and the leaf base (Aco002580 and Aco009790) were not DE (Figure 5). GA20ox (Aco002580) is also involved in synthesis, although it tended to decline and was not DE (Figure 5a). GA2‐oxidase (Aco007347) involved in GA degradation was negatively regulated at the apex on day 3 (Figure 5b), although a GA‐regulated protein (Aco000979 [Figure 5c] and Aco025093) was up‐regulated at the apex only on day 3 and on days 5 through 8 (Figure 5). The expression of the GA receptor (GID1, Aco003526) was low and was not DE at the apex or leaf base (Table ST2). Expression was observed in three DELLA genes; Aco003635 and Aco005453 were up‐regulated at the apex at 6 h only, and Aco025081 was up‐regulated in the apex only on day 1 and down‐regulated on day 3. DELLA‐SCARECROW‐like 21 (Aco005454) was up‐regulated on days 1 through 3 (Figure 5d).

#### 3.4.5 Perception and transduction of extracellular signal kinases

**FIGURE 6:**
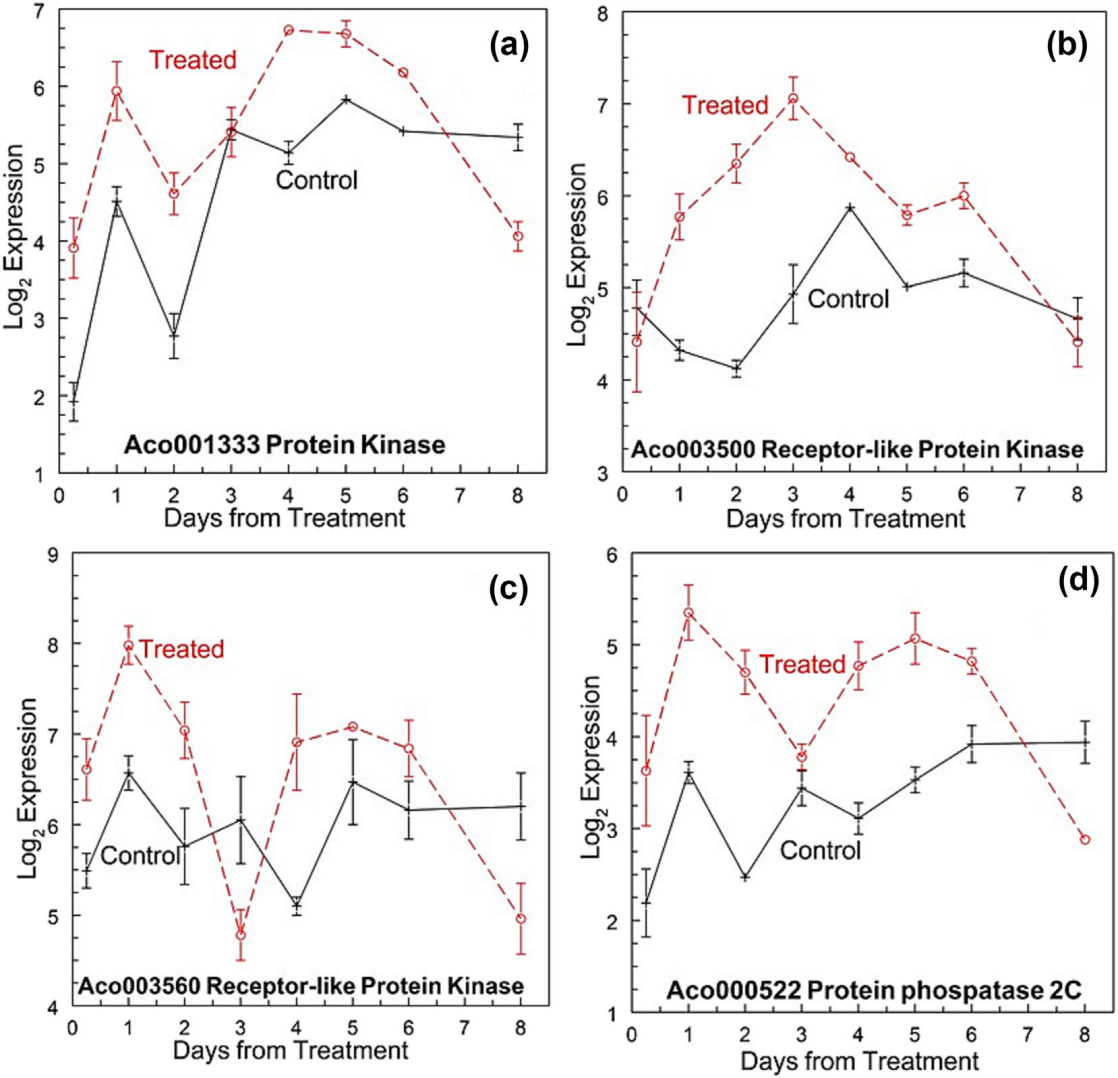
Expression of protein receptor‐like kinases and phosphatase after C2H4 treatment. (a) Aco001333 protein kinase, (b) Aco003500 receptor‐like protein kinase, (c) Aco003560 receptor‐like protein kinase, and (d) Aco000522 protein phosphatase 2C. Log_2_ means ± standard error, *n* = 3.

Protein kinase superfamily proteins were generally the highest number of genes DE both up‐ and down‐regulated (Figure SF1). On days 2 and 3, 25 and 20 protein kinase genes were expressed, respectively, and on days 1 and 4, 15 and 5 genes were down‐regulated. The protein kinase gene, Aco001333, was up‐regulated at the apex and leaf base at 6 h and then at the apex on days 1, 2, and 4 (Figure 6a). Aco003479 was up‐regulated at the apex only at 6 h and on days 1 and 3. Aco005092 was up‐regulated only at the apex on days 1 through 3, and Aco010095 was up‐regulated in the apex at 6 h and on day 1 and in the leaf down‐regulated at 6 h and up‐regulated on day 1. Aco009459 was up‐regulated at the apex on days 1, 3, 4, and 5 and down‐regulated at the leaf base on days 3 and 4. Other protein kinase genes with a similar cis‐motif (Aco005313, Aco008045, Aco013545, Aco014629, Aco018063, and Aco024439) were not DE in either tissue at all sampling times.

Receptor‐like kinase (Aco003500) was up‐regulated from days 1 to 3 compared with the control (Figure 6a). A second receptor‐like kinase (Aco003560) was up‐regulated in the first three samples and then had an expression level similar to the control apex (Figure 6c). Two acid phosphatases (Aco001653 and Aco029542) had similar up‐regulation between the untreated control and treated plants at the apex and leaf bases. Protein phosphatase 2C (Aco000522) was up‐regulated at the apex on days 1, 2, 4, and 5 and down‐regulated at the leaf base at 6 h (Figure 6d).

#### 3.4.6 DNA modification, histones, and histone interactions

**FIGURE 7:**
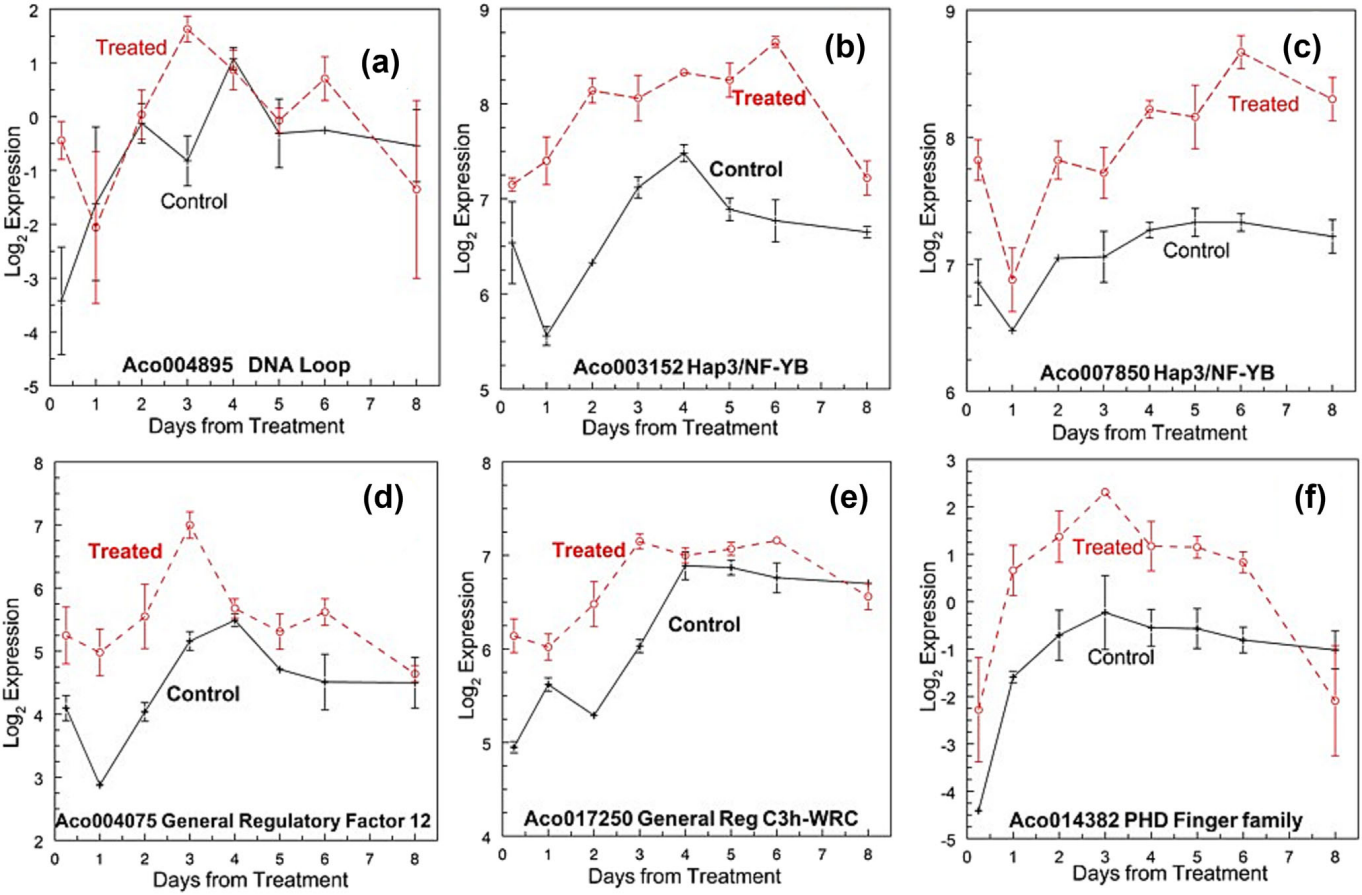
Histone and growth regulatory factors. (a) Aco004895 DNA loop protein, (b) Aco003152 Hap3/NF‐YB, (c) Aco007850 Hap3/NF‐YB, (d) Aco004075 growth regulatory factor 12, (e) Aco017250 growth regulatory factor C3h‐WRC/GRF, and (f) PHD ZN‐finger family protein. Log_2_ means ± standard error, *n* = 3.

Six hours after ethephon treatment, a predicted SWIB.MDM2 domain containing a protein involved in the formation of DNA gene loops, Aco004895, increased nearly three times in both the leaf bases and the apex from a very low level (Figure 7a). There was a second peak on day 1 at the apex, but it declined in the leaf base. No changes were observed in the predicted Zn^2+^‐dependent histone deacetylases at the apex, although changes were observed in the leaf bases (Aco000593 and Aco002180) 6 h and days 1 and 2, respectively. Two genes predicted to be involved in RNA‐directed DNA methylation (Aco004257 and Aco012515) did not show marked changes in the apex or leaf bases (Table ST2). Two DNA (cytosine‐5)‐methyltransferase (Aco006129 and Aco007653) were negatively regulated at the treated apex with little change in leaf bases (Table ST2). Similarly, four DNA topoisomerases (Aco003061, Aco004200, Aco008016, and Aco013563) showed little or no change in expression.

The expression of histone H2B and HAP3 (Ac0001475, Aco003152 [Figure 7b], Aco003157, Aco010959, and Aco014210) were up‐regulated within 6 h after ethephon treatment. Histones 1–3 (Aco000204 and Aco001050), histone H2A‐12 (Aco014218), and histone H3 (Aco007850 [Figure 7c] and Aco018209) were up‐regulated at the apex 1 day after treatment, although HAP3 histone (Aco012142) was down‐regulated on day 1 and histone H4 (Aco018376) and histone H1–3 (Aco031193) on day 2. Five histone H4 genes were up‐regulated on day 6 in the apex (Figure SF1).

Three growth regulatory factors (C3h‐WRC/GRF) associated with histones were up‐regulated at the apex in 6 h (Aco004075 [Figure 7d], Aco017250 [Figure 7e], and Aco023268) (Figure 7), and three more increased on day 1 (Aco009479, Aco015755, and Aco020046). A plant homeodomain (PHD) protein with a histone tail (Aco014382) increased in 6 h and was expressed at a higher level than the control for all but the sampling on day 8 (Figure 7f). Homeobox Knotted‐1‐like genes (Aco004983 and Aco015873) showed similar up‐regulation from day 1 forward, only at the apex (Table ST2). Both were DE from days 2 to 8 with Aco015873 from day 1. A response regulator (14‐3‐3) (Aco018444) tended to be lower at all samplings but not DE. On days 2 and 3, 25 and 20 pentatricopeptides genes were up‐ and down‐regulated, respectively, and potentially involved in organelle RNA processing (Figure SF1).

Endoreduplication was not indicated at the apex with Aco004998 cyclin not DE and a decline in Aco003499 cyclin‐dependent kinase G‐2 only on day 1. A decrease (days 3) was observed in the cyclin‐dependent kinase inhibitor Aco010247 (Siamese) (Table ST2).

#### 3.4.7 Flowering time and floral transition

The Circadian Timekeeper homolog in pineapple (Aco019534) was not DE. The GIGANTEA‐like gene (Aco014347) believed to be clock‐controlled and involved in flowering timekeeping in some systems was down‐regulated at the pineapple apex and was generally less than control, especially 3 and 4 days after ethylene treatment (Table ST2).

The expression of homologs of TERMINAL FLOWER (TFL) (Aco016718 and Aco031443) began to increase at the apex after day 2 and was DE on days 6 and 8 without a similar pattern in the leaf base (Table ST2). The expression of the TEOSINTE‐BRANCHED I homolog (TCP) (Aco015741) decreased faster in the control than in the treated apex and was DE on day 3 only in the apex. The FRIGIDA‐like homolog (Aco015042) increased at the apex after day 1 and was DE at the apex on day 2 but at no time at the leaf base.

Three EARLY FLOWERING‐like homologs (ELFs) showed different differential expression patterns. Aco005852 showed a decrease on day 8 at the apex with no change at the leaf base. Aco009476 at the apex and leaf base was up‐regulated at 6 h and on day 1 after treatment. The third ELF gene (Aco030106) was only DE at the apex, being up‐regulated at 6 h and 1 day after treatment and down‐regulated on day 3 (Table ST2).

Two WUSCHEL homologs (Aco006233 and Aco015382) showed a similar pattern of upregulation at the apex only within 6 h after treatment (Table ST2). At the apex, the response of Aco015382 was more pronounced, DE from day 2 through day 8 only at the apex, and Aco006233 was DE from day 1 through day 3. The three TOPLESS‐related genes (Aco006421, Aco018149, and Aco018150) that are corepressors in the transition to flowering in model systems, interacting with CO and FT, were not DE.

#### 3.4.8 CONSTANS‐like, SOC1, and unusual floral organs

**FIGURE 8:**
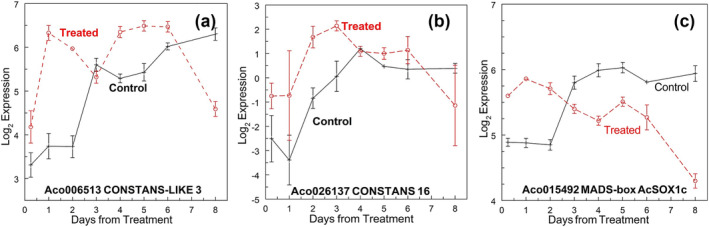
Gene related to the apical meristem from vegetative to floral transition. (a) Aco006513 Zn‐finger CONSTAN‐ like 3, (b) Aco026137 CONSTANS‐like 16, and (c) Aco015492 MADS‐box SUPPRESSOR OF CONSTANS OVEREXPRESSION 1 (AcSOC1c). Log_2_ means ± standard error, *n* = 3.

The CONSTANS‐LIKE 16 zinc finger protein (Aco003091, Aco006513 [Figure 8a], and Aco026137 [Figure 8b]) showed different patterns. Aco003091 was up‐regulated at 6 h at the apex and down‐regulated on day 3, and up‐regulated in the leaf base on day 2. Aco006513 was up‐regulated at the apex on days 1, 2, 4, and 5 and down‐regulated on day 8 (Figure 8a), and up‐regulated in the leaf base on days 3 and 4. Aco0026137 was up‐regulated at the apex on days 2 and 3 and at the leaf bases on days 3 and 4.

The closest pineapple homolog (Aco015492–AcSOC1c) to SOC1 (SUPPRESSOR OF OVEREXPRESSION OF CONSTANS1), a MADS‐box transcription factor, was negatively regulated on day 8 at the apex only (Figure 8c). The other reported AcSOC1 genes (Aco0013229–AcSOC1a, Aco016643–AcSOC1b, Aco017449–AcSOC1e, and Aco030142–AcSOC1d) were not DE at the apex or leaf bases in this early stage of the apex transition. AcSOX1a to AcSOX1d were expressed at high levels at all stages (Table ST2).

The UNUSUAL FLORAL ORGAN homolog in pineapple (Aco008339) was expressed at low levels at the treated apex, untreated apex, and at the leaf base declining after day 3 with the treated declining at a faster rate, although not DE.

**FIGURE 9:**
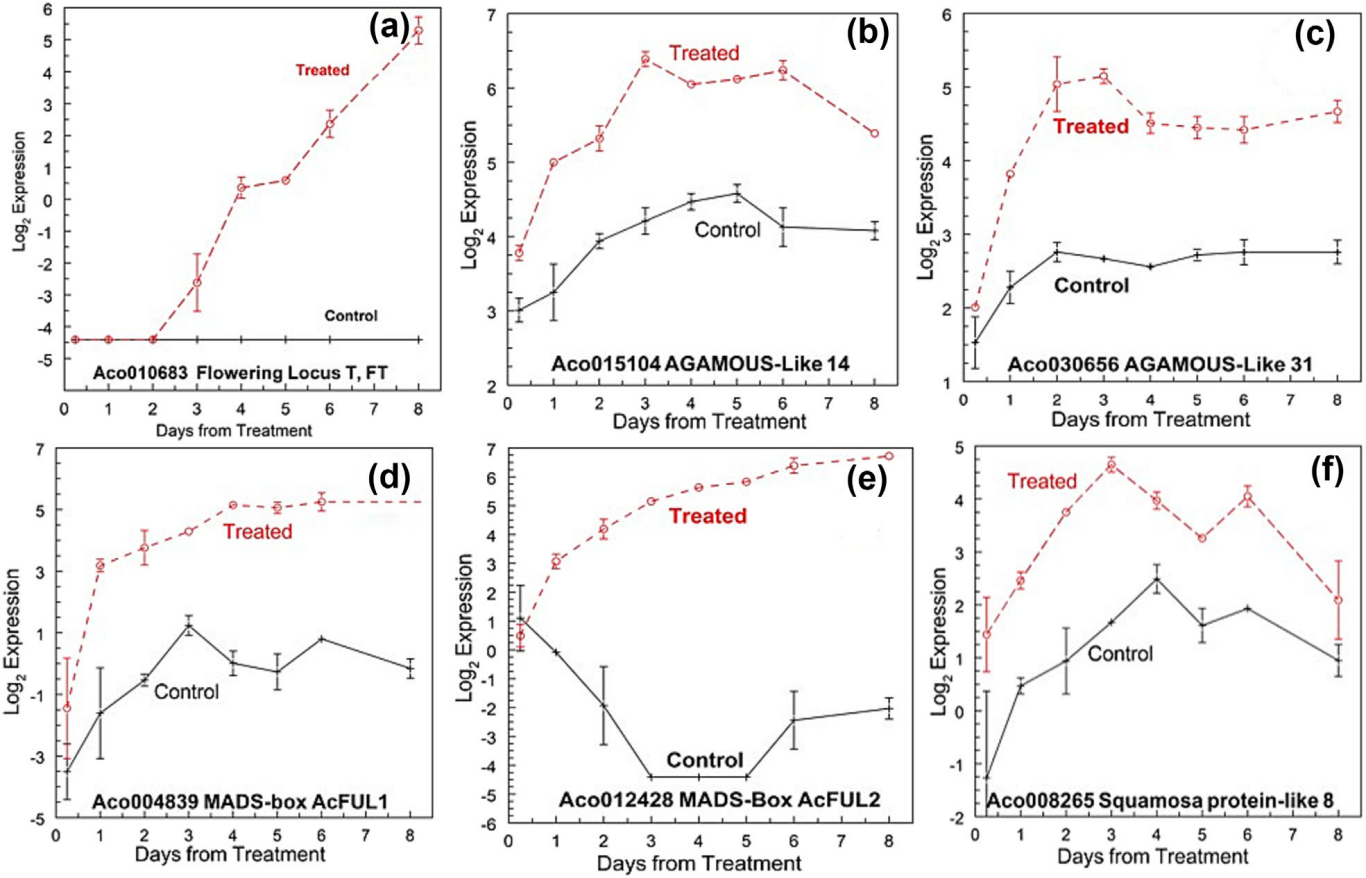
Expression of genes related to the floral meristem. (a) Aco010683 Flowering Locus T (FT), (b) Aco015104 MADS‐box AGAMOUS‐like 14, (c) Aco030656 MADS‐box AGAMOUS‐like 31, (d) Aco004839 MADS‐box AcFUL1, (e) Aco012428 MADS‐box AcFUL2, and (f) Aco008265 Squamosa protein‐like 8. Log_2_ means ± standard error, *n* = 3.

FLOWERING LOCUS T (FT) (Aco010683 [Figure 9a] and Aco010684) began to increase at the apex only on days 2 or 3, respectively, and then in the treated was DE on days 6 and 8, respectively, with little expression in untreated control plants. FT expression of FT for both genes at the leaf bases was very low and showed little expression after the ethephon treatment.

Two genes described in the literature as the flowering repressor Flowering Locus C (FLC) Aco015104 (Figure 9b) and Aco019039 increased within 6 h after ethephon treatment, differentially up‐regulated from day 1 onward, with no differential expression at the leaf bases. However, BLASTP of these two MADS‐box transcription factors showed greater identity with AGAMOUS‐like 14 and CAULIFLOWER A‐like an AP1 paralog, respectively. Another AGAMOUS‐like 31 gene (Aco30656) was similarly DE from days 1 to 8 only at the apex (Figure 9c). This confirmed the failure of others to detect FLC in pineapple and its possible loss from the genome (Zhang, Fatima, et al., 2020).

The MADS‐box AcFUL genes (Aco004839–AcFUL1 [Figure 9d] and Aco012428–AcFUL2 [Figure 9e]) were both up‐regulated at the apex only, from days 1 to 8. The SEP4 gene (Aco017563), although expressed at a higher level in the treated apex, was at an overall low expression level; however, it was not DE.

Two Squamosa‐like binding proteins (Aco08265 [Figure 9f] and Aco012822) were up‐regulated at the apex only from day 1 through day 8. Two proteins in the NAC domain (Aco000744 and Aco011880) were DE at the apex and up‐regulated. Aco000744 was DE on days 1 through 4 and Aco012822 on days 2 and 3 only (Table ST2).

The increase in FLORICAULA/LEAFY (Aco019058) was observed in both treated and untreated control plants, the increase DE on days 2 and 3 (Table ST2). At the leaf base, both untreated and treated tended to decline from their low levels. Another predicted FLORICAULA‐LEAFY‐like pattern (Aco019059) was not DE at the apex or leaf base. Although the PISTALLA gene was expressed at a lower level than the control at the apex, it was not DE at either the apex or the leaf base. Sterile APETALA (Aco002301) was up‐regulated and DE at the apex only on day 2.

### 3.5 Expressed transcription factors DNA binding motifs

Forty‐one TFs were selected that were related to ethylene responses and involved in potential flowering, had known homology, and were expressed soon after ethephon treatment, and 21 were associated with at least one sequence‐specific binding motif (Table ST3). Twenty‐two candidate motifs were inferred, and a single transcription factor could potentially have one or more binding sequences (Table 2).

**TABLE 2:** Selected pineapple transcription factors expressed after flower induction, trans‐binding motifs, and Pfam domain. The best predictions in footprintDB were selected in *Arabidopsis thalaiana.*

Gene name	Blast e‐value	Interface similarity	footprinDB PWM/Consensus	Pfam domain
Aco000347.1	1E^−29^	7/7	MA0975.1: s **C G CC G CC**	PF00847 AP2 domain
Aco001697.1	1E^−115^	4/4	M0680_1.02: ga **A T G** w **A** y **C T** g M0370: ry **G T C** y **A G** r **T** t **C A** ww	PF04873 Ethylene insensitive 3
Aco001844.1	3E^−34^	7/7	RAP2.3: rg **C G CC G C** ma	PF00847 AP2 domain
Aco002115.1	2E^−56^	7/13	M0821_1.02: y **G T A C GG** wm	PF12041 Transcriptional regulator DELLA protein N terminal
Aco004839.1	7E^−84^	8/8	MA0940.1: my **AAAAA** wr **G AAA**	PF00319 SRF‐type TF
Aco005324.1	4E^−32^	7/7	ERF1/ERF2/ERF5: **A G CC G CC A** ATERF1_2: yt **G CC GG C** ar	PF00847 AP2 domain
Aco005453.1	6E^−22^	4/6	M0821_1.02: y **G T A C GG** wm	PF12041 Transcriptional regulator DELLA protein N terminal
Aco005770.1	7E^−16^	4/7	MA0988.1: hg **C A C G T G** cd	PF00249 Myb‐like DNA‐binding domain
Aco006074.1	1e^−33^	7/7	MA0975.1: s **C G CC G CC**	PF00847 AP2 domain
Aco006233.1	5E^−12^	‐	M0422: **A** r **TT AA TT A** rt	PF00046 Homeobox domain
Aco010600.1	2E^−54^	7/7	ERF1: rs **C G CC G CC** a MA0567.1: m **G CC G CC** a	PF00847 AP2 domain
Aco012428.1	2E^−79^	7/8	MA0940.1: my **AAAAA** wr **G AAA**	PF00319 SRF‐type TF
Aco014268.1	8E^−34^	7/7	M0061: kygr **C GG C GG** m **C** gwg UN0363.1: cwc **C** t **CC G CC G** cc	PF00847 AP2 domain
Aco015104.1	1E^−27^	7/8	MA0940.1: my **AAAAA** wr **G AAA**	PF00319 SRF‐type TF
Aco015335.1	4E^−61^	4/4	M0680_1.02: ga **A T G** w **A** y **C T** g M0370: ry **G T C** y **A G** r **T** t **C A** ww	PF04873 Ethylene insensitive 3
Aco015382.1	4E^−22^	8/8	6ryi_DE: **A T C A** cg **T G A** 6ryl_DE: **T** y **AA T G C G TT** s **T** M0436: **T G A** w **T G A** w **T G** a M0447: **T C A** w **T C A** w **T** y **A**	PF00046 Homeobox domain
Aco017254.1	9E^−52^	20/20	MA1038.1: d **GG T A GG T** ara	PF00249 Myb‐like DNA‐binding domain
Aco017455.1	5E^−17^	3/3	M0468: s **C G A** a **AA** wwt **C GG** ar	PF03195 Protein of unknown function DUF260
Aco017871.1	3E^−125^	4/4	M0680_1.02: ga **A T G** w **A** y **C T** g M0370: ry **G T C** y **A G** r **T** t **C A** ww	PF04873 Ethylene insensitive 3
Aco018980.1	2E^−37^	9/9	ORA47: cr **CC G A CC A** a ORA47_2: k **G C G CC G** m **C** t MA1048.1: r **CC G A CC A**	PF00847 (AP2 domain)
Aco019059.1	5E^−24^	2/4	2vy1_A: **T GG T** nnn **T A**	PF01698 (Floricaula/Leafy protein)
Aco031731.1	4E‐50	7/11	M0821_1.02: y **G T A C GG** wm	PF12041 Transcriptional regulator DELLA protein N terminal

TF binding sites (TFBSs) were not uniformly distributed in the upstream promoter region. However, they tended to lie in the vicinity of the transcription start site (TSS), exhibiting a clear peak within the region from 400 to −200 bp, and then drop progressively. We did not observe binding sites on the downstream side of the TSS (0, +200pb).

To understand the positional binding preference of each TF family, the motifs were grouped into eight families based on the Pfam domains of their corresponding TFs (Figure SF4). All AP2‐motifs shared the same positional distribution profile, being concentrated within −400, −200 bp, which denoted an AP2‐binding preference within this region. Although Myb and DELLA showed a single central peak around the interval [−800, −600 bp], others such as homeobox, SRF, and EIN3 binding sites exhibited multiple peaks upstream of the TSS that can denote multiple binding preferences.

### 3.6 Transcription factors following induction

**FIGURE 10:**
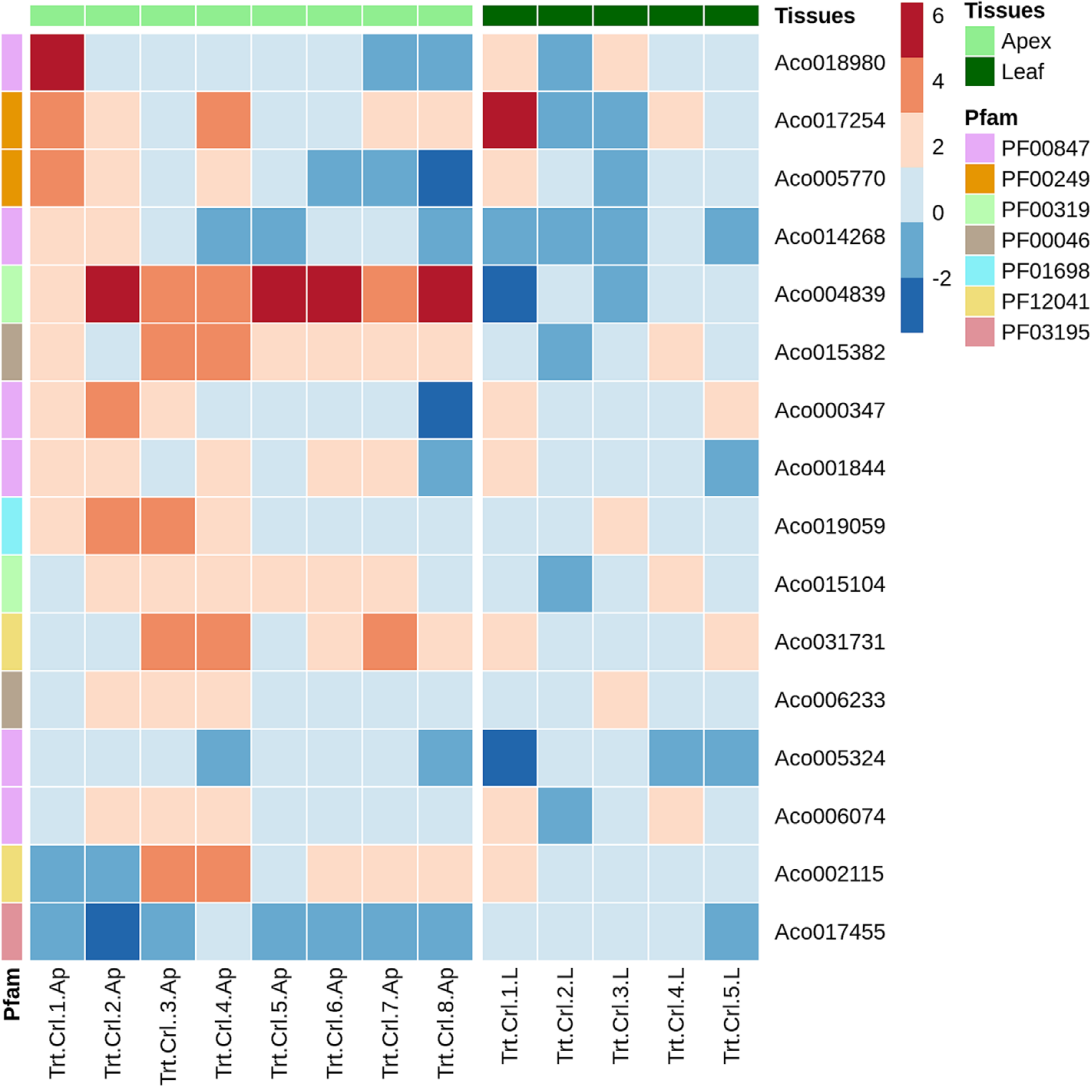
Expression profile of selected transcription factors associated with pineapple flowering in the apex and leaf bases and their predicted binding domain. Blue and red squares indicate the levels of gene expression. The *x*‐axis corresponds to the control and treated tissues (apex and the leaf), and the *y*‐axis corresponds to the flowing TFs grouped by their Pfam domain. The row color bar refers to the Pfam domain, and the column color bar corresponds to the tissue type (see the color key).

The target genes for the selected transcription factor binding motifs (Table 2) were grouped into eight broad categories according to function (Figures 10 and SF3). Higher expression occurs in the first days after ethephon treatment in transcription factors, protein turnover, kinases, phosphatases, and plant growth regulators. A secondary peak was evident 3 to 4 days after ethephon treatment in a narrow range of target genes. Target genes expressed at the leaf base tended to follow a different expression pattern from that of the apex. A similar pattern was found in carbohydrate metabolism and general metabolism to transcription factors, and redox metabolism and stress‐related genes tended to cluster a few days after treatment (Figure SF4). A potential transcription repressor that included the regulation of GA20‐oxidase and thus GA biosynthesis, the OVATE genes, were up‐regulated on day 0 (Aco004275) and Aco015523 from days 4 to 8 at the apex (Results SR1). At leaf bases, an OVATE gene (Aco012538) was up‐regulated on days 0 to 2. Other OVATE genes were generally negatively regulated.

### 3.7 Small RNA expression

One hundred and ninety miRs were expressed at various levels in one or more replication or apex sampling stages or in the treated or control. When filtered and normalized, 76 miRs were retained in 30 families (Table ST6) from differential expression analysis and temporal clustering. Single members of 10 miR families were DE (Table ST8) at different sampling stages and cross‐listed with published pineapple miR targets (Zheng et al., 2016).

The two miRs often associated with vegetative phase change and flowering control in angiosperm miR156 and miR172 were not DE at any stage or tissue. Two members of miR 156 were expressed at a very low level, and one (miR172a) of the four members of miR172 expressed was at a higher level and tended to show lower expression in the treated apexes during the latter sampling times (Table ST8).

**FIGURE 11:**
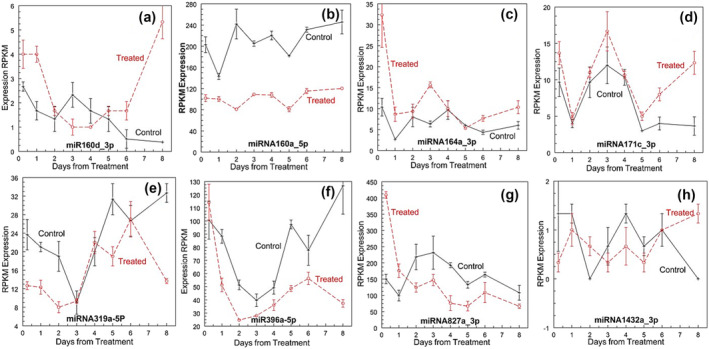
Differentially expressed miRNA after ethylene treatment. (a) miRNA160d_3p, (b) miRNA160a_5p, (c) miRNA164a_3p, (d) miRNA171c_3p, (e) miRNA319a_5p, (f) miRNA396a_5p, (g) miRNA827a_3P, and (h) miRNA1432a_3p. RPKM means ± standard error, *n* = 3.

MiRs that showed up‐ or down‐regulation, such as miR160d (Figure 11a), miR160a (Figure 10b), miR164a (Figure 11c), miR171c (Figure 11d), miR319a (Figure 11e), miR396a (Figure 10f), miR827a (Figure 11g), and miR1432a (Figure 11h), did not show a consistent pattern or agreement with the expression pattern of the predicted target genes (Table ST8). Up‐regulation of miR396c, 6 h after treatment, could be related to down‐regulation of seven DE target genes on day 1: subunit of the condensin complex subunit, Aco011167 Auxin Efflux, translational activator GCN1, two callose synthases, and protein of unknown function. An unknown protein with DUF 3049 was up‐regulated from days 2 through 8 at the apex only when miR 396 was less consistent than the control. No miR was differentially regulated on days 2, 4, 5, and 6 after treatment. There were some parallels between the miR expression patterns of the apex and potential targets DE in the leaf bases (Table ST8).

## 4 DISCUSSION

The induction of flowering in pineapple proceeds in phases, the first phase being the change from the vegetative meristem to the floral meristem. In pineapples, this morphological change can be observed 7 days after induction, with a change from leaf primordia to the first floral bracts (Bartholomew, 1977; Espinosa et al., 2017; Liu et al., 2011). In the second phase, flower primordia first appear on day 11 on the axils of the bracts and continue to initiate until about 200 flowers are formed, each subtended on a bract, on day 34 after induction (Bartholomew, 1977; Chen et al., 2019). The last phase is the open flower stage that starts about 90 days after induction. The phase 1 meristem morphological changes are associated with a peak in 1‐aminocyclopropane‐1‐carboxylic acid (ACC) in the leaf 2 days after treatment with naphthalene acetic acid (NAA) (Botella et al., 2000) and an ethylene peak 4 to 8 days after flower induction (Lin, Maruthasalam, et al., 2009; Liu et al., 2011). These results are supported by AcACS2 induction in the meristem during flower induction, while silencing and suppression of AcACS2 in transgenic pineapple plants result in significant delay in flowering (Trusov & Botella, 2006; Wang & Paull, 2018). Therefore, AcACS2 may be the main contributor to the promotion of pineapple flowering (Trusov & Botella, 2006). However, in our results, AcACS2 (Aco000276) (Figure 3b) was not DE in the first 8 days after ethylene treatment, and AcASC1 (Aco015517) was negatively regulated at the apex 2 to 3 days and again 6 and 8 days after treatment (Figure 3a). This suggested that AcASC2 acts later in the vegetative‐to‐floral transition process. This is partially supported by the latter down‐regulation of ACO genes being similarly down‐regulated in the apex and/or leaf base at days 2 and 3 and up‐regulated from days 4 through 8. The same ACO gene (Aco015240) had previously been reported to be up‐regulated 8 days after ethylene treatment (Liu et al., 2021; Liu, Liu, et al., 2018). Similarly, other genes associated with the ethylene response pathway: reception, protein kinase (CTR1) (Figure 3d), and EIN2 (Figure 3e) were not DE. EIN3/EIL (Aco017871) (Figure 3f) was negatively regulated at the apex from days 3 to 8 similar to that reported (Liu et al., 2021).

Some of the first changes in gene expression were in ethylene‐responsive transcription factors (ERTF) (Figure 4g–j and Table 1). ERTFs were expected to be the first step in the conversion from vegetative to floral meristem in phase I. Five ERTFs have been reported to increase 8 days after treatment (Liu et al., 2021; Liu, Liu, et al., 2018), although the absence of a control does not allow for determination of differential expression. One of these reported ERTFs (Aco001844) was up‐regulated only at the apex, from 6 h and the first 4 days after treatment, together with (Aco001318, Aco002824, and Aco16349) (Table 1). The four reported ERTFs (Liu, Liu, et al., 2018), Aco001600, Aco009511, Aco012860, and Aco017803, were down‐regulated at the apex from day 3 or only on day 8.

Our results indicated dramatic up‐ and down‐regulation of auxin influx and efflux transporters at the apex and leaf base (Table ST2). ARF (Aco009671) was up‐regulated at the apex only 2 and 3 days after treatment, possibly involved in the reshaping of the apex in the floral transition (Liu et al., 2021), and an auxin‐associated dormancy factor (Aco009671) decreased. These changes were consistent with an earlier finding that indole acetic acid (IAA) levels at the apex are lower than in untreated plants from days 4 to 16, whereas IAA in the leaf base increases in the treated plants after day 4 (Liu et al., 2011), suggesting possible auxin redistribution in the apex. The MATE efflux proteins that could be involved in the transport of ABA and auxins and petal development (Liu, Liu, et al., 2018) were up‐regulated at the apex of the pineapple, although this response may involve ARF. Auxin (IAA) levels at the apex of the pineapple and leaf bases increase 16 days after ethephon treatment, whereas CK zeatin increased in both the base of the apex and the leaf 8 days after treatment (Liu et al., 2011). The same researchers found a peak in 2‐isopentyl adenine in the apex 4 and 8 days after treatment. CK‐related genes only showed a downward trend in a CK response regulator (Aco002961). At this early stage of the vegetative to floral transition, it is possible that the “antagonistic” activity between auxin and cytokinin (Barton, 2010; Hnatuszko‐Konka et al., 2021; Kurepa et al., 2019; Merelo et al., 2022; Wybouw & De Rybel, 2019) did not play a critical role in the vegetative transition of pineapple to the floral meristem transition.

GA synthesis and signaling have been shown to regulate various aspects of flowering in Arabidopsis through DELLA genes (Bao et al., 2020). However, the potential crosstalk of GA with JA (Osadchuk et al., 2019) does not appear to play a role in the early steps in the vegetative–floral transition based on gene expression data. Furthermore, GA3 levels decrease within 4 days after treatment in both the leaf and the apex and are lower than in untreated until day 28 (Liu et al., 2011). The other stress‐related plant growth regulator abscisic acid is elevated within 4 days of treatment and then decreases (Liu et al., 2011), and the two genes that potentially encode stress‐ripening proteins from abscisic acid increased from day 3. Other genes related to stress: abundant genes for LATE EMBRYOGENESIS ASSOCIATED (LEA), nodulin‐related, heat and dehydration stress, and Remorin were expressed early (days 1 and 2) or in later stages (6 days) or down‐regulated (Table ST2). Changes in these stress‐related genes occurred at both the apex and leaf base.

The JA synthesis gene, allene oxide synthase (Aco008572), increased rapidly after treatment without a change in the potential COI JA receptor (Figure 4a,b). The JA response genes were up‐regulated shortly after treatment. Liu et al. (2021) reported a decrease in JA content and an increase in a gene (Aco005530) annotated as JA‐amino synthetase, which we characterize here as an auxin response gene from the GH3 family that was down‐regulated only in the leaf base on day 2. JA receptor mutations and overexpression of JAZ repressors in rice and Arabidopsis result in early flowering phenotypes (Yang et al., 2012) and support the finding that exogenous application of methyl jasmonate leads to earlier flowering (Huang et al., 2022; Zhao et al., 2021). Repression of FLOWERING LOCUS T (FT) expression is a potential mechanism through which JA receptors could delay flowering (Huang et al., 2022; Zhai et al., 2015). These results suggest that the COI‐mediated JA pathway delays Arabidopsis and rice. However, in pineapple, we observed an increase in JA synthesis and response genes immediately after treatment. The various roles in both plant stress responses and the floral transition can act as a hub to regulate the balance between these two processes, as signaling pathway genes have similar gene components (Huang et al., 2022; Wang et al., 2019; Zhao et al., 2021) and potential GA crosstalk (Bao et al., 2020; Osadchuk et al., 2019), although not through changes in a MYC transcription factor (bHLH) in the early stages of the floral apex transition expressed at low levels and not DE.

Kinases are involved in a wide range of developmental functions, including hormone responses and cell morphogenesis (Becraft, 2002; Wang & Gou, 2020; Wang, Hsu, et al., 2020). Several of these kinases were up‐regulated at the apex of the pineapple after treatment (Figures 6d and SF1). The timing of up‐regulation varied with the kinases. A receptor‐like kinase (Aco003560) was up‐regulated in the first three samples and another (Aco003500) (Figure 6b) was up‐regulated 1 day later, suggesting specific responses, although the substrate or regulatory pathway was unknown.

Histone deacetylation regulates gene expression (Berr et al., 2012) through histone deacetylases (HDA). Within 6 h after treatment, a pineapple homolog (Aco016684) is negatively regulated in the leaf bases and another (Aco015229) 1 day and Aco003725 2 days after treatment at the apex, and (Aco016700) is up‐regulated in the apex on day 2. HDA in Arabidopsis is induced by JA and pathogens (Wu et al., 2008; Zhou et al., 2005), and down‐regulation by antisense RNA and mutations or overexpression cause delayed flowering under long‐day conditions (Ning et al., 2019; Yu et al., 2011; Zhou et al., 2005). Histone acetyltransferases are also involved in gene regulation (Boycheva et al., 2014), and one (Aco007580) (Figure 7c) was DE at the apex at 6 h. A SWIB/MDM2 domain superfamily protein (Aco004895) potentially involved in the accessibility of the DNA remodeling complex that altered accessibility (Sacharowski et al., 2015) was up‐regulated on day 3 at the apex (Figure 7a). Dramatic changes in histones (H1–3, H2A, H2B, H3, and H4) were especially up‐regulated at the leaf bases at 6 h and then some at the apex on day 1 and day 2 (Aco001050, Aco10964, Aco0001473, Aco001475, Aco010959, and Aco018209) in H2A, H2B, and H3 (Figure 7). These data support dynamic changes that occur in DNA accessibility and new gene expression patterns (Borg et al., 2021; Pfluger & Wagner, 2007). Histone H2A is diverse and complex and plays critical roles in development (Borg et al., 2021; Lei & Berger, 2020) and in Arabidopsis modulates the flowering response (Jarillo & Piñeiro, 2015; Searle et al., 2006). These histones can also be modified by histone‐lysine N‐methyltransferases that are down‐regulated at the apex on day 1, further changing the expression of the new gene. Trimethylation of H3 in FLOWERING LOCUS C chromatin has been reported in cabbage bolting (Fu et al., 2020). Endoreduplication is a common feature during fleshy fruit development (Tourdot et al., 2023); although not detected at this early stage of fruit development, it potentially occurs during fruit early flowering and fruit expansion (Mao et al., 2018).

Growth regulatory factors (GRFs) interact with other transcriptional coactivators, though not mandatory, and DNA (Fonini et al., 2020; Kim & Tsukaya, 2015) to control plant growth, including flower organogenesis (Liu et al., 2014), and interact with auxin response factors and miR396 (Beltramino et al., 2021). Three GRFs were up‐regulated within 6 h after treatment and another three on day 1 (Figure 7d,e), suggesting a role in remodeling of the apical meristem of the shoot. The protein–protein interaction involving 14‐3‐3 proteins and phosphorylation can act as a negative regulator of rice flowering (Taoka et al., 2011) was not DE in pineapple. A plant homeodomain protein (PHD) (Aco014382) was higher at 6 h and increased on days 2 through 3 at the apex (Figure 7f), whereas other PHD proteins were up‐ or down‐regulated at 6 h at the leaf bases (Table ST2). Homeodomain proteins are involved in responses to abiotic stress and plant growth and development (Alam et al., 2019; López‐González et al., 2014; Searle et al., 2006) and are co‐expressed with auxin and ethylene response factors (Müller & Munné‐Bosch, 2015; Roosjen et al., 2017). The PHD protein with its histone‐binding tails has been shown to play a role in the regulation of plant development (Zhao et al., 2018) and flowering time potentially by improving the binding of BAH transcription regulators to trimethylated histone H3 (Qian et al., 2021). Other homeobox proteins (Mukherjee et al., 2009), such as WOX, showed significant up‐ and down‐regulation in leaf bases, with some up‐regulated at the apex from day 1 or 2 forward (Aco012965, Aco015382, and Aco023084) with Aco008700 down‐regulated on days 1 and 2. In the same superclass, HD‐Zip (Aco001263, Aco002750, and Aco012846) were initially up‐regulated from 6 h in the leaf base and only at the apex from day 1 onwards. None of the nine Bel1‐like homeobox genes was DE. The WUSCHEL mobile homeobox (WUS) is critical for the maintenance of the shoot by controlling cell fate (Barton, 2010; Kitagawa & Jackson, 2019; Mayer et al., 1998). Two WUSCHEL homologs (Aco006233 and Aco015382) were up‐regulated in the apex only within 6 h, and Aco015382 was DE from days 2 to 8, potentially involved in the modification of the shoot apex. The WUS transcription factor relies on a feedback loop with CLAVATA3 (Fletcher et al., 1999; Prusinkiewicz et al., 2022). CLAVATA3/ESR‐related (Aco026242) was not DE at the apex of the pineapple and was expressed at a lower level at the leaf base than at the apex. Meristematic cells require the expression of the KNOX gene SHOOTMERISTEM LESS (Long et al., 1996). Two KNOTTED‐1‐like genes (Aco004983 and Aco015873) from days 2 and 1, respectively, are up‐regulated; KNOTTED‐1‐like genes have been implicated in maintaining meristems (Hay & Tsiantis, 2010) and wheat flowering with GRFs (Kim et al., 2019).

Photoperiodic regulation of flowering occurs in many plants (Amasino, 2010; Jin et al., 2018; Liu et al., 2013; Putterill et al., 1995; Yano et al., 2000) such as Arabidopsis, rice, and maize. Pineapple is also a quantitative short‐day plant (Friend & Lydon, 1979). Specific molecular components in this photoperiodic response pathway are conserved (Fu et al., 2015; Hecht et al., 2005; Liu et al., 2013). The transcription factor CONSTANS (CO) proteins are involved in the regulation of FLOWERING LOCUS T (FT), TERMINAL FLOWER (TFL), and related proteins (Amasino, 2010; Corbesier et al., 2007; Liu et al., 2013; Putterill et al., 1995; Song et al., 2010). CO acts as a central gene that integrates the flowering network (Matsoukas, 2015; Shim et al., 2016). Interestingly, Zhang, Pan, et al. (2020) did not report CO paralogs in the pineapple genome in their analysis of the MADS‐box genes and suggested that they had been lost. We predicted 20 CONSTANS‐like proteins with the CCT domain (*CO*, *CO*‐like, *TOC1*) (Griffiths et al., 2003), of which 13 were DE in the apex or leaf bases at some sampling stage (Tables ST1 and ST2). Although different CO‐like homologs showed different patterns in differential expression, two showed upregulation in the first 6 days of sampling (Aco006513 [Figure 8a] and Aco026137 [Figure 8b]). Liu et al. (2021) reported in a different pineapple variety that two COs (Aco007020 and Aco014592) were strongly up‐regulated from days 7 to 14 outside of our sampling period. Two FTs (Aco010683 [Figure 9a] and Aco010684) were up‐regulated after day 2 until the end of the sampling on day 8 after the increase in the two CONSTANS‐like genes (Figure 8a,b). This increase in FT with similar relative expression has previously been reported at the pineapple apex (Ruan et al., 2019), although we did not see reduced expression after day 5. AcFT has been reported to be high in pineapple fruit but not in leaves with the highest level 40 days after initiation (Lv, Duan, Xie, Wei, et al., 2012). FT can cause flowering if expressed in the apical meristem of the leaf or shoot independently of CO (Amasino, 2010; An et al., 2004; Kinoshita & Richter, 2020). FT partners with FLOWERING LOCUS D (FD), a bZIP transcription factor at the apex (Abe et al., 2005; Kinoshita & Richter, 2020; Wigge et al., 2005). Nineteen bZIP TFs were regulated up or down at the apex, leaf base, or both, in general, at a single sampling time (Table ST2). Aco009751 was the only bZIP TF that was up‐regulated at the apex only on days 1 and 2. Two TFL genes (Aco016718 and Aco031443) increased similarly in parallel with the two FT genes. However, TFL is considered a gene that represses the floral transition in different meristems in a range of species (Danilevskaya et al., 2010; Ohshima et al., 1997) and is activated by SOC1, competing with FT in photoperiodic responses, although the mechanism is unclear (Périlleux et al., 2019; Zhu et al., 2020). If TFL acts in this way, increasing in parallel with FT, then its role in pineapple could be to maintain a pool of undifferentiated cells in the center of the apex for the continued development of new floral primordia, as suggested in Arabidopsis (Jaeger et al., 2013).

Flowering timing genes, such as the circadian timekeeper, were either not DE or down‐regulated. GIGANTEA (GI) (Aco014347) was negatively regulated at the apex of the pineapple 2 and 3 days after treatment without differential expression in the leaf base. GI has been shown to affect CO expression, and CO is necessary for FT expression in long‐day plants (Amasino, 2010). The TERMINAL FLOWER homologs (Aco16718 and Aco031443) increased from day 2 but were not DE until day 6, whereas the TEOSINTE‐BRANCHED homolog (Aco015741) decreased. Three homologs similar to early flowering were DE at the apex at different times after treatment. These and other genes have been implicated in the intricate balance of various internal and environmental signals to initiate vegetative to floral change and are conserved in angiosperm with modifications (Lee & Lee, 2010; Liu, Liu, et al., 2018; Srikanth & Schmid, 2011).

FLOWERING LOCUS C (FLC) are not reported in the pineapple genome (Zhang, Fatima, et al., 2020). Ruelens et al. (2013) highlighted the difficulties in identifying FLC genes with their short protein sequences and limitations of similarities searches. These limitations were addressed by Zhang, Fatima, et al. (2020) in their expanded phylogenetic study where they identified 48 MADS‐box genes (32 MIKC‐Type and 2 M‐δ type) in pineapple. Two FLCs from gene expression studies (Hu et al., 2021; Zhang, Pan, et al., 2020). These two genes Aco015104—named AcFLC1 and Aco019039—AcFLC2 increased within 6 h after treatment with ethephon, differentially up‐regulated from day 1 onwards (Figure 10) without differential expression at the leaf bases. Liu, Liu, et al. (2018) had characterized Aco015104 as AGL‐10, and Wang, Li, et al. (2020) annotated Aco019039 as AcSEP1. However, BLASTp of these MADS‐box transcription factors showed greater identity with AGAMOUS‐like 14 and CAULIFLOWER A‐like an AP1 paralog, respectively, and not FLC. The absence of FLC is in variance with other model systems in which FLC acts as a repressor in flowering timing in Arabidopsis by Histone‐3 methylation (Zhao et al., 2005). FLC acts in a dose‐dependent manner negatively regulating the expression of genes that promote flowering, such as FT and SUPPRESSOR OF OVEREXPRESSION OF CO 1 (SOC1) (Amasino, 2010; Michaels & Amasino, 1999). Bromelaids that include pineapple are considered the last family in the Poales to diversify (Bouchenak‐Khellardi et al., 2014) and therefore have a very ancestral moncot flower (Hu et al., 2021), and pineapple like *Amborella* may have lost FLC‐like genes that are sister to the SEP subfamily (Ruelens et al., 2013; Yu, Duan, et al., 2016.

The absence of this FLC repressor in pineapple means that upregulation of genes such as CO, FT, and APETALA1/FRUTFULL‐like (FUL) may be critical for pineapple flower induction. In ornamental bromeliads (*Aechima fasciata*), FT has also been implicated in flowering (Li, Wang, et al., 2016). FUL is closely related to APETALA1 (AP1) and CAULIFLOWER. In Arabidopsis inflorescence, FUL directly and negatively regulates the accumulation of AP2 and AP2‐like genes, thus maintaining WUS expression (Balanzà et al., 2018; Ferrandiz et al., 2000; Melzer et al., 2008). In pineapple, a similar AP2 (Aco006706) was down‐regulated at the leaf base at 6 h only, with numerous transcription factors that showed variable expression often up‐regulated from day 5, whereas FUL was up‐regulated from day 1 suggesting that a difference in Arabidopsis regulation is similar to AP2. STERILE APETALA (SAP)(Aco002301) was DE on day 3, whereas ULTRAPETALA (Aco014393) was not DE.

Four MADS‐box genes in Arabidopsis and rice, SOC1, SHORT VEGETATIVE PHASE (SVP), AGAMOUS‐LIKE 24, and SEPALLATA 4, act redundantly to suppress TERMINAL FLOWER1 (TFL1) in emerging floral meristems. This is critical for FUL in specifying floral meristems (Balanzà et al., 2014; Becker & Theißen, 2003; Boss et al., 2004; Callens et al., 2018; Hecht et al., 2005; Liu et al., 2013). In Arabidopsis (Balanzà et al., 2014) and wheat (Debernardi et al., 2020), FUL plays a central role in the fate of the meristem fate and interacts with SOC1 and SVP. Hu et al. (2021) describe five SOC1 genes, named AcSOC1a to AcSOC1e, in the pineapple genome, and six were reported by Zhang, Pan, et al. (2020), the exception being Aco015499. However, the only DE SOC1 was AcSOC1c (Aco15492) (Figure 8c), which was negatively regulated on day 8. AcSOC1e has been reported in the flower bud and at a lower level in the flower (Zhang, Fatima, et al., 2020). Three SVP genes have been reported in the pineapple genome (Aco002729, Aco004028, and Aco027879) (Hu et al., 2021; Zhang, Pan, et al., 2020). Only Aco004028 (AcSVP2) was up‐regulated in the leaf base at 6 h. An AGAMOUS‐like 31 (Aco030656) was up‐regulated in the apex only from days 1 to 8, and two TFL1 (Aco016718 and Aco031443) were up‐regulated on days 6 and 8, or day 8, respectively (Table ST2). Liu, Wu, et al. (2018) in their gene expression analysis reported that Aco016718 was down‐regulated at 8 days in two pineapple varieties (with neither expressed in open flowers (Zhang, Pan, et al., 2020). In most angiosperms, TFL1 and SVP suppress the transition from vegetative to floral development (Gregis et al., 2013; Wickland Daniel & Hanzawa, 2015), and FT promotes the transition. After the vegetative stage, SVP is involved in the specification of the floral meristem (Gregis et al., 2013). These two genes are homologs to phosphatidylethanolamine binding proteins that have various functions in plant growth and have recently evolved from the same gene. The expression of the TFL1 gene at the pineapple apex parallels the expression of FT, suggesting that it did not act as a repressor. Two AcFUL genes (Aco004839 [AcFUL1; AGL8; Figure 9d] and Aco012428 [AcFUL2; AGL8; Figure 9e]) have been reported in pineapple (Hu et al., 2021; Liu, Liu, et al., 2018; Wang, Li, et al., 2020) in the apical meristem and flower organs, with the first expression reported occurring 8 days after treatment. We found that both AcFUL1 and AcFUL2 were up‐regulated within 1 day of treatment and remained so until day 8 (Figure 9d,e). UNUSUAL FLORAL ORGANS (UFO) (Aco008339) were not DE at this early stage of pineapple flower formation, although they play a critical master regulator of the identity of the other flower meristem (Lee et al., 1997; Zhao et al., 2016) that interacts with LEAFY (LFY) and SEPALLATE3 (SEP3) (Lippman et al., 2008; Weigel et al., 1992). Wang, Li, et al. (2020) reported pineapple floral homeotic genes from pineapples in classes A, B, C, and E at a later stage of flower development; none were DE at this early stage of the floral transition, although six of the 11 homeotic genes were expressed.

The FLOWERING LOCUS T (FT) protein is considered a conserved mobile signal that mediates the flowering transition in all angiosperms (Pin & Nilsson, 2012; Wickland Daniel & Hanzawa, 2015). The FT protein is synthesized in the leaves and transported to the apex where it interacts with the FD. This interaction affects the binding of the TFL1 flowering repressor to chromatin (Wickland Daniel & Hanzawa, 2015; Zhu et al., 2020, 2021). In pineapple, FT was only expressed at the apex (Figure 9a) with very low levels at the leaf base (Table ST2), suggesting that FT was synthesized in the apex and transport from the leaf did not occur after treatment. TFL1 is considered a repressor of the flowering transition, although, in pineapple, its increased expression at the apex parallels FT, implying that it does not act as a repressor. These functions have undergone a change with the potential to retain their role in pineapples that of the ancestral gene, the pineapple flower being regarded as early divergent basal monocots from eudicots, and many genes retain their ancestral functions (Hu et al., 2021).

The plant‐specific SQUAMOSA PROMOTER BINDING PROTEIN LIKE (SBP) plays different roles in plant growth and development (Chen et al., 2010; Yamasaki et al., 2004), including flowering by binding to the multigene family SQUAMOSA gene (Klein et al., 1996). SBP along with FT are FLC targets (Madrid et al., 2020). SBPs are also regulated by miR156 and miR157 (Guo et al., 2008). Thirteen SBP genes of 17 were DE in pineapple, of which three were down‐regulated at the apex on day 1, two (Aco008265 [Figure 9f] and Aco012822) were up‐regulated on day 1 or 2, respectively, and Aco008265 was up‐regulated from days 1 to 6.

In our investigation, *Arabidopsis thaliana* and *Oryza sativa* were used as model species, as cis‐DNA elements within noncoding regions are poorly annotated in most plant species (Galli et al., 2020). Various experimental approaches have been used to detect and validate cis‐regulatory transcription factor binding sites (TFBS) in vivo (Christ et al., 2013; Ishimori, 2022; Koschmann et al., 2012; Rivière et al., 2022; Yu, Lin, Li, et al., 2016). However, it is worth mentioning that the precision of identifying a TFBS using cross‐species modeling depends on the degree of divergence between the species with results suggesting that the approach has predictive value (Rivière et al., 2022). We evaluated the cis regulation motifs of the DNA gene to selected DE transcription factor (TF) binding motifs potentially involved in the transition of the shoot apical meristem to floral state. TFs can act alone or cooperatively to regulate gene expression of genes (Jones & Vandepoele, 2020; Schmitz et al., 2021). The APETALA2/ethylene response transcription factor (AP2/ERTF) was considered the most important in the pineapple ethylene response; other TF factors were selected according to their expression pattern, especially in the apex tissue. Interestingly, all AP2/ERTF motifs shared the same positional distribution profile, being concentrated within −400, −200 bp that denoted a AP2/ERTF binding preference within this region and consistent with those described for TF binding (Ksouri et al., 2021). Although Myb and DELLA showed a single central peak around the interval [−800, −600 bp], others, such as the homeobox, SRF, and EIN3 binding sites, exhibited multiple peaks upstream of TSS. This could be explained by the fact that transcription factors are known to recognize multiple DNA binding sequences so that they can have multiple binding preferences (Inukai et al., 2017) and undergo changes in specificity during evolution such as LEAFY (LFY) (Sayou et al., 2014).

MiR156 and miR172, which are commonly associated with playing a role in flowering control (Aukerman & Sakai, 2003; Mathieu et al., 2009; Teotia & Tang, 2015; Zhu & Helliwell, 2010), were not DE at the pineapple apex. The conserved miR396 was up‐regulated and has been associated with the regulation of genes for the identity of the expression in Arabidopsis, barley, rice, and maize (Smoczynska & Szweykowska‐Kulinska, 2016; Yang et al., 2009). MiR396 targets (Zheng et al., 2016) were projected to be auxin efflux carrier, GCN1 translational activator, a condensin complex subunit, callose genes, and some unknowns. The GCN1 target was the most interesting because it has been associated with flower organ type and delayed flowering time (Baucher et al., 2013; Cui et al., 2021) through regulation of genes at the translation level and therefore plays a role in pineapple flowering. In Arabidopsis, GCN1 is known to target the growth‐regulating factor (GRF) gene family (Baucher et al., 2013), some of which are up‐regulated in pineapple (Figure 7d,e,f).

**FIGURE 12:**
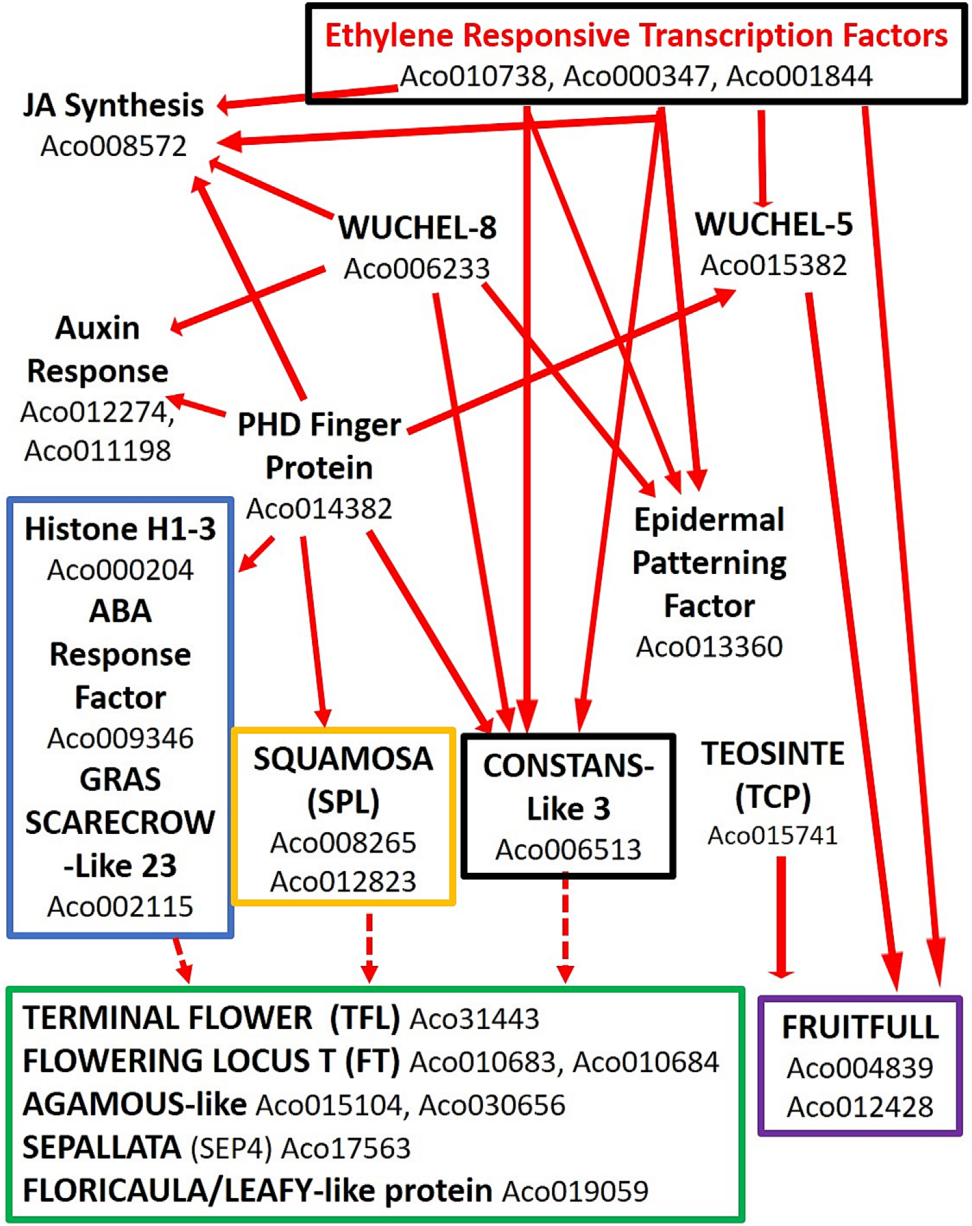
Summary of potential gene network interaction based on differential expression and Trans‐Cis motif targets. Solid arrows indicate potential Trans‐Cis connections, and dashed arrows suggest potential connections. Three ethylene‐responsive transcriptions including Aco001844 (Figure 2i) had Trans‐Cis target involved in JA synthesis (Figure 3a), WUSCHEL (Figure 9), EPIDERMAL PATTERNING FACTOR (Aco013360), and two up‐regulated FRUITFUL genes (Figure 8d,e) that could interact with AcSOC1c (Figure 7c), although not differentially expressed in the early stages and modulate FT (Figure 9a), SPL (Figure 8f), AGAMOUS (Figure 8b), and CO (Figure 7a) leading to floral transition. Auxin response gene (Aco012274, Aco011198), another WUSCHEL gene (Aco006233), and DNA modification seemed to interact with a PHD Zn‐finger protein (Aco014382), histones H1–3, ABA (Aco009346), and a GRAS SCARECROW‐like (Aco002115).

Differential expression patterns and the analysis of target genes for cis‐acting elements revealed tissue‐specific spatiotemporal expression patterns that allowed us to develop a model of potential interactions that led to the conversion of the pineapple apex from a vegetative to a floral state (Figure 12). In this model, the basic assumption was that the response to ethephon treatment was initiated by ethylene response factors. The second assumption was that we were looking for a gene interaction that caused the transition to a floral meristem (floret bract formation) reported to be observable in pineapple in 7 to 10 days (Bartholomew, 1977; Espinosa et al., 2017). Flowers are not seen in this floral meristem for another 5 to 6 weeks (Bartholomew, 1977; Chen et al., 2019; Mao et al., 2018); therefore, genes associated with the identity of floral organs (sepals, petals, stamens, and carpels) (Bowman et al., 1989, 2012; Coen & Meyerowitz, 1991) were not a focus or expected in this study that deals with the transition from vegetative to floral meristem.

The model of the gene expression network (Figure 12) involves ERTF targets (Aco001844), WUSCHEL (Aco015382), and two MADS‐box genes (Aco004839–AcFUL1 [Figure 9d] and Aco012428–AcFUL2 [Figure 9e]). Another target of these two MADS‐box genes that are involved in controlling the timing of flowering and meristem identity (Callens et al., 2018; Hu et al., 2021; Liu et al., 2013; Stewart et al., 2016) is Teosinte (Aco015741), a noncanonical gene of the bHLH TCP domain (Dhaka et al., 2017). Class II are involved in cell division during floral transition and organ development in model systems such as Arabidopsis and maize (Dhaka et al., 2017; Li, 2015). Two additional ERTFs were rapidly up‐regulated after treatment (Aco000347 and Aco010738; Table 1) and have multiple binding targets that can lead to a cascade of responses. The targets of these two up‐regulated ERTF include JA synthase (Aco008572; Figure 4a), epidermal patterning‐like factor (Aco013360), and potentially critical CONSTANS‐like 3 gene (Aco006513). Maternal effect embryo arrest (Aco008567) is down‐regulated by these two ERTFs. Another WUSCHEL Homeobox 8 gene (Aco006233) also has as a target the same CONSTANS gene and, in addition, the same JA synthase gene and the auxin response protein (Aco012374). The next key network player is the PHD Homeobox gene (Aco014382; Figure 7f). PHD homeodomain proteins are involved in plant growth and development (Alam et al., 2019; López‐González et al., 2014; Searle et al., 2006) and are co‐expressed with auxin and ethylene response factors (Müller & Munné‐Bosch, 2015; Roosjen et al., 2017). These proteins have histone binding tails that have been shown to play a role in the regulation of plant growth (Zhao et al., 2018) and flowering time potentially through enhanced binding of BAH transcription regulators to trimethylated histone H3 (Qian et al., 2021). The targets of this PHD homeobox include the same JA synthase, the Auxin response factor, and CONSTANS. Other targets include two SQUAMOSA proteins (Aco008265 [Figure 9f] and Aco012823) and a group of genes for Auxin response factor (Aco011198), Abscisic response binding factor (Aco009346), GRAS SCARECROW‐like (Aco002115), and Histone‐3 (Aco000204). We found no target connection of the Trans‐Cis motif to MADS‐box genes that are up‐regulated soon after treatment: TERMINAL FLOWER (TFL1) (Aco031443), FLOWERING LOCUS T (FT) (Aco010683 [Figure 9a] and Aco010684), two AGAMOUS‐like (Aco015104 and Aco030656; Figure 9c), SOC1c (Aco0152492; Figure 8c), or SEPALLATA (SEP4) (Aco017563). FLC, whose role in the flowering of cereals and eudicots is regulated by vernalization and other environmental controls via the suppression floral integration genes such as FT and SOC1 (Kennedy & Koen, 2020; Madrid et al., 2020). This loss of FLC in pineapple removes a level of flowering control and allows other pathways and control to function. Furthermore, the three corepressor TOPLESS genes (Aco006421, Aco018149, and Aco018150) were not DE and may not play a role in repressing CO and FT (Causier et al., 2011; Goralogia et al., 2017; Graeff et al., 2016) It is possible that FUL, CO‐like‐1, SOC1c, FT, SEP4, and SPL could interact to form a flower activation complex that involves phosphorylation, nuclear localization, and interaction with 14‐3‐3 receptors such as Aco018444, general response factors, and histones to initiate the transition from vegetative to floral meristem.

## 5 CONCLUSIONS

Genes controlling the vegetative to floral meristem transition show considerable conservation. Similar to other systems, including orchids with their highly specialized flower structure (Li et al., 2022; Putterill et al., 2004), gene duplication, and subfunctionalization are common themes. Pineapple appears to have lost its flowering repressor, FLOWERING LOCUS C, and in the initial stages, the SUPPRESSOR OF OVEREXPRESSION OF CONSTANS 1 (SOC1) was not significantly involved in this transition. CONSTANS (CO) was a positive regulator and hub that had cis binding motifs that bind to ETHYLENE RESPONSE (ERTF), WUSCHEL, and PHD homeobox transcription factors. APETALA1/FRUTFULL (AP1/FUL) was also a target for an up‐regulated ERTF. The FLOWERING LOCUS T (FT), TERMINAL FLOWER (TFL), AGAMOUS‐like APETELAR (AP2), and SEPETALA (SEP) increased rapidly within 2–3 days after ethylene treatment. DNA modification and new histone gene expression were a common theme. ERTF had a direct effect on JA synthesis and indirectly on Auxin response genes with suppression of abscisic acid‐responsive binding factor and SQUAMOSA protein expression. This indicated that the ethylene response in pineapple acted directly as a promoter of the vegetative‐to‐flowering transition through APETALA1/FRUITFULL (AP1/FUL). AP1/FUL modifying the cell differentiation of the shoot apical meristem similar to Arabidopsis and tomato (Balanzà et al., 2014; Ferrandiz et al., 2000; Jiang et al., 2021; Litt & Irish, 2003; McCarthy et al., 2015) through TFL, LFY, and CAULIFLOWER (CAL), although a homolog of CAL was not DE in pineapple or reported by others in pineapple (Wang, Li, et al., 2020). The foundational knowledge presented here contributes to our molecular regulatory understanding of ethylene induction of flowering in a non‐model system that is a base group in the Poales. In addition, the results give direction to potential candidate genes that are keys to flowering control in Bromeliads.

## AUTHOR CONTRIBUTIONS

**Robert E. Paull:** Conceptualization, methodology, formal analysis, data curation, resources, writing (Draught & Editing), editing, supervision, funding acquisition. **Najla Ksouri:** Methodology, validation, formal analysis, investigation, data collection, writing (draught, review and editing), visualization. **Michael Kantar:** Methodology, formal analysis, validation, visualization, writing (draft and editing). **Dessireé Zerpa‐Catanho:** Methodology, formal analysis, investigation, writing (review and editing). **Nancy Jung Chen:** Methodology, Validation, Formal Analysis, Investigation, Writing (Review and editing). **Gail Uruu:** Visualization, Writing (Review and editing). **Jingjing Yue:** Sequencing and assembly. **Shiyong Guo:** Assembly and Curation of Small‐RNA. **Yun Zheng:** Small‐RNA analysis and writing. **Ching Man Jennifer Wai:** Investigation, resources, writing (review and editing). **Ray Ming:** Validation, Resources, Writing (Review and editing), supervision, Funding Acquisition.

## CONFLICT OF INTEREST STATEMENT

The authors declare that they have no competing interests.

## PEER REVIEW

The peer review history for this article is available in the Supporting Information for this article.

## Supporting information


**Data S1.** Supplementary Results SR1 DEGs associated with cytokinin synthesis and response, abscisic acid, LATE EMBRYOGENESIS ABUNDANT (LEA) genes, nodulin‐related, stress‐related, sugar metabolism, OVATE, and light‐dependent short hypocotyl, protein turnover and interactions, regulation of transport gradients, sugar metabolism and transporters, and our network and temporal analysis.

**Table S1.** Log2 means and standard error of 19,824 genes expressed following treatment with ethephon in leaf bases and apex of pineapple. CAP1 to CAP8 are water control treatment apex tissue while Cl1 to CL5 are leaf bases expression data. TAP1 to TAP8 and TL1 to TL5 were the apex and leaf base from ethephon treated tissue, respectively. Arabidopsis ortholog ID and BLAST Hit Accession AHRD‐2.0 and Quality Index added from Xu et al., (2018).
**Table S2.** Summary of apex and leaf base gene expression tagged as significantly different (P < .05) using edgeR (Robinson et al., 2010) on OmnicsBox OmicsBox ‐ Bioinformatics made easy. BioBam Bioinformatics (Version 2.1.10) (Nueda et al., 2014). Gene annotation, transcription factor family, whether previously reported as being involved in pineapple flowering and possible miRNA target. Up and down regulation at different sampling stages and statical results and hierarchical cluster group determined. KEGG reference pathway map and function, and InterPro Classification Accession number, annotation and GO were determined for these 7,962 DEGs.
**Table S3.** The transcription binding motif for selected DE genes including ethylene response factors, organism, BLAST e‐value, interface similarity, FootprintDB consensus, and Pfam Domain.
**Table S4.** Transcription factor motifs predicted target genes from a full pineapple genome search the transcription factors Pfam domains, TF Motif, Target pineapple gene Aco#, Target Annotation, Published Name and miR Target 2016 data (Zheng et al., 2016).
**Table S5.** Phylogenetic analysis AP2/ERTF expressed genes. The pineapple genome database was searched for all members of the AP2/ERTF family members. BLASTP at NCBI was used to screen the predicted amino acid sequences. The pineapple differentially expressed AP2/ERTF genes were BLASTP against the Arabidopsis AP2/ERTF PlantTFDB database at http://planttfdb.gao-lab.org/prediction_result.php (Jin et al., 2017) and assigned to specific classes and group using the Arabidopsis and rice classification system of Nakano et al. (2006). The ERTF highlighted in red were predicted to play a critical role in pineapple flowering transition.
**Table S6.** Gene coexpression network was inferred using GWENA (Lemoine et al., 2021) a modified version of WGCNA (Langfelder & Horvath, 2008) that displays coexpression networks, network modules, hub gene detection and differential coexpression. The network was visualized in the R program (R Core Team, 2021) (See supplementary Figure S5) and the weight cutoff was set at a value <.01.
**Table S7.** The small RNA raw count values were normalized by calculating their reads per ten million sequencing tags. The average RPTM of miRs were summarized.
**Table S8.** Significant temporal expression changes and significant differences of small RNA between treated and control determined by ‘maSigPro’ (Bioconductor) in OmicsBox (BioBam Version 2.1.10) (Nueda et al., 2014). Up and down regulation at different sampling stages and statical results and hierarchical cluster group determined.

**Figure S1.** InterPro domain motifs of the top twelve up‐ and down‐regulated differentially expressed genes. The AP2‐ERTF genes in the individual Pie Charts are boxed in a red rectangle. The number in each pie slice are the number of genes that were DE at that sampling. The complete list of InterPro domains and GO differentially expressed are found in Supplementary Table ST2.
**Figure S2.** Pineapple flowering transcriptome library quality. A. Total number of reads, B. Normalized Log_2_ counts per million, and C. Assignment of total reads as percent of total from control and treated apex and leaf bases at different sampling times.
**Figure S3.** Feature map of the predicted DNA‐binding sites. DNA motifs were grouped based on their Pfam domains. The x‐axis corresponds to upstream length [−1,500 bp, +200 bp] around the TSS. The y‐axis corresponds to the density of captured sites with P‐value <10 e−4. Black dots correspond the occurrence of each captured site.
**Figure S4.** Expression patterns of target genes in broad categories with cis‐domains to the transcription factors given in Figure 9 following ethephon treatment in the apex and leaf base.
**Figure S5.** Gene expression network (GWENA) for treated apex at the same first sampling 6 hr after ethylene treatment. Ethylene responsive transcription factors were in all cluster except #4 and #14.

## Data Availability

Data supporting the findings of this study are openly available on Gene Expression Omnibus (GEO http://www.ncbi.nlm.nih.gov/geo/) under accession numbers GSE239489 for mRNA and GSE241286 for small RNA. Transcription data are also available in the Pineapple genome database (Xu et al., 2018) at http://pineapple.angiosperms.org/pineapple/html/index.html.

## References

[pld3541-bib-0001] Abe, M. , Kobayashi, Y. , Yamamoto, S. , Daimon, Y. , Yamaguchi, A. , Ikeda, Y. , Ichinoki, H. , Notaguchi, M. , Goto, K. , & Araki, T. (2005). FD, a bZIP protein mediating signals from the floral pathway integrator FT at the shoot apex. Science, 309, 1052–1056. 16099979 10.1126/science.1115983

[pld3541-bib-0002] Abeles, F. B. , Morgan, P. W. , & Saltveit, M. E. J. (1992). Ethylene in plant biology. Academic Press.

[pld3541-bib-0003] Achard, P. , Baghour, M. , Chapple, A. , Hedden, P. , Van Der Straeten, D. , Genschik, P. , Moritz, T. , & Harberd, N. P. (2007). The plant stress hormone ethylene controls floral transition via DELLA‐dependent regulation of floral meristem‐identity genes. Proceedings of the National Academy of Sciences, 104, 6484–6489. 10.1073/pnas.0610717104 PMC1851083 17389366

[pld3541-bib-0004] Alam, I. , Liu, C.‐C. , Ge, H.‐L. , Batool, K. , Yang, Y.‐Q. , & Lu, Y.‐H. (2019). Genome wide survey, evolution and expression analysis of PHD finger genes reveal their diverse roles during the development and abiotic stress responses in Brassica rapa L. BMC Genomics, 20, 773. 31651238 10.1186/s12864-019-6080-8 PMC6814106

[pld3541-bib-0005] Amasino, R. (2010). Seasonal and developmental timing of flowering. The Plant Journal, 61, 1001–1013. 20409274 10.1111/j.1365-313X.2010.04148.x

[pld3541-bib-0006] An, H. , Roussot, C. , Suárez‐López, P. , Corbesier, L. , Vincent, C. , Piñeiro, M. , Hepworth, S. , Mouradov, A. , Justin, S. , Turnbull, C. , & Coupland, G. (2004). CONSTANS acts in the phloem to regulate a systemic signal that induces photoperiodic flowering of Arabidopsis. Development, 131, 3615–3626. 15229176 10.1242/dev.01231

[pld3541-bib-0007] Aukerman, M. J. , & Sakai, H. (2003). Regulation of flowering time and floral organ identity by a MicroRNA and its APETALA2‐like target genes. The Plant Cell, 15, 2730–2741. 14555699 10.1105/tpc.016238 PMC280575

[pld3541-bib-0008] Balanzà, V. , Martínez‐Fernández, I. , & Ferrándiz, C. (2014). Sequential action of FRUITFULL as a modulator of the activity of the floral regulators SVP and SOC1. Journal of Experimental Botany, 65, 1193–1203. 24465009 10.1093/jxb/ert482 PMC3935574

[pld3541-bib-0009] Balanzà, V. , Martínez‐Fernández, I. , Sato, S. , Yanofsky, M. F. , Kaufmann, K. , Angenent, G. C. , Bemer, M. , & Ferrándiz, C. (2018). Genetic control of meristem arrest and life span in Arabidopsis by a FRUITFULL‐APETALA2 pathway. Nature Communications, 9, 565. 10.1038/s41467-018-03067-5 PMC5805735 29422669

[pld3541-bib-0010] Bao, S. , Hua, C. , Shen, L. , & Yu, H. (2020). New insights into gibberellin signaling in regulating flowering in Arabidopsis. Journal of Integrative Plant Biology, 62, 118–131. 31785071 10.1111/jipb.12892

[pld3541-bib-0011] Bartholomew, D. P. (1977). Inflorescence development of pineapple (*Ananas comosus* [L.] Merr.) induced to flower with Ethephon. Botanical Gazette, 138, 312–320.

[pld3541-bib-0012] Bartholomew, D. P. (2014). History and perspectives on the role of ethylene in pineapple flowering. Acta Horticulturae, 1042, 269–284.

[pld3541-bib-0013] Bartholomew, D. P. , Malézieux, E. , Sanewsk, G. M. , & Sinclair, E. (2003). Inflorescence and fruit development and yield. In D. P. Bartholomew , R. E. Paull , & K. G. Rohrbach (Eds.), The pineapple: Botany, production and uses (pp. 167–202). CABI International. 10.1079/9780851995038.0167

[pld3541-bib-0014] Bartholomew, D. P. , & Sanewski, G. M. (2018). Inflorescence and fruit development and yield. In G. M. Sanewski , D. P. Bartholomew , & R. E. Paull (Eds.), The pineapple: Botany, production and uses, CABI (2nd ed., pp. 233–268). Wallingford, United Kingdom: CAB International.

[pld3541-bib-0015] Barton, M. K. (2010). Twenty years on: The inner workings of the shoot apical meristem, a developmental dynamo. Developmental Biology, 341, 95–113. 19961843 10.1016/j.ydbio.2009.11.029

[pld3541-bib-0016] Baucher, M. , Moussawi, J. , Vandeputte, O. M. , Monteyne, D. , Mol, A. , Pérez‐Morga, D. , & El Jaziri, M. (2013). A role for the miR396/GRF network in specification of organ type during flower development, as supported by ectopic expression of Populus trichocarpa miR396c in transgenic tobacco. Plant Biology, 15, 892–898. 23173976 10.1111/j.1438-8677.2012.00696.x

[pld3541-bib-0017] Becker, A. , & Theißen, G. (2003). The major clades of MADS‐box genes and their role in the development and evolution of flowering plants. Molecular Phylogenetics and Evolution, 29, 464–489. 14615187 10.1016/s1055-7903(03)00207-0

[pld3541-bib-0018] Becraft, P. W. (2002). Receptor kinase signaling in plant development. Annual Review of Cell and Developmental Biology, 18, 163–192. 10.1146/annurev.cellbio.18.012502.083431 12142267

[pld3541-bib-0019] Beltramino, M. , Debernardi, J. M. , Ferela, A. , & Palatnik, J. F. (2021). ARF2 represses expression of plant GRF transcription factors in a complementary mechanism to microRNA miR396. Plant Physiology, 185, 1798–1812. 33580700 10.1093/plphys/kiab014 PMC8133599

[pld3541-bib-0020] Berr, A. , Ménard, R. , Heitz, T. , & Shen, W.‐H. (2012). Chromatin modification and remodelling: A regulatory landscape for the control of Arabidopsis defence responses upon pathogen attack. Cellular Microbiology, 14, 829–839. 22405188 10.1111/j.1462-5822.2012.01785.x

[pld3541-bib-0021] Binder, B. M. , O'Malley, R. C. , Wang, W. , Moore, J. M. , Parks, B. M. , Spalding, E. P. , & Bleecker, A. B. (2004). Arabidopsis seedling growth response and recovery to ethylene. A Kinetic Analysis. Plant Physiology, 136, 2913–2920. 15466220 10.1104/pp.104.050369 PMC523353

[pld3541-bib-0022] Binder, B. M. , O'Malley, R. C. , Wang, W. , Zutz, T. C. , & Bleecker, A. B. (2006). Ethylene stimulates nutations that are dependent on the ETR1 receptor. Plant Physiology, 142, 1690–1700. 17071649 10.1104/pp.106.087858 PMC1676061

[pld3541-bib-0023] Bolger, A. M. , Lohse, M. , & Usadel, B. (2014). Trimmomatic: A flexible trimmer for Illumina sequence data. Bioinformatics, 30, 2114–2120. 24695404 10.1093/bioinformatics/btu170 PMC4103590

[pld3541-bib-0024] Borg, M. , Jiang, D. , & Berger, F. (2021). Histone variants take center stage in shaping the epigenome. Current Opinion in Plant Biology, 61, 101991. 33434757 10.1016/j.pbi.2020.101991

[pld3541-bib-0025] Boss, P. K. , Bastow, R. M. , Mylne, J. S. , & Dean, C. (2004). Multiple pathways in the decision to flower: Enabling, promoting, and resetting. The Plant Cell, 16, S18–S31. 10.1105/tpc.015958 15037730 PMC2643402

[pld3541-bib-0026] Botella, J. R. , Cavallaro, A. S. , & Cazzonelli, C. I. (2000). Towards the production of transgenic pineapple to control flowering and ripening. Acta Horticulturae, 529, 115–122.

[pld3541-bib-0027] Bouchenak‐Khellardi, Y. , Muthama, M. A. , & Linder, H. P. (2014). A revised evolutionary history of Poales: Origins and diversification. Botanical Journal of the Linnean Society, 175, 4–16.

[pld3541-bib-0028] Bowman, J. L. , Smyth, D. R. , & Meyerowitz, E. M. (1989). Genes directing flower development in Arabidopsis. The Plant Cell, 1, 37–52. 2535466 10.1105/tpc.1.1.37 PMC159735

[pld3541-bib-0029] Bowman, J. L. , Smyth, D. R. , & Meyerowitz, E. M. (2012). The ABC model of flower development: Then and now. Development, 139, 4095–4098. 23093420 10.1242/dev.083972

[pld3541-bib-0030] Boycheva, I. , Vassileva, V. , & Iantcheva, A. (2014). Histone acetyltransferases in plant development and plasticity. Current Genomics, 15, 28–37. 24653661 10.2174/138920291501140306112742 PMC3958957

[pld3541-bib-0031] Burg, S. P. , & Burg, E. A. (1966). Auxin‐induced ethylene formation: Its relation to flowering in the pineapple. Science, 152, 1269–1269. 5937118 10.1126/science.152.3726.1269

[pld3541-bib-0032] Callens, C. , Tucker, M. R. , Zhang, D. , & Wilson, Z. A. (2018). Dissecting the role of MADS‐box genes in monocot floral development and diversity. Journal of Experimental Botany, 69, 2435–2459. 29718461 10.1093/jxb/ery086

[pld3541-bib-0033] Causier, B. , Ashworth, M. , Guo, W. , & Davies, B. (2011). The TOPLESS interactome: A framework for gene repression in Arabidopsis. Plant Physiology, 158, 423–438. 22065421 10.1104/pp.111.186999 PMC3252085

[pld3541-bib-0034] Chang, C. , & Stadler, R. (2001). Ethylene hormone receptor action in Arabidopsis. BioEssays, 23, 619–627. 11462215 10.1002/bies.1087

[pld3541-bib-0035] Chen, H. , Hu, B. , Zhao, L. , Shi, D. , She, Z. , Huang, X. , Priyadarshani, S. V. G. N. , Niu, X. , & Qin, Y. (2019). Differential expression analysis of reference genes in pineapple (*Ananas comosus* L.) during reproductive development and response to abiotic stress, hormonal stimuli. Tropical Plant Biology, 12, 67–77.

[pld3541-bib-0036] Chen, X. , Zhang, Z. , Liu, D. , Zhang, K. , Li, A. , & Mao, L. (2010). SQUAMOSA promoter‐binding protein‐like transcription factors: Star players for plant growth and development. Journal of Integrative Plant Biology, 52, 946–951. 20977652 10.1111/j.1744-7909.2010.00987.x

[pld3541-bib-0037] Chen, Y.‐F. , Etheridge, N. , & Schaller, G. E. (2005). Ethylene signal transduction. Annals of Botany, 95, 901–915. 15753119 10.1093/aob/mci100 PMC4246747

[pld3541-bib-0039] Chongloi, G. L. , Prakash, S. , & Vijayraghavan, U. (2019). Regulation of meristem maintenance and organ identity during rice reproductive development. Journal of Experimental Botany, 70, 1719–1736. 30753578 10.1093/jxb/erz046

[pld3541-bib-0040] Christ, A. , Maegele, I. , Ha, N. , Nguyen, H. H. , Crespi, M. D. , & Maizel, A. (2013). In silico identification and in vivo validation of a set of evolutionary conserved plant root‐specific cis‐regulatory elements. Mechanisms of Development, 130, 70–81. 22504372 10.1016/j.mod.2012.03.002

[pld3541-bib-0042] Coen, E. S. , & Meyerowitz, E. M. (1991). The war of the whorls: Genetic interactions controlling flower development. Nature, 353, 31–37. 1715520 10.1038/353031a0

[pld3541-bib-0043] Corbesier, L. , Vincent, C. , Jang, S. , Fornara, F. , Fan, Q. , Searle, I. , Giakountis, A. , Farrona, S. , Gissot, L. , Turnbull, C. , & Coupland, G. (2007). FT protein movement contributes to long‐distance signaling in floral induction of *Arabidopsis* . Science, 316, 1030–1033. 17446353 10.1126/science.1141752

[pld3541-bib-0045] Cui, X. , Gao, K. , Wang, L. , Lv, M. , Li, Z. , Zheng, D. , Wu, W. , Yao, W. , Ding, L. , Li, X. , Zhu, J.‐K. , & Zhang, H. (2021). General control non‐derepressible 1 (AtGCN1) is important for flowering time, plant growth, seed development, and the transcription/translation of specific genes in Arabidopsis. Frontiers in Plant Science, 12, 630311. 33868334 10.3389/fpls.2021.630311 PMC8045761

[pld3541-bib-0046] Danilevskaya, O. N. , Meng, X. , & Ananiev, E. V. (2010). Concerted modification of flowering time and inflorescence architecture by ectopic expression of TFL1‐like genes in maize. Plant Physiology, 153, 238–251. 20200067 10.1104/pp.110.154211 PMC2862429

[pld3541-bib-0047] Debernardi, J. M. , Greenwood, J. R. , Jean Finnegan, E. , Jernstedt, J. , & Dubcovsky, J. (2020). APETALA 2‐like genes AP2L2 and Q specify lemma identity and axillary floral meristem development in wheat. The Plant Journal, 101, 171–187. 31494998 10.1111/tpj.14528 PMC6972666

[pld3541-bib-0048] Dhaka, N. , Bhardwaj, V. , Sharma, M. K. , & Sharma, R. (2017). Evolving tale of TCPs: New paradigms and old lacunae. Frontiers in Plant Science, 8, 479. 10.3389/fpls.2017.00479 28421104 PMC5376618

[pld3541-bib-0049] Espinosa, M. E. Á. , Moreira, R. O. , Lima, A. A. , Ságio, S. A. , Barreto, H. G. , Luiz, S. L. P. , Abreu, C. E. A. , Yanes‐Paz, E. , Ruíz, Y. C. , González‐Olmedo, J. L. , & Chalfun‐Júnior, A. (2017). Early histological, hormonal, and molecular changes during pineapple (A*nanas comosus* [L.] Merrill) artificial flowering induction. Journal of Plant Physiology, 209, 11–19. 27988471 10.1016/j.jplph.2016.11.009

[pld3541-bib-0050] Ferrandiz, C. , Gu, Q. , Martienssen, R. , & Yanofsky, M. F. (2000). Redundant regulation of meristem identity and plant architecture by FRUITFULL, APETALA1 and CAULIFLOWER. Development, 127, 725–734. 10648231 10.1242/dev.127.4.725

[pld3541-bib-0052] Fletcher, J. C. , Brand, U. , Running, M. P. , Simon, R. , & Meyerowitz, E. M. (1999). Signaling of cell fate decisions by *CLAVATA3* in *Arabidopsis* shoot meristems. Science, 283, 1911–1914. 10082464 10.1126/science.283.5409.1911

[pld3541-bib-0053] Fonini, L. S. , Lazzarotto, F. , Barros, P. M. , Cabreira‐Cagliari, C. , Martins, M. A. B. , Saibo, N. J. , Turchetto‐Zolet, A. C. , & Margis‐Pinheiro, M. (2020). Molecular evolution and diversification of the GRF transcription factor family. Genetics and Molecular Biology, 43, 280–283. 10.1590/1678-4685-GMB-2020-0080 PMC7380329 32706846

[pld3541-bib-0054] Friend, D. J. C. , & Lydon, J. (1979). Effects of daylength on flowering, growth, and CAM of pineapple (*Ananas comosus* [L.] Merrill). Botanical Gazette, 140, 280–283.

[pld3541-bib-0055] Fu, J. , Yang, L. , & Dai, S. (2015). Identification and characterization of the CONSTANS‐like gene family in the short‐day plant Chrysanthemum lavandulifolium. Molecular Genetics and Genomics, 290, 1039–1054. 25523304 10.1007/s00438-014-0977-3

[pld3541-bib-0056] Fu, W. , Huang, S. , Gao, Y. , Zhang, M. , Qu, G. , Wang, N. , Liu, Z. , & Feng, H. (2020). Role of BrSDG8 on bolting in Chinese cabbage (*Brassica rapa*). Theoretical and Applied Genetics, 133, 2937–2948. 32656681 10.1007/s00122-020-03647-4

[pld3541-bib-0057] Galli, M. , Feng, F. , & Gallavotti, A. (2020). Mapping regulatory determinants in plants. Frontiers in Genetics, 11, 591194. 33193733 10.3389/fgene.2020.591194 PMC7655918

[pld3541-bib-0058] Goralogia, G. S. , Liu, T.‐K. , Zhao, L. , Panipinto, P. M. , Groover, E. D. , Bains, Y. S. , & Imaizumi, T. (2017). CYCLING DOF FACTOR 1 represses transcription through the TOPLESS co‐repressor to control photoperiodic flowering in Arabidopsis. The Plant Journal, 92, 244–262. 28752516 10.1111/tpj.13649 PMC5634919

[pld3541-bib-0060] Gowing, D. P. (1961). Experiments on the photoperiodic response in pineapple. American Journal of Botany, 48, 16–21.

[pld3541-bib-0061] Graeff, M. , Straub, D. , Eguen, T. , Dolde, U. , Rodrigues, V. , Brandt, R. , & Wenkel, S. (2016). MicroProtein‐mediated recruitment of CONSTANS into a TOPLESS trimeric complex represses flowering in Arabidopsis. PLoS Genetics, 12, e1005959. 27015278 10.1371/journal.pgen.1005959 PMC4807768

[pld3541-bib-0062] Gregis, V. , Andrés, F. , Sessa, A. , Guerra, R. F. , Simonini, S. , Mateos, J. L. , Torti, S. , Zambelli, F. , Prazzoli, G. M. , Bjerkan, K. N. , Grini, P. E. , Pavesi, G. , Colombo, L. , Coupland, G. , & Kater, M. M. (2013). Identification of pathways directly regulated by SHORT VEGETATIVE PHASE during vegetative and reproductive development in Arabidopsis. Genome Biology, 14, R56. 23759218 10.1186/gb-2013-14-6-r56 PMC3706845

[pld3541-bib-0063] Griffiths, S. , Dunford, R. P. , Coupland, G. , & Laurie, D. A. (2003). The evolution of CONSTANS‐like gene families in barley, rice, and Arabidopsis. Plant Physiology, 131, 1855–1867. 12692345 10.1104/pp.102.016188 PMC166942

[pld3541-bib-0064] Guo, A.‐Y. , Zhu, Q.‐H. , Gu, X. , Ge, S. , Yang, J. , & Luo, J. (2008). Genome‐wide identification and evolutionary analysis of the plant specific SBP‐box transcription factor family. Gene, 418, 1–8. 18495384 10.1016/j.gene.2008.03.016

[pld3541-bib-0065] Hall, A. E. , & Bleecker, A. B. (2003). Analysis of combinatorial loss‐of‐function mutants in the Arabidopsis ethylene receptors reveals that the ers1 etr1 double mutant has severe developmental defects that are EIN2 dependent. The Plant Cell, 15, 2032–2041. 12953109 10.1105/tpc.013060 PMC181329

[pld3541-bib-0066] Hay, A. , & Tsiantis, M. (2010). KNOX genes: Versatile regulators of plant development and diversity. Development, 137, 3153–3165. 20823061 10.1242/dev.030049

[pld3541-bib-0067] Hecht, V. , Foucher, F. , Ferrándiz, C. , Macknight, R. , Navarro, C. , Morin, J. , Vardy, M. E. , Ellis, N. , Beltrán, J. P. , Rameau, C. , & Weller, J. L. (2005). Conservation of Arabidopsis flowering genes in model legumes. Plant Physiology, 137, 1420–1434. 15778459 10.1104/pp.104.057018 PMC1088331

[pld3541-bib-0069] Hnatuszko‐Konka, K. , Gerszberg, A. , Weremczuk‐Jeżyna, I. , & Grzegorczyk‐Karolak, I. (2021). Cytokinin signaling and de novo shoot organogenesis. Genes, 12, 265. 33673064 10.3390/genes12020265 PMC7917986

[pld3541-bib-0070] Hu, J. , Chang, X. , Zhang, Y. , Yu, X. , Qin, Y. , Sun, Y. , & Zhang, L. (2021). The pineapple MADS‐box gene family and the evolution of early monocot flower. Scientific Reports, 11, 849. 33441609 10.1038/s41598-020-79163-8 PMC7806820

[pld3541-bib-0071] Hua, J. , Chang, C. , Sun, Q. , & Meyerowitz, E. M. (1995). Ethylene insensitivity conferred by *Arabidopsis* ERS gene. Science, 269, 1712–1714. 7569898 10.1126/science.7569898

[pld3541-bib-0072] Hua, J. , Sakai, H. , Nourizadeh, S. , Chen, Q. G. , Bleecker, A. B. , Ecker, J. R. , & Meyerowitz, E. M. (1998). EIN4 and ERS2 are members of the putative ethylene receptor gene family in Arabidopsis. The Plant Cell, 10, 1321–1332. 9707532 10.1105/tpc.10.8.1321 PMC144061

[pld3541-bib-0073] Huang, H. , Chen, Y. , Wang, S. , Qi, T. , & Song, S. (2022). Jasmonate action and crosstalk in flower development and fertility. Journal of Experimental Botany, 74, 1186–1197. 10.1093/jxb/erac251 35670512

[pld3541-bib-0074] Inukai, S. , Kock, K. H. , & Bulyk, M. L. (2017). Transcription factor–DNA binding: Beyond binding site motifs. Current Opinion in Genetics & Development, 43, 110–119. 28359978 10.1016/j.gde.2017.02.007 PMC5447501

[pld3541-bib-0075] Ishimori, M. (2022). Transcription factor binding site prediction: Finding the point from many data. Plant and Cell Physiology, 63, 1324–1325. 36073809 10.1093/pcp/pcac128

[pld3541-bib-0076] Jaeger, K. E. , Pullen, N. , Lamzin, S. , Morris, R. J. , & Wigge, P. A. (2013). Interlocking feedback loops govern the dynamic behavior of the floral transition in Arabidopsis. The Plant Cell, 25, 820–833. 23543784 10.1105/tpc.113.109355 PMC3634691

[pld3541-bib-0077] Jarillo, J. A. , & Piñeiro, M. (2015). H2A.Z mediates different aspects of chromatin function and modulates flowering responses in Arabidopsis. The Plant Journal, 83, 96–109. 25943140 10.1111/tpj.12873

[pld3541-bib-0078] Jiang, X. , Lubini, G. , Hernandes‐Lopes, J. , Rijnsburger, K. , Veltkamp, V. , de Maagd, R. A. , Angenent, G. C. , & Bemer, M. (2021). FRUITFULL‐like genes regulate flowering time and inflorescence architecture in tomato. The Plant Cell, 34, 1002–1019. 10.1093/plcell/koab298 PMC8894982 34893888

[pld3541-bib-0079] Jin, J. P. , Tian, F. , Yang, D. C. , Meng, Y. Q. , Kong, L. , Luo, J. C. , & Gao, G. (2017). PlantTFDB 4.0: Toward a central hub for transcription factors and regulatory interactions in plants. Nucleic Acids Research, 45(D1), D1040–D1045. 27924042 10.1093/nar/gkw982 PMC5210657

[pld3541-bib-0080] Jin, M. , Liu, X. , Jia, W. , Liu, H. , Li, W. , Peng, Y. , Du, Y. , Wang, Y. , Yin, Y. , Zhang, X. , Liu, Q. , Deng, M. , Li, N. , Cui, X. , Hao, D. , & Yan, J. (2018). ZmCOL3, a CCT gene represses flowering in maize by interfering with the circadian clock and activating expression of ZmCCT. Journal of Integrative Plant Biology, 60, 465–480. 29319223 10.1111/jipb.12632

[pld3541-bib-0081] Jones, D. M. , & Vandepoele, K. (2020). Identification and evolution of gene regulatory networks: Insights from comparative studies in plants. Current Opinion in Plant Biology, 54, 42–48. 32062128 10.1016/j.pbi.2019.12.008

[pld3541-bib-0083] Kendrick, M. D. , & Chang, C. (2008). Ethylene signaling: New levels of complexity and regulation. Current Opinion in Plant Biology, 11, 479–485. 18692429 10.1016/j.pbi.2008.06.011 PMC2562597

[pld3541-bib-0084] Kennedy, A. , & Koen, G. (2020). The role of FLOWERING LOCUS C relatives in cereals. Frontiers in Plant Science, 11, 617340. 10.3389/fpls.2020.617340 33414801 PMC7783157

[pld3541-bib-0085] Kevany, B. M. , Tieman, D. M. , Taylor, M. G. , Cin, V. D. , & Klee, H. J. (2007). Ethylene receptor degradation controls the timing of ripening in tomato fruit. The Plant Journal, 51, 458–467. 17655616 10.1111/j.1365-313X.2007.03170.x

[pld3541-bib-0086] Kim, D. Y. , Hong, M. J. , & Seo, Y. W. (2019). Genome‐wide transcript analysis of inflorescence development in wheat. Genome, 62, 623–633. 31269405 10.1139/gen-2018-0200

[pld3541-bib-0087] Kim, J. H. , & Tsukaya, H. (2015). Regulation of plant growth and development by the GROWTH‐REGULATING FACTOR and GRF‐INTERACTING FACTOR duo. Journal of Experimental Botany, 66, 6093–6107. 26160584 10.1093/jxb/erv349

[pld3541-bib-0088] Kinoshita, A. , & Richter, R. (2020). Genetic and molecular basis of floral induction in Arabidopsis thaliana. Journal of Experimental Botany, 71, 2490–2504. 32067033 10.1093/jxb/eraa057 PMC7210760

[pld3541-bib-0089] Kitagawa, M. , & Jackson, D. (2019). Control of meristem size. Annual Review of Plant Biology, 70, 269–291. 10.1146/annurev-arplant-042817-040549 31035828

[pld3541-bib-0237] Klein, J. , Saedler, H. , & Huijser, P. (1996). A new family of DNA binding proteins includes putative transcriptional regulators of the Antirrhinum majus floral meristem identity gene SQUAMOSA. Molecular and General Genetics MGG, 250(1), 7–16. 10.1007/bf02191820 8569690

[pld3541-bib-0090] Koschmann, J. , Machens, F. , Becker, M. , Niemeyer, J. , Schulze, J. , Bülow, L. , Stahl, D. J. , & Hehl, R. (2012). Integration of bioinformatics and synthetic promoters leads to the discovery of novel elicitor‐responsive cis‐regulatory sequences in Arabidopsis. Plant Physiology, 160, 178–191. 22744985 10.1104/pp.112.198259 PMC3440196

[pld3541-bib-0091] Kozomara, A. , Birgaoanu, M. , & Griffiths‐Jones, S. (2018). miRBase: From microRNA sequences to function. Nucleic Acids Research, 47, D155–D162. 10.1093/nar/gky1141 PMC6323917 30423142

[pld3541-bib-0092] Ksouri, N. , Castro‐Mondragón, J. A. , Montardit‐Tarda, F. , van Helden, J. , Contreras‐Moreira, B. , & Gogorcena, Y. (2021). Tuning promoter boundaries improves regulatory motif discovery in nonmodel plants: The peach example. Plant Physiology, 185, 1242–1258. 33744946 10.1093/plphys/kiaa091 PMC8133646

[pld3541-bib-0093] Kurepa, J. , Shull, T. E. , & Smalle, J. A. (2019). Antagonistic activity of auxin and cytokinin in shoot and root organs. Plant Direct, 3, e00121. 31245764 10.1002/pld3.121 PMC6508789

[pld3541-bib-0094] Langfelder, P. , & Horvath, S. (2008). WGCNA: An R package for weighted correlation network analysis. BMC Bioinformatics, 9, 559. 19114008 10.1186/1471-2105-9-559 PMC2631488

[pld3541-bib-0095] Lee, I. , Wolfe, D. S. , Nilsson, O. , & Weigel, D. (1997). A *LEAFY* co‐regulator encoded by *UNUSUAL FLORAL ORGANS* . Current Biology, 7, 95–104. 9016705 10.1016/s0960-9822(06)00053-4

[pld3541-bib-0096] Lee, J. , & Lee, I. (2010). Regulation and function of SOC1, a flowering pathway integrator. Journal of Experimental Botany, 61, 2247–2254. 20413527 10.1093/jxb/erq098

[pld3541-bib-0098] Lei, B. , & Berger, F. (2020). H2A variants in Arabidopsis: Versatile regulators of genome activity. Plant Communications, 1, 100015. 33404536 10.1016/j.xplc.2019.100015 PMC7747964

[pld3541-bib-0099] Lemoine, G. G. , Scott‐Boyer, M.‐P. , Ambroise, B. , Périn, O. , & Droit, A. (2021). GWENA: Gene co‐expression networks analysis and extended modules characterization in a single Bioconductor package. BMC Bioinformatics, 22, 267. 34034647 10.1186/s12859-021-04179-4 PMC8152313

[pld3541-bib-0100] Li, S. (2015). The *Arabidopsis thaliana* TCP transcription factors: A broadening horizon beyond development. Plant Signaling & Behavior, 10, e1044192. 26039357 10.1080/15592324.2015.1044192 PMC4622585

[pld3541-bib-0101] Li, Y. , Zhang, B. , & Yu, H. (2022). Molecular genetic insights into orchid reproductive development. Journal of Experimental Botany, 73, 1841–1852. 10.1093/jxb/erac016 35104310

[pld3541-bib-0102] Li, Y.‐H. , Wu, Q.‐S. , Huang, X. , Liu, S.‐H. , Zhang, H.‐N. , Zhang, Z. , & Sun, G.‐M. (2016). Molecular cloning and characterization of four genes encoding ethylene receptors associated with pineapple (*Ananas comosus* L.) flowering. Frontiers in Plant Science, 7, 1791. 10.3389/fpls.2016.00710 27252725 PMC4878293

[pld3541-bib-0103] Li, Z. , Wang, J. , Zhang, X. , Lei, M. , Fu, Y. , Zhang, J. , Wang, Z. , & Xu, L. (2016). Transcriptome sequencing determined flowering pathway genes in Aechmea fasciata treated with ethylene. Journal of Plant Growth Regulation, 35, 316–329.

[pld3541-bib-0104] Lin, C. H. , Maruthasalam, S. , Shiu, L. Y. , Lien, W. C. , Loganathan, M. , Yu, C. W. , Hung, S. H. , Ko, Y. , & Chen, Y. Y. (2009). Physical and chemical manipulation of flowering in pineapple. Acta Horticulturae, 822, 117–124.

[pld3541-bib-0105] Lin, Z. , Zhong, S. , & Grierson, D. (2009). Recent advances in ethylene research. Journal of Experimental Botany, 60, 3311–3336. 19567479 10.1093/jxb/erp204

[pld3541-bib-0106] Lippman, Z. B. , Cohen, O. , Alvarez, J. P. , Abu‐Abied, M. , Pekker, I. , Paran, I. , Eshed, Y. , & Zamir, D. (2008). The making of a compound inflorescence in tomato and related nightshades. PLoS Biology, 6, e288. 19018664 10.1371/journal.pbio.0060288 PMC2586368

[pld3541-bib-0107] Litt, A. , & Irish, V. F. (2003). Duplication and diversification in the APETALA1/FRUITFULL floral homeotic gene lineage: Implications for the evolution of floral development. Genetics, 165, 821–833. 14573491 10.1093/genetics/165.2.821 PMC1462802

[pld3541-bib-0108] Liu, C. , Teo Zhi Wei, N. , Bi, Y. , Song, S. , Xi, W. , Yang, X. , Yin, Z. , & Yu, H. (2013). A conserved genetic pathway determines inflorescence architecture in *Arabidopsis* and rice. Developmental Cell, 24, 612–622. 10.1016/j.devcel.2013.02.013 23537632

[pld3541-bib-0109] Liu, C.‐H. , & Fan, C. (2016). De novo transcriptome assembly of floral buds of pineapple and identification of differentially expressed genes in response to ethephon induction. Frontiers in Plant Science, 7, 203. 10.3389/fpls.2016.00203 26955375 PMC4767906

[pld3541-bib-0110] Liu, C. H. , Liu, Y. , Shao, X. H. , & Lai, D. (2018). Comparative analyses of the transcriptome and proteome of Comte de Paris and Smooth Cayenne to improve the understanding of ethephon‐induced floral transition in pineapple. Cellular Physiology and Biochemistry, 50, 2139–2156. 30415248 10.1159/000495057

[pld3541-bib-0111] Liu, H. , Guo, S. , Xu, Y. , Li, C. , Zhang, Z. , Zhang, D. , Xu, S. , Zhang, C. , & Chong, K. (2014). OsmiR396d‐regulated OsGRFs function in floral organogenesis in rice through binding to their targets OsJMJ706 and OsCR4. Plant Physiology, 165, 160–174. 24596329 10.1104/pp.114.235564 PMC4012577

[pld3541-bib-0113] Liu, L. , Wu, Y. , Liao, Z. , Xiong, J. , Wu, F. , Xu, J. , Lan, H. , Tang, Q. , Zhou, S. , Liu, Y. , & Lu, Y. (2018). Evolutionary conservation and functional divergence of the LFK gene family play important roles in the photoperiodic flowering pathway of land plants. Heredity, 120, 310–328. 29225355 10.1038/s41437-017-0006-5 PMC5842218

[pld3541-bib-0114] Liu, M. , Wu, Q.‐S. , Liu, S.‐H. , Zhang, H.‐N. , Lin, W.‐Q. , Zhang, X.‐M. , & Li, Y.‐H. (2021). Combining single‐molecule sequencing and Illumina RNA sequencing to elucidate flowering induction of pineapple (*Ananas comosus* [L.] Merr.) treated with exogenous ethylene. Plant Growth Regulation, 94, 303–321.

[pld3541-bib-0115] Liu, Q. , Xu, C. , & Wen, C.‐K. (2010). Genetic and transformation studies reveal negative regulation of ERS1 ethylene receptor signaling in Arabidopsis. BMC Plant Biology, 10, 60. 20374664 10.1186/1471-2229-10-60 PMC2923534

[pld3541-bib-0116] Liu, S.‐h. , Zang, X.‐p. , & Sun, G.‐m. (2011). Changes in endogenous hormone concentrations during inflorescence induction and development in pineapple (*Ananas comosus* cv. Smooth Cayenne) by ethephon. African Journal of Biotechnology, 10, 10892–10899.

[pld3541-bib-0117] Long, J. A. , Moan, E. I. , Medford, J. I. , & Barton, M. K. (1996). A member of the KNOTTED class of homeodomain proteins encoded by the STM gene of Arabidopsis. Nature, 379, 66–69. 8538741 10.1038/379066a0

[pld3541-bib-0118] López‐González, L. , Mouriz, A. , Narro‐Diego, L. , Bustos, R. , Martínez‐Zapater, J. M. , Jarillo, J. A. , & Piñeiro, M. (2014). Chromatin‐dependent repression of the Arabidopsis floral integrator genes involves plant specific PHD‐containing proteins. The Plant Cell, 26, 3922–3938. 25281686 10.1105/tpc.114.130781 PMC4247585

[pld3541-bib-0119] Lv, L. , Duan, J. , Xie, J. , Wei, C. , Liu, Y. , Liu, S. , & Sun, G. (2012). Isolation and characterization of a FLOWERING LOCUS T homolog from pineapple (Ananas comosus [L.] Merr). Gene, 505, 368–373. 10.1016/j.gene.2012.06.011 22710136

[pld3541-bib-0120] Lv, L.‐L. , Duan, J. , Xie, J.‐H. , Liu, Y.‐G. , Wei, C.‐B. , Liu, S.‐H. , Zhang, J.‐X. , & Sun, G.‐M. (2012). Cloning and expression analysis of a PISTILLATA homologous gene from pineapple (Ananas comosus L. Merr). International Journal of Molecular Sciences, 13, 1039–1053. 22312303 10.3390/ijms13011039 PMC3269737

[pld3541-bib-0121] Ma, B. , Chen, H. , Chen, S.‐Y. , & Zhang, J.‐S. (2014). Roles of ethylene in plant growth and responses to stresses. In L.‐S. P. Tran & S. Pal (Eds.), Phytohormones: A window to metabolism, signaling and biotechnological applications (pp. 81–118). New York, NY.

[pld3541-bib-0122] Madrid, E. , Chandler, J. W. , & Coupland, G. (2020). Gene regulatory networks controlled by FLOWERING LOCUS C that confer variation in seasonal flowering and life history. Journal of Experimental Botany, 72, 4–14. 10.1093/jxb/eraa216 PMC7816851 32369593

[pld3541-bib-0123] Mao, Q. , Chen, C. , Xie, T. , Luan, A. , Liu, C. , & He, Y. (2018). Comprehensive tissue‐specific transcriptome profiling of pineapple (*Ananas comosus*) and building an eFP‐browser for further study. PeerJ, 6, e6028. 30564517 10.7717/peerj.6028 PMC6284516

[pld3541-bib-0124] Mathieu, J. , Yant, L. J. , Mürdter, F. , Küttner, F. , & Schmid, M. (2009). Repression of flowering by the miR172 target SMZ. PLoS Biology, 7, e1000148. 10.1371/journal.pbio.1000148 19582143 PMC2701598

[pld3541-bib-0125] Matsoukas, I. G. (2015). Florigens and antiflorigens: A molecular genetic understanding. Essays in Biochemistry, 58, 133–149. 26374892 10.1042/bse0580133

[pld3541-bib-0126] Mayer, K. F. X. , Schoof, H. , Haecker, A. , Lenhard, M. , Jürgens, G. , & Laux, T. (1998). Role of *WUSCHELi*n regulating stem cell fate in the *Arabidopsis* shoot meristem. Cell, 95, 805–815. 9865698 10.1016/s0092-8674(00)81703-1

[pld3541-bib-0127] McCarthy, E. W. , Mohamed, A. , & Litt, A. (2015). Functional divergence of APETALA1 and FRUITFULL is due to changes in both regulation and coding sequence. Frontiers in Plant Science, 6, 1076. 10.3389/fpls.2015.01076 26697035 PMC4667048

[pld3541-bib-0128] Melzer, S. , Lens, F. , Gennen, J. , Vanneste, S. , Rohde, A. , & Beeckman, T. (2008). Flowering‐time genes modulate meristem determinacy and growth form in Arabidopsis thaliana. Nature Genetics, 40, 1489–1492. 18997783 10.1038/ng.253

[pld3541-bib-0129] Merelo, P. , González‐Cuadra, I. , & Ferrándiz, C. (2022). A cellular analysis of meristem activity at the end of flowering points to cytokinin as a major regulator of proliferative arrest in *Arabidopsis* . Current Biology, 32, 749–762.e743. 34963064 10.1016/j.cub.2021.11.069

[pld3541-bib-0130] Meyerowitz, E. M. (1997). Genetic control of cell division patterns in developing plants. Cell, 88, 299–308. 9039256 10.1016/s0092-8674(00)81868-1

[pld3541-bib-0131] Michaels, S. D. , & Amasino, R. M. (1999). FLOWERING LOCUS C encodes a novel MADS domain protein that acts as a repressor of flowering. The Plant Cell, 11, 949–956. 10330478 10.1105/tpc.11.5.949 PMC144226

[pld3541-bib-0132] Ming, R. , VanBuren, R. , Wai, C. M. , Tang, H. , Schatz, M. C. , Bowers, J. E. , Lyons, E. , Wang, M.‐L. , Chen, J. , Biggers, E. , Zhang, J. , Huang, L. , Zhang, L. , Miao, W. , Zhang, J. , Ye, Z. , Miao, C. , Lin, Z. , Wang, H. , … Yu, Q. (2015). The pineapple genome and the evolution of CAM photosynthesis. Nature Genetics, 47, 1435–1442. 26523774 10.1038/ng.3435 PMC4867222

[pld3541-bib-0133] Mukherjee, K. , Brocchieri, L. , & Bürglin, T. R. (2009). A comprehensive classification and evolutionary analysis of plant homeobox genes. Molecular Biology and Evolution, 26, 2775–2794. 19734295 10.1093/molbev/msp201 PMC2775110

[pld3541-bib-0134] Müller, M. , & Munné‐Bosch, S. (2015). Ethylene response factors: A key regulatory hub in hormone and stress signaling. Plant Physiology, 169, 32–41. 26103991 10.1104/pp.15.00677 PMC4577411

[pld3541-bib-0135] Nakano, T. , Suzuki, K. , Fujimura, T. , & Shinshi, H. (2006). Genome‐wide analysis of the ERF gene family in Arabidopsis and rice. Plant Physiology, 140, 411–432. 16407444 10.1104/pp.105.073783 PMC1361313

[pld3541-bib-0136] Ning, Y.‐Q. , Chen, Q. , Lin, R.‐N. , Li, Y.‐Q. , Li, L. , Chen, S. , & He, X.‐J. (2019). The HDA19 histone deacetylase complex is involved in the regulation of flowering time in a photoperiod‐dependent manner. The Plant Journal, 98, 448–464. 30828924 10.1111/tpj.14229

[pld3541-bib-0137] Nueda, M. J. , Tarazona, S. , & Conesa, A. (2014). Next maSigPro: Updating maSigPro bioconductor package for RNA‐seq time series. Bioinformatics, 30, 2598–2602. 24894503 10.1093/bioinformatics/btu333 PMC4155246

[pld3541-bib-0138] Ogawara, T. , Higashi, K. , Kamada, H. , & Ezura, H. (2003). Ethylene advances the transition from vegetative growth to flowering in Arabidopsis thaliana. Journal of Plant Physiology, 160, 1335–1340. 10.1078/0176-1617-01129 14658386

[pld3541-bib-0139] Ohshima, S. , Murata, M. , Sakamoto, W. , Ogura, Y. , & Motoyoshi, F. (1997). Cloning and molecular analysis of the Arabidopsis gene terminal flower 1. Molecular and General Genetics MGG, 254, 186–194. 10.1007/s004380050407 9108281

[pld3541-bib-0140] Okimoto, M. C. (1948). Anatomy and histology of the pineapple inflorescence and fruit. Botanical Gazette, 110, 217–231. 10.1086/335530

[pld3541-bib-0141] O'Malley, R. C. , Rodriguez, F. I. , Esch, J. J. , Binder, B. M. , O'Donnell, P. , Klee, H. J. , & Bleecker, A. B. (2005). Ethylene‐binding activity, gene expression levels, and receptor system output for ethylene receptor family members from Arabidopsis and tomato†. The Plant Journal, 41, 651–659. 15703053 10.1111/j.1365-313X.2004.02331.x

[pld3541-bib-0142] Osadchuk, K. , Cheng, C.‐L. , & Irish, E. E. (2019). Jasmonic acid levels decline in advance of the transition to the adult phase in maize. Plant Direct, 3, e00180. 31788658 10.1002/pld3.180 PMC6879778

[pld3541-bib-0143] Périlleux, C. , Bouché, F. , Randoux, M. , & Orman‐Ligeza, B. (2019). Turning meristems into fortresses. Trends in Plant Science, 24, 431–442. 30853243 10.1016/j.tplants.2019.02.004

[pld3541-bib-0144] Pfluger, J. , & Wagner, D. (2007). Histone modifications and dynamic regulation of genome accessibility in plants. Current Opinion in Plant Biology, 10, 645–652. 17884714 10.1016/j.pbi.2007.07.013 PMC2140274

[pld3541-bib-0145] Pin, P. A. , & Nilsson, O. (2012). The multifaceted roles of FLOWERING LOCUS T in plant development. Plant, Cell & Environment, 35, 1742–1755. 10.1111/j.1365-3040.2012.02558.x 22697796

[pld3541-bib-0146] Plett, J. M. , Cvetkovska, M. , Makenson, P. , Xing, T. , & Regan, S. (2009). Arabidopsis ethylene receptors have different roles in Fumonisin B1‐induced cell death. Physiological and Molecular Plant Pathology, 74, 18–26.

[pld3541-bib-0147] Plett, J. M. , Mathur, J. , & Regan, S. (2009). Ethylene receptor ETR2 controls trichome branching by regulating microtubule assembly in *Arabidopsis thaliana* . Journal of Experimental Botany, 60, 3923–3933. 19648171 10.1093/jxb/erp228 PMC2736899

[pld3541-bib-0148] Prusinkiewicz, P. , Zhang, T. , Owens, A. , Cieslak, M. , & Elomaa, P. (2022). Phyllotaxis without symmetry: What can we learn from flower heads? Journal of Experimental Botany, 73, 3319–3329. 35275600 10.1093/jxb/erac101 PMC9162182

[pld3541-bib-0149] Putterill, J. , Laurie, R. , & Macknight, R. (2004). It's time to flower: The genetic control of flowering time. BioEssays, 26, 363–373. 15057934 10.1002/bies.20021

[pld3541-bib-0150] Putterill, J. , Robson, F. , Lee, K. , Simon, R. , & Coupland, G. (1995). The CONSTANS gene of arabidopsis promotes flowering and encodes a protein showing similarities to zinc finger transcription factors. Cell, 80, 847–857. 7697715 10.1016/0092-8674(95)90288-0

[pld3541-bib-0151] Qian, F. , Zhao, Q.‐Y. , Zhang, T.‐N. , Li, Y.‐L. , Su, Y.‐N. , Li, L. , Sui, J.‐H. , Chen, S. , & He, X.‐J. (2021). A histone H3K27me3 reader cooperates with a family of PHD finger‐containing proteins to regulate flowering time in Arabidopsis. Journal of Integrative Plant Biology, 63, 787–802. 33433058 10.1111/jipb.13067

[pld3541-bib-0152] Qu, X. , Hall, B. P. , Gao, Z. , & Schaller, G. E. (2007). A strong constitutive ethylene‐response phenotype conferred on Arabidopsis plants containing null mutations in the ethylene receptors ETR1 and ERS1. BMC Plant Biology, 7, 3. 17224067 10.1186/1471-2229-7-3 PMC1781942

[pld3541-bib-0181] R Core Team . (2021). R: A language and environment for statistical computing. Vienna: R Foundation for Statistical Computing. https://www.R-project.org

[pld3541-bib-0153] Remizowa, M. V. , Sokoloff, D. D. , & Rudall, P. J. (2010). Evolutionary history of the monocot flower. Annals of the Missouri Botanical Garden, 97(617–645), 629.

[pld3541-bib-0154] Rivière, Q. , Corso, M. , Ciortan, M. , Noël, G. , Verbruggen, N. , & Defrance, M. (2022). Exploiting genomic features to improve the prediction of transcription factor‐binding sites in plants. Plant & Cell Physiology, 63, 1457–1473. 10.1093/pcp/pcac095 35799371

[pld3541-bib-0155] Robinson, M. D. , McCarthy, D. J. , & Smyth, G. K. (2010). edgeR: A bioconductor package for differential expression analysis of digital gene expression data. Bioinformatics, 26, 139–140. 19910308 10.1093/bioinformatics/btp616 PMC2796818

[pld3541-bib-0156] Rodriguez, A. G. (1932). Influence of smoke and ethylene on the fruiting of the pineapple (*Ananas sativus* Shult.). Journal of the Department of Agriculture. Puerto Rico, 16, 5–18.

[pld3541-bib-0157] Roosjen, M. , Paque, S. , & Weijers, D. (2017). Auxin response factors: Output control in auxin biology. Journal of Experimental Botany, 69, 179–188. 10.1093/jxb/erx237 28992135

[pld3541-bib-0158] Ruan, C. , Nian, Y. , Hu, F. , Wang, X. , Fan, H. , Wu, F. , Chen, Z. , & Zhang, Z. (2019). Cloning and expression analysis of FT gene in the process of ethephon‐induced pineapple flowering. Journal of Fruit Tree, 36, 1648–1657.

[pld3541-bib-0159] Ruelens, P. , De Maagd, R. A. , Proost, S. , Theißen, G. , Geuten, K. , & Kaufmann, K. (2013). FLOWERING LOCUS C in monocots and the tandem origin of angiosperm‐specific MADS‐box genes. Nature Communications, 4, 2280. 10.1038/ncomms3280 23955420

[pld3541-bib-0160] Sacharowski, S. P. , Gratkowska, D. M. , Sarnowska, E. A. , Kondrak, P. , Jancewicz, I. , Porri, A. , Bucior, E. , Rolicka, A. T. , Franzen, R. , Kowalczyk, J. , Pawlikowska, K. , Huettel, B. , Torti, S. , Schmelzer, E. , Coupland, G. , Jerzmanowski, A. , Koncz, C. , & Sarnowski, T. J. (2015). SWP73 subunits of Arabidopsis SWI/SNF chromatin remodeling complexes play distinct roles in leaf and flower development. The Plant Cell, 27, 1889–1906. 26106148 10.1105/tpc.15.00233 PMC4531355

[pld3541-bib-0161] Sauquet, H. , von Balthazar, M. , Magallón, S. , Doyle, J. A. , Endress, P. K. , Bailes, E. J. , Barroso de Morais, E. , Bull‐Hereñu, K. , Carrive, L. , Chartier, M. , Chomicki, G. , Coiro, M. , Cornette, R. , El Ottra, J. H. L. , Epicoco, C. , Foster, C. S. P. , Jabbour, F. , Haevermans, A. , Haevermans, T. , … Schönenberger, J. (2017). The ancestral flower of angiosperms and its early diversification. Nature Communications, 8, 16047. 10.1038/ncomms16047 PMC5543309 28763051

[pld3541-bib-0162] Sayou, C. , Monniaux, M. , Nanao, M. H. , Moyroud, E. , Brockington, S. F. , Thévenon, E. , Chahtane, H. , Warthmann, N. , Melkonian, M. , Zhang, Y. , Wong, G. K.‐S. , Weigel, D. , Parcy, F. , & Dumas, R. (2014). A promiscuous intermediate underlies the evolution of LEAFY DNA binding specificity. Science, 343, 645–648. 24436181 10.1126/science.1248229

[pld3541-bib-0163] Schaller, G. E. , & Kieber, J. J. (2002). Ethylene, 2002. The Arabidopsis Book. 10.1199/tab.0071 PMC3243340 22303216

[pld3541-bib-0164] Schilling, S. , Kennedy, A. , Pan, S. , Jermiin, L. S. , & Melzer, R. (2020). Genome‐wide analysis of MIKC‐type MADS‐box genes in wheat: Pervasive duplications, functional conservation and putative neofunctionalization. New Phytologist, 225, 511–529. 31418861 10.1111/nph.16122

[pld3541-bib-0166] Schmitz, R. J. , Grotewold, E. , & Stam, M. (2021). Cis‐regulatory sequences in plants: Their importance, discovery, and future challenges. The Plant Cell, 34, 718–741. 10.1093/plcell/koab281 PMC8824567 34918159

[pld3541-bib-0167] Searle, I. , He, Y. , Turck, F. , Vincent, C. , Fornara, F. , Kröber, S. , Amasino, R. A. , & Coupland, G. (2006). The transcription factor FLC confers a flowering response to vernalization by repressing meristem competence and systemic signaling in Arabidopsis. Genes & Development, 20, 898–912. 16600915 10.1101/gad.373506 PMC1472290

[pld3541-bib-0168] Sebastian, A. , & Contreras‐Moreira, B. (2013). footprintDB: A database of transcription factors with annotated cis elements and binding interfaces. Bioinformatics, 30, 258–265. 24234003 10.1093/bioinformatics/btt663

[pld3541-bib-0169] Seifert, G. J. , Barber, C. , Wells, B. , & Roberts, K. (2004). Growth regulators and the control of nucleotide sugar flux. The Plant Cell, 16, 723–730. 14973160 10.1105/tpc.019661 PMC385283

[pld3541-bib-0170] Shim, J. S. , Kubota, A. , & Imaizumi, T. (2016). Circadian clock and photoperiodic flowering in Arabidopsis: CONSTANS is a hub for signal integration. Plant Physiology, 173, 5–15. 27688622 10.1104/pp.16.01327 PMC5210731

[pld3541-bib-0171] Smoczynska, A. , & Szweykowska‐Kulinska, Z. (2016). MicroRNA‐mediated regulation of flower development in grasses. Acta Biochimica Polonica, 63, 687–692. 27815966 10.18388/abp.2016_1358

[pld3541-bib-0172] Smyth, D. R. (2018). Evolution and genetic control of the floral ground plan. New Phytologist, 220, 70–86. 29959892 10.1111/nph.15282

[pld3541-bib-0174] Song, Y. H. , Ito, S. , & Imaizumi, T. (2010). Similarities in the circadian clock and photoperiodism in plants. Current Opinion in Plant Biology, 13, 594–603. 20620097 10.1016/j.pbi.2010.05.004 PMC2965781

[pld3541-bib-0175] Srikanth, A. , & Schmid, M. (2011). Regulation of flowering time: All roads lead to Rome. Cellular and Molecular Life Sciences, 68, 2013–2037. 21611891 10.1007/s00018-011-0673-y PMC11115107

[pld3541-bib-0176] Stewart, D. , Graciet, E. , & Wellmer, F. (2016). Molecular and regulatory mechanisms controlling floral organ development. The FEBS Journal, 283, 1823–1830. 26725470 10.1111/febs.13640

[pld3541-bib-0180] Taoka, K.‐i. , Ohki, I. , Tsuji, H. , Furuita, K. , Hayashi, K. , Yanase, T. , Yamaguchi, M. , Nakashima, C. , Purwestri, Y. A. , Tamaki, S. , Ogaki, Y. , Shimada, C. , Nakagawa, A. , Kojima, C. , & Shimamoto, K. (2011). 14‐3‐3 proteins act as intracellular receptors for rice Hd3a florigen. Nature, 476, 332–335. 21804566 10.1038/nature10272

[pld3541-bib-0182] Teotia, S. , & Tang, G. (2015). To bloom or not to bloom: Role of microRNAs in plant flowering. Molecular Plant, 8, 359–377. 25737467 10.1016/j.molp.2014.12.018

[pld3541-bib-0183] Tieman, D. M. , Taylor, M. G. , Ciardi, J. A. , & Klee, H. J. (2000). The tomato ethylene receptors NR and LeETR4 are negative regulators of ethylene response and exhibit functional compensation within a multigene family. Proceedings of the National Academy of Sciences, 97, 5663–5668. 10.1073/pnas.090550597 PMC25885 10792050

[pld3541-bib-0184] Tourdot, E. , Mauxion, J.‐P. , Gonzalez, N. , & Chevalier, C. (2023). Endoreduplication in plant organogenesis: A mean to boost fruit growth. Journal of Experimental Botany, erad235. 10.1093/jxb/erad235 37343125

[pld3541-bib-0185] Trusov, Y. , & Botella, J. R. (2006). Silencing of the ACC synthase gene ACACS2 causes delayed flowering in pineapple [*Ananas comosus* (L.) Merr.]. Journal of Experimental Botany, 57, 3953–3960. 17046980 10.1093/jxb/erl167

[pld3541-bib-0186] Turatsinze, J.‐V. , Thomas‐Chollier, M. , Defrance, M. , & van Helden, J. (2008). Using RSAT to scan genome sequences for transcription factor binding sites and cis‐regulatory modules. Nature Protocols, 3, 1578–1588. 18802439 10.1038/nprot.2008.97

[pld3541-bib-0188] Wang, F. , Cui, X. , Sun, Y. , & Dong, C.‐H. (2013). Ethylene signaling and regulation in plant growth and stress responses. Plant Cell Reports, 32, 1099–1109. 23525746 10.1007/s00299-013-1421-6

[pld3541-bib-0189] Wang, J. , Wu, D. , Wang, Y. , & Xie, D. (2019). Jasmonate action in plant defense against insects. Journal of Experimental Botany, 70, 3391–3400. 30976791 10.1093/jxb/erz174

[pld3541-bib-0190] Wang, L. , Li, Y. , Jin, X. , Liu, L. , Dai, X. , Liu, Y. , Zhao, L. , Zheng, P. , Wang, X. , Liu, Y. , Lin, D. , & Qin, Y. (2020). Floral transcriptomes reveal gene networks in pineapple floral growth and fruit development. Communications Biology, 3, 500. 32913289 10.1038/s42003-020-01235-2 PMC7483743

[pld3541-bib-0191] Wang, M.‐L. , & Paull, R. E. (2018). Genetic transformation of pineapple. In R. Ming (Ed.), Genetics and genomics of pineapple (pp. 69–86). Springer International Publishing.

[pld3541-bib-0192] Wang, P. , Hsu, C.‐C. , Du, Y. , Zhu, P. , Zhao, C. , Fu, X. , Zhang, C. , Paez, J. S. , Macho, A. P. , Tao, W. A. , & Zhu, J.‐K. (2020). Mapping proteome‐wide targets of protein kinases in plant stress responses. Proceedings of the National Academy of Sciences, 117, 3270–3280. 10.1073/pnas.1919901117 PMC7022181 31992638

[pld3541-bib-0193] Wang, Q. , Zhang, W. , Yin, Z. , & Wen, C.‐K. (2013). Rice CONSTITUTIVE TRIPLE‐RESPONSE2 is involved in the ethylene‐receptor signalling and regulation of various aspects of rice growth and development. Journal of Experimental Botany, 64, 4863–4875. 24006427 10.1093/jxb/ert272 PMC3830475

[pld3541-bib-0197] Wang, Z. , & Gou, X. (2020). Receptor‐like protein kinases function upstream of MAPKs in regulating plant development. International Journal of Molecular Sciences, 21, 7638. 33076465 10.3390/ijms21207638 PMC7590044

[pld3541-bib-0198] Weigel, D. , Alvarez, J. , Smyth, D. R. , Yanofsky, M. F. , & Meyerowitz, E. M. (1992). LEAFY controls floral meristem identity in Arabidopsis. Cell, 69, 843–859. 1350515 10.1016/0092-8674(92)90295-n

[pld3541-bib-0199] Weigel, D. , & Meyerowitz, E. M. (1994). The ABCs of floral homeotic genes. Cell, 78, 203–209. 7913881 10.1016/0092-8674(94)90291-7

[pld3541-bib-0200] Wen, C.‐K. , Li, W. , & Guo, H. (2015). Regulatory components of ethylene signal transduction. In C.‐K. Wen (Ed.), Ethylene in plants (pp. 73–92). Springer Netherlands.

[pld3541-bib-0201] Wickland Daniel, P. , & Hanzawa, Y. (2015). The FLOWERING LOCUS T/TERMINAL FLOWER 1 gene family: Functional evolution and molecular mechanisms. Molecular Plant, 8, 983–997. 25598141 10.1016/j.molp.2015.01.007

[pld3541-bib-0202] Wigge, P. A. , Kim, M. C. , Jaeger, K. E. , Busch, W. , Schmid, M. , Lohmann, J. U. , & Weigel, D. (2005). Integration of spatial and temporal information during floral induction in *Arabidopsis* . Science, 309, 1056–1059. 16099980 10.1126/science.1114358

[pld3541-bib-0203] Wilson, R. L. , Kim, H. , Bakshi, A. , & Binder, B. M. (2014). The ethylene receptors ETHYLENE RESPONSE1 and ETHYLENE RESPONSE2 have contrasting roles in seed germination of Arabidopsis during salt stress. Plant Physiology, 165, 1353–1366. 24820022 10.1104/pp.114.241695 PMC4081342

[pld3541-bib-0204] Wu, F. , Shi, X. , Lin, X. , Liu, Y. , Chong, K. , Theißen, G. , & Meng, Z. (2017). The ABCs of flower development: Mutational analysis of AP1/FUL‐like genes in rice provides evidence for a homeotic (A)‐function in grasses. The Plant Journal, 89, 310–324. 27689766 10.1111/tpj.13386

[pld3541-bib-0205] Wu, K. , Zhang, L. , Zhou, C. , Yu, C.‐W. , & Chaikam, V. (2008). HDA6 is required for jasmonate response, senescence and flowering in Arabidopsis. Journal of Experimental Botany, 59, 225–234. 18212027 10.1093/jxb/erm300

[pld3541-bib-0206] Wuriyanghan, H. , Zhang, B. , Cao, W.‐H. , Ma, B. , Lei, G. , Liu, Y.‐F. , Wei, W. , Wu, H.‐J. , Chen, L.‐J. , Chen, H.‐W. , Cao, Y.‐R. , He, S.‐J. , Zhang, W.‐K. , Wang, X.‐J. , Chen, S.‐Y. , & Zhang, J.‐S. (2009). The ethylene receptor ETR2 delays floral transition and affects starch accumulation in rice. The Plant Cell, 21, 1473–1494. 19417056 10.1105/tpc.108.065391 PMC2700534

[pld3541-bib-0207] Wybouw, B. , & De Rybel, B. (2019). Cytokinin—A developing story. Trends in Plant Science, 24, 177–185. 30446307 10.1016/j.tplants.2018.10.012

[pld3541-bib-0208] Xie, F. , Liu, Q. , & Wen, C.‐K. (2006). Receptor signal output mediated by the ETR1 N terminus is primarily subfamily I receptor dependent. Plant Physiology, 142, 492–508. 16891553 10.1104/pp.106.082628 PMC1586051

[pld3541-bib-0209] Xu, H. , Yu, Q. , Shi, Y. , Hua, X. , Tang, H. , Yang, L. , Ming, R. , & Zhang, J. (2018). PGD: Pineapple genomics database. Horticulture Research, 5, 66. 10.1038/s41438-018-0078-2 30245835 PMC6139296

[pld3541-bib-0210] Yamasaki, K. , Kigawa, T. , Inoue, M. , Tateno, M. , Yamasaki, T. , Yabuki, T. , Aoki, M. , Seki, E. , Matsuda, T. , Nunokawa, E. , Ishizuka, Y. , Terada, T. , Shirouzu, M. , Osanai, T. , Tanaka, A. , Seki, M. , Shinozaki, K. , & Yokoyama, S. (2004). A novel zinc‐binding motif revealed by solution structures of DNA‐binding domains of Arabidopsis SBP‐family transcription factors. Journal of Molecular Biology, 337, 49–63. 15001351 10.1016/j.jmb.2004.01.015

[pld3541-bib-0211] Yang, D.‐L. , Yao, J. , Mei, C.‐S. , Tong, X.‐H. , Zeng, L.‐J. , Li, Q. , Xiao, L.‐T. , Sun, T.‐p. , Li, J. , Deng, X.‐W. , Lee, C. M. , Thomashow, M. F. , Yang, Y. , He, Z. , & He, S. Y. (2012). Plant hormone jasmonate prioritizes defense over growth by interfering with gibberellin signaling cascade. Proceedings of the National Academy of Sciences, 109, E1192–E1200. 10.1073/pnas.1201616109 PMC3358897 22529386

[pld3541-bib-0212] Yang, F. , Liang, G. , Liu, D. , & Yu, D. (2009). Arabidopsis MiR396 mediates the development of leaves and flowers in transgenic tobacco. Journal of Plant Biology, 52, 475–481.

[pld3541-bib-0213] Yano, M. , Katayose, Y. , Ashikari, M. , Yamanouchi, U. , Monna, L. , Fuse, T. , Baba, T. , Yamamoto, K. , Umehara, Y. , Nagamura, Y. , & Sasaki, T. (2000). Hd1, a major photoperiod sensitivity quantitative trait locus in rice, is closely related to the Arabidopsis flowering time gene CONSTANS. The Plant Cell, 12, 2473–2483. 11148291 10.1105/tpc.12.12.2473 PMC102231

[pld3541-bib-0214] Yoshida, H. , & Nagato, Y. (2011). Flower development in rice. Journal of Experimental Botany, 62, 4719–4730. 21914655 10.1093/jxb/err272

[pld3541-bib-0215] Yu, C.‐P. , Lin, J.‐J. , & Li, W.‐H. (2016). Positional distribution of transcription factor binding sites in Arabidopsis thaliana. Scientific Reports, 6, 25164. 27117388 10.1038/srep25164 PMC4846880

[pld3541-bib-0216] Yu, C.‐W. , Liu, X. , Luo, M. , Chen, C. , Lin, X. , Tian, G. , Lu, Q. , Cui, Y. , & Wu, K. (2011). HISTONE DEACETYLASE6 interacts with FLOWERING LOCUS D and regulates flowering in Arabidopsis. Plant Physiology, 156, 173–184. 21398257 10.1104/pp.111.174417 PMC3091038

[pld3541-bib-0218] Yu, X. , Duan, X. , Zhang, R. , Fu, X. , Ye, L. , Kong, H. , Xu, G. , & Shan, H. (2016). Prevalent exon‐intron structural changes in the APETALA1/FRUITFULL, SEPALLATA, AGAMOUS‐LIKE6, and FLOWERING LOCUS C MADS‐box gene subfamilies provide new insights into their evolution. Frontiers in Plant Science, 7, 598. 27200066 10.3389/fpls.2016.00598 PMC4852290

[pld3541-bib-0220] Zhai, Q. , Zhang, X. , Wu, F. , Feng, H. , Deng, L. , Xu, L. , Zhang, M. , Wang, Q. , & Li, C. (2015). Transcriptional mechanism of jasmonate receptor COI1‐mediated delay of flowering time in Arabidopsis. The Plant Cell, 27, 2814–2828. 26410299 10.1105/tpc.15.00619 PMC4682329

[pld3541-bib-0221] Zhang, H. , Pan, X. , Yi, D. , Lin, W. , & Zhang, X. (2020). Role of MADS‐box gene in the development of flower organs in pineapple of a typical collective fruit. Research Square. 10.21203/rs.3.rs-53296/v1

[pld3541-bib-0222] Zhang, X. , Fatima, M. , Zhou, P. , Ma, Q. , & Ming, R. (2020). Analysis of MADS‐box genes revealed modified flowering gene network and diurnal expression in pineapple. BMC Genomics, 21, 8. 31896347 10.1186/s12864-019-6421-7 PMC6941321

[pld3541-bib-0223] Zhao, L. , Li, X. , Chen, W. , Xu, Z. , Chen, M. , Wang, H. , & Yu, D. (2021). The emerging role of jasmonate in the control of flowering time. Journal of Experimental Botany, 73, 11–21. 10.1093/jxb/erab418 34599804

[pld3541-bib-0224] Zhao, S. , Zhang, B. , Yang, M. , Zhu, J. , & Li, H. (2018). Systematic profiling of histone readers in *Arabidopsis thaliana* . Cell Reports, 22, 1090–1102. 29386129 10.1016/j.celrep.2017.12.099

[pld3541-bib-0225] Zhao, Y. , Zhang, T. , Broholm, S. K. , Tähtiharju, S. , Mouhu, K. , Albert, V. A. , Teeri, T. H. , & Elomaa, P. (2016). Evolutionary co‐option of floral meristem identity genes for patterning of the flower‐like Asteraceae inflorescence. Plant Physiology, 172, 284–296. 27382139 10.1104/pp.16.00779 PMC5074612

[pld3541-bib-0226] Zhao, Z. , Yu, Y. , Meyer, D. , Wu, C. , & Shen, W.‐H. (2005). Prevention of early flowering by expression of FLOWERING LOCUS C requires methylation of histone H3 K36. Nature Cell Biology, 7, 1256–1260. 16299497 10.1038/ncb1329

[pld3541-bib-0227] Zheng, Y. , Li, T. , Xu, Z. , Wai, C. M. , Chen, K. , Zhang, X. , Wang, S. , Ji, B. , Ming, R. , & Sunkar, R. (2016). Identification of microRNAs, phasiRNAs and their targets in pineapple. Tropical Plant Biology, 9, 176–186.

[pld3541-bib-0228] Zhou, C. , Zhang, L. , Duan, J. , Miki, B. , & Wu, K. (2005). HISTONE DEACETYLASE19 is involved in jasmonic acid and ethylene signaling of pathogen response in Arabidopsis. The Plant Cell, 17, 1196–1204. 15749761 10.1105/tpc.104.028514 PMC1087996

[pld3541-bib-0229] Zhu, Q.‐H. , & Helliwell, C. A. (2010). Regulation of flowering time and floral patterning by miR172. Journal of Experimental Botany, 62, 487–495. 20952628 10.1093/jxb/erq295

[pld3541-bib-0230] Zhu, Y. , Klasfeld, S. , Jeong, C. W. , Jin, R. , Goto, K. , Yamaguchi, N. , & Wagner, D. (2020). TERMINAL FLOWER 1‐FD complex target genes and competition with FLOWERING LOCUS T. Nature Communications, 11, 5118. 10.1038/s41467-020-18782-1 PMC7550357 33046692

[pld3541-bib-0231] Zhu, Y. , Klasfeld, S. , & Wagner, D. (2021). Molecular regulation of plant developmental transitions and plant architecture via PEPB family proteins: An update on mechanism of action. Journal of Experimental Botany, 72, 2301–2311. 33449083 10.1093/jxb/eraa598

[pld3541-bib-0232] Zhu, Z. Y. , Yang, Y. M. , Liu, S. H. , & Sun, G. M. (2012). Natrual flowering of pineapple and the inhibited technology. China Tropical Agriculture, 48, 75–77.

