# Supplementary material for: Differential gene expression during floral transition in pineapple
Source: Plant Direct. 2023 Nov 14;7(11):e541. doi: 10.1002/pld3.541 (PMC10644199; doi:10.1002/pld3.541)
Supplement: Supplementary file 1 — Data S1. Supplementary Results SR1 DEGs associated with cytokinin synthesis and response, abscisic acid, LATE EMBRYOGENESIS ABUNDANT (LEA) genes, nodulin‐related, stress‐related, sugar metabolism, OVATE, and light‐dependent short hypocotyl, protein turnover and interactions, regulation of transport gradients, sugar metabolism and transporters, and our network and temporal analysis. [file PLD3-7-e541-s002.docx]

**Supplementary Results SR1**

***Cytokinin Synthesis and Response***

Cytokinin (CK) synthesis involves isopentenyl transferase (IPT), of which one (Aco006715) was upregulated both at the base of the leaf and the apex only on day 1. A CK response regulator (Aco002961) was down-regulated at the apex only on day 2. Then another CK response regulator (Aco010350) was down-regulated at the leaf bases, while in the apex it was down-regulated after day 3. Three genes of cytokinin oxidoreductase (Aco006318, Aco007706; Aco023264) showed variable up- and downregulation at both the apex and the leaf base. The 5'-monophosphate phosphoribohydrolase gene (LONELY GUY Genes (LOG) (Aco010086; Aco013949; Aco017730) were also differentially up or downregulated in both tissues at different sampling times.

***Abscisic acid genes***

Of the seven predicted ABA receptors (PYR1), one (Aco018101) began to increase at the apex after day 1; no similar increase was observed at the bases of the leaf. The PYR1 receptors (Aco007083) increased on day 1 in the base of the leaf, while Aco005513 was negatively regulated at the apex at 6 h and days 2 through 6. The other PYR1 genes (Aco004375) were down-regulated at the apex at 6 h and day 2. The binding factor (Aco015030) increased at the apex on day 3. The abscisic stress ripening protein Aco007857 increased twofold after treatment at the apex and leaf bases, while Aco003190 increased more than twofold on day 2 at the apex only. The GRAM domain-containing protein/ABA-responsive protein-related protein (Aco003616) increased at 6 h and day 3, 7 and 8 at the apex, but only at 6 h for the leaf bases together with Aco007675.

***Late embryogenesis abundant genes (LEA) and nodulin-related genes***

Twenty-seven predicted LEA genes showed expression at the apex and leaf bases with or without ethylene treatment. Aco002475 was up-regulated at the apex only on days 1 and 2, while Aco009627 was up-regulated in the apex on day 1. Aco012577 tended to be higher at the apex until day 5 and Ac002475 until day 6.

Three early nodule-like genes (Aco0048773; Aco007544; Aco022294) were up-regulated within 6 h and then expressed at a higher level than the untreated control at the apex until day 6. A nodulin transporter (Aco016031) was negatively regulated at the apex within 6 hours after treatment and expressed at a lower level than the control for the entire sampling period.

***Stress-Related Genes***

Noticeable changes occurred in six stress-related genes. A protein similar to heat stress transcription factor A-1 (Aco027352) was negatively regulated on days 3 and again on days 6 and 8, while another (Aco005573) was positively regulated on days 2 to 5. An early responsive protein to dehydration stress (Aco015870) was up-regulated more than 1.7 times from day 2 to day 8. A putative NBS-LRR disease resistance protein (Aco031473) was negatively regulated on day 2 and positively regulated on day 4 and then significantly negatively regulated on day 5. Other LRR DE genes were up- and down-regulated at different sampling stage (Supplementary Figure SF1). Proton gradient regulation 5-chloroplast photoprotection (Aco010143) was up-regulated on day 2 and again on day 4 to 8. A wheat-related stress-responsive homolog protein 1 (16V2S3 – expressed in the pericarp) (Aco001681) was negatively regulated during the first 3 days, then positively regulated for days 4 and 5 before negatively regulated.Cytochrome P450 genes were in the top twelve DE up- and down-regulated genes at all sampling stages while other P450s were down-regulated after day 3. (Supplementary Figure SF1).

Four Remorins, involved in the stress response, showed differential up-regulation at both the apex and the leaf bases at different sampling times. Remorin (Aco001328) was up-regulated in the leaf base at 6 h and at the apex on days 2 and 3. Aco009303 was up-regulated at the apex and down-regulated at the leaf base at 6 h, while both tissues were up-regulated on day 2. The other two Remorins (Aco013539; Aco014830), depending on the sampling, showed up- and down-regulation.

Calmodulin 4 (Aco001760) was down-regulated in the leaf base at 6 h and up-regulated in the leaf base and apex on day 2. The calcium-binding EF-hand gene (Ac0000376) was up-regulated at the apex on days 1 and 2, down-regulated in leaf 6 h and on days 3 and 4, and up-regulated on day 2.

***Regulation of the Transport Gradient***

Three proteins in the MATE efflux family involved primary and secondary metabolites, including ABA and auxin, and petal differentiation and expansion increased on day 3 alone (Aco029923) at the apex, from day 2 to day 3 (Aco000270) at the apex, and Aco009960 was not differentially expressed, although it tended to be lower than the control. A nitrate transporter (Aco015264) was down-regulated at the base of the leaf at 6 h and up-regulated at the apex from day 1 to day 8, except on day 3 when it was down-regulated. An ABC transporter G family member 14 potentially involved in cytokinin transport, Aco013155 (AcABCG27), was up-regulated at the apex only on day 3. Another member of the G family of ABC transporter (Aco020513) 16 tended to be higher than the control.

***Sugar Metabolism and Transporters***

Hexokinase (Aco000407) was negatively regulated at the apex on days 2 and 3. A sucrose phosphate synthase (Aco017378) was up-regulated at the apex on day 3. Acid invertase (Aco-17533) was differentially down-regulated on days 2 and 3.

Trehalose phosphate synthase (Aco000950) was negatively regulated at the apex at 6 h and on days 2, 3 and 6, and at the base of the leaf at 6 h. Aco012107 was down-regulated at the apex and leaf base on day 3 and up-regulated at the apex on day 5 and was down-regulated again on day 8. Trehalose-6-phosphate phosphatase involved in starch and sucrose metabolism showed different patterns. Three trehalose-6-phosphate phosphatases were differentially regulated; Aco001192 was up-regulated at the apex on day 3, Aco004091 was down-regulated at the apex on day 3; Aco011727 and Aco014723 were down-regulated at the base of the leaf at 6 h.

The expression of the callose-degrading enzyme, glucan endo-1,3-beta-glucosidase 10 (Aco002643) was up-regulated at 6 h and then down-regulated on day 1 at the base of the leaf, while up-regulated at the apex on days 2 and 3, and 6 and 8.

Different alleles of monosaccharide transporter (MST) symporters showed variable patterns. Two MSTs, Aco003836, were up-regulated in the leaf base on days 1, 3 and 4, and up-regulated at the apex on days 2, 3 and 6; Aco10302 was down-regulated in the leaf base at 6 h, and up-regulated in the apex on days 2, 3 and 4; Aco008059 was down-regulated in the apex on day 3. The other solute carrier Aco014404 was negatively regulated at the apex and the leaf base was positively regulated at 6 h. The bidirectional sugar transporter (Aco011302) was down-regulated on days 3 and 5 at the apex, while Aco005793 was down-regulated in the leaf on day 2, and up-regulated in the apex on days 3, 6 and 8.

***Ovate and Light-Dependent Short Hypocotyl Gene Expression***

Four Ovate genes in pineapple were up-regulated at different sampling times after ethylene treatment. Ovate modulates the shape of the tomato and may act as transcription repressors in other systems. Ovate (Aco011137) was up-regulated at the apex and down-regulated in the leaf 6 hours after treatment and down-regulated on days 6 and 8 days at the apex. The other three Ovate genes (Aco016755; Aco018275; Aco029040) were all up-regulated from day 1 to day 4 at the apex, and days 3 and 4 in the leaf, although Aco018275 was only up-regulated at the apex, while Aco029040 on days 1 to 3 at the apex.

Two light-dependent short hypocotyl genes (Ac008960; Aco031856), involved in plant morphogenesis such as seedling stage, were both up-regulated at the apex on day 1 and in the leaf 6 hours after treatment and in both tissues from day 2 to 4.

***Protein Turnover and Interactions***

Five RING/U and -H2 finger genes (Aco009665; Aco012901; Aco018868; Aco022305; Aco026964) associated with protein ubiquitination and an acceptor of RAB in the secretory and endocytic trafficking gene (Aco001601) showed a cluster of similar upregulation in the first three days after ethylene treatment, two were upregulated from very low expression levels in untreated control samples within 6 hours of treatment (Aco018868; Ac022305). A RING finger protein (Aco010501) had lower expression in subsequent samplings (6 and 8 days). A protein in the BTB / POZ domain (Aco018361) that mediated transcriptional repression and interacts with histone deacetylase decreased in 6 h at the apex and remained lower than the control until day 8.

**Network and Temporal Analysis**

The central nodes in the first module of the zero-day sampling taken 6 hours after ethephon treatment (Supplementary Figure SF5) include several unknowns (Aco002616; Aco008737; Aco014170; Aco016785; Aco022058; Aco023348). A principal node included an GA 2-oxidase involved in GA degradation (Aco002580), leucine-rich repeat protein kinase (Aco021558), a RING/U finger genes involved in protein turnover (Aco018868), sucrose phosphate synthase (Aco017378), a cytoskeleton-related formin (Aco004882) and an abundance of late embryogenesis proteins (LEA5) (Aco002475). Furthermore, several TFs are involved in this network; Ethylene responsive transcription factor 3 (Aco004208), two WRKY DNA binding proteins 51 and 74 (Aco005719; Aco005240), GRAS family transcription factor (Aco012050), bHLH DNA binding protein (Aco016415), a MYB domain protein (Ac0031816), GATA transcription factor (Aco009467) and a MADS box transcription factor (Aco019039).

When plants were treated with ethylene, there was a clear response relative to the control. Not unexpectedly, the transcriptional networks that were activated directly after treatment (6 hours) contained transcription factors and ethylene response genes (Supplemental Figure SF1, SF4). These genes started a cascade that could lead to floral induction. On the contrary, at the control apex, there was fewer transcription factors and networked genes, in general (Supplemental Table ST5). These networks of response genes become less pronounced in the latter sampling, clearly showing that the cascade of effects occurs very rapidly after initiation.

The GWENA coexpression analysis found a similar number of genes as in previous studies in different systems. However, care must be taken in overinterpreting the biological meaning of the coexpression, as the main objective of this analysis was to identify potential targets for future analysis. We found key pathways that contain important genes that are expressed together at key time points after floral initiation, which coincided with differential expression analysis. Furthermore, in general, less than 25% of the genes in the GWENA coexpression group were differentially expressed (Supplementary Table ST5). The highest percentage found in any coexpression group was 51% in a group of 29 members.

**Discussion**

A surprising observation was the up-regulation of four OVATE genes at the pineapple apex at different stages. An OVATE gene (Aco011137) was up-regulated at the apex and down-regulated at the leaf base at 6 hours and down-regulated at 6 and 8 days. OVATE genes with a conserved domain, initially described in controlling tomato shape (Liu *et al.*, 2002), act as transcription repressors in many aspects of plant growth and development (Wang *et al.*, 2016) and the expression of target genes. One such case is Gibberellin 20 oxidase (GA20ox) (Wang *et al.*, 2007), although this GA20ox gene was not differentially expressed at the apex of the pineapple. In addition to its response in tomato shape. Liu *et al.* (2002) noted abnormalities such as reduced floral organ size, dwarf plants, and changes in leaf morphology. Ovate expression is found in all tissues and organs in tomato, rice, and Arabidopsis (Schmitz *et al.*, 2015; Wang *et al.*, 2011) and in rice especially during seed development (Yu *et al.*, 2015). The role of light-dependent short hypocotyl genes (Aco008960; Aco031856) was up-regulated at the apex and leaf base from day 1 to day 4. Some of these light-dependent short hypocotyl genes are involved in organ boundary determination (Cho and Zambryski, 2011; Lee *et al.*, 2020; Takeda *et al.*, 2011) that would be required in the vegetative to floral transition.

Meristem fate is determined by many interacting pathways (Claeys *et al.*, 2019; Tanaka *et al.*, 2013). One of these pathways involves trehalose 6-phosphate phosphatases (Claeys *et al.*, 2019). In pineapple, trehalose 6-phosphate phosphatase (Aco001192) was up-regulated at the apex on day 3 while another allele (Aco004091) was down-regulated. Trehalose 6-phosphate phosphatases are correlated with sucrose levels and, therefore, may be required to ensure adequate levels for changes in tissue and development stage (Figueroa and Lunn, 2016). However, at the apex of pineapple, the sugar metabolic pathways and sugar transporters did not show a relationship with the vegetative to flowering meristem transition.

**References Cited**

**Cho E, Zambryski PC**. 2011. *ORGAN BOUNDARY1* defines a gene expressed at the junction between the shoot apical meristem and lateral organs. Proceedings of the National Academy of Sciences **108**, 2154-2159.

**Claeys H, Vi SL, Xu X, Satoh-Nagasawa N, Eveland AL, Goldshmidt A, Feil R, Beggs GA, Sakai H, Brennan RG, Lunn JE, Jackson D**. 2019. Control of meristem determinacy by trehalose 6-phosphate phosphatases is uncoupled from enzymatic activity. Nature Plants **5**, 352-357.

**Figueroa CM, Lunn JE**. 2016. A Tale of Two Sugars: Trehalose 6-Phosphate and Sucrose. Plant Physiology **172**, 7-27.

**Lee M, Dong X, Song H, Yang JY, Kim S, Hur Y**. 2020. Molecular characterization of *Arabidopsis thaliana* LSH1 and LSH2 genes. Genes & Genomics **42**, 1151-1162.

**Liu J, Van Eck J, Cong B, Tanksley SD**. 2002. A new class of regulatory genes underlying the cause of pear-shaped tomato fruit. Proceedings of the National Academy of Sciences **99**, 13302-13306.

**Schmitz AJ, Begcy K, Sarath G, Walia H**. 2015. Rice Ovate Family Protein 2 (OFP2) alters hormonal homeostasis and vasculature development. Plant Science **241**, 177-188.

**Takeda S, Hanano K, Kariya A, Shimizu S, Zhao L, Matsui M, Tasaka M, Aida M**. 2011. CUP-SHAPED COTYLEDON1 transcription factor activates the expression of LSH4 and LSH3, two members of the ALOG gene family, in shoot organ boundary cells. The Plant Journal **66**, 1066-1077.

**Tanaka W, Pautler M, Jackson D, Hirano H-Y**. 2013. Grass Meristems II: Inflorescence Architecture, Flower Development and Meristem Fate. Plant and Cell Physiology **54**, 313-324.

**Wang S, Chang Y, Ellis B**. 2016. Overview of OVATE FAMILY PROTEINS, A Novel Class of Plant-Specific Growth Regulators. Frontiers in Plant Science **7,** 417

**Wang S, Chang Y, Guo J, Chen J-G**. 2007. Arabidopsis Ovate Family Protein 1 is a transcriptional repressor that suppresses cell elongation. The Plant Journal **50**, 858-872.

**Wang S, Chang Y, Guo J, Zeng Q, Ellis BE, Chen J-G**. 2011. Arabidopsis Ovate Family Proteins, a Novel Transcriptional Repressor Family, Control Multiple Aspects of Plant Growth and Development. PLOS ONE **6**, e23896.

**Yu H, Jiang W, Liu Q, Zhang H, Piao M, Chen Z, Bian M**. 2015. Expression Pattern and Subcellular Localization of the Ovate Protein Family in Rice. PLOS ONE **10**, e0118966.
