# Supplementary material for: Differential gene expression during floral transition in pineapple
Source: Plant Direct. 2023 Nov 14;7(11):e541. doi: 10.1002/pld3.541 (PMC10644199; doi:10.1002/pld3.541)

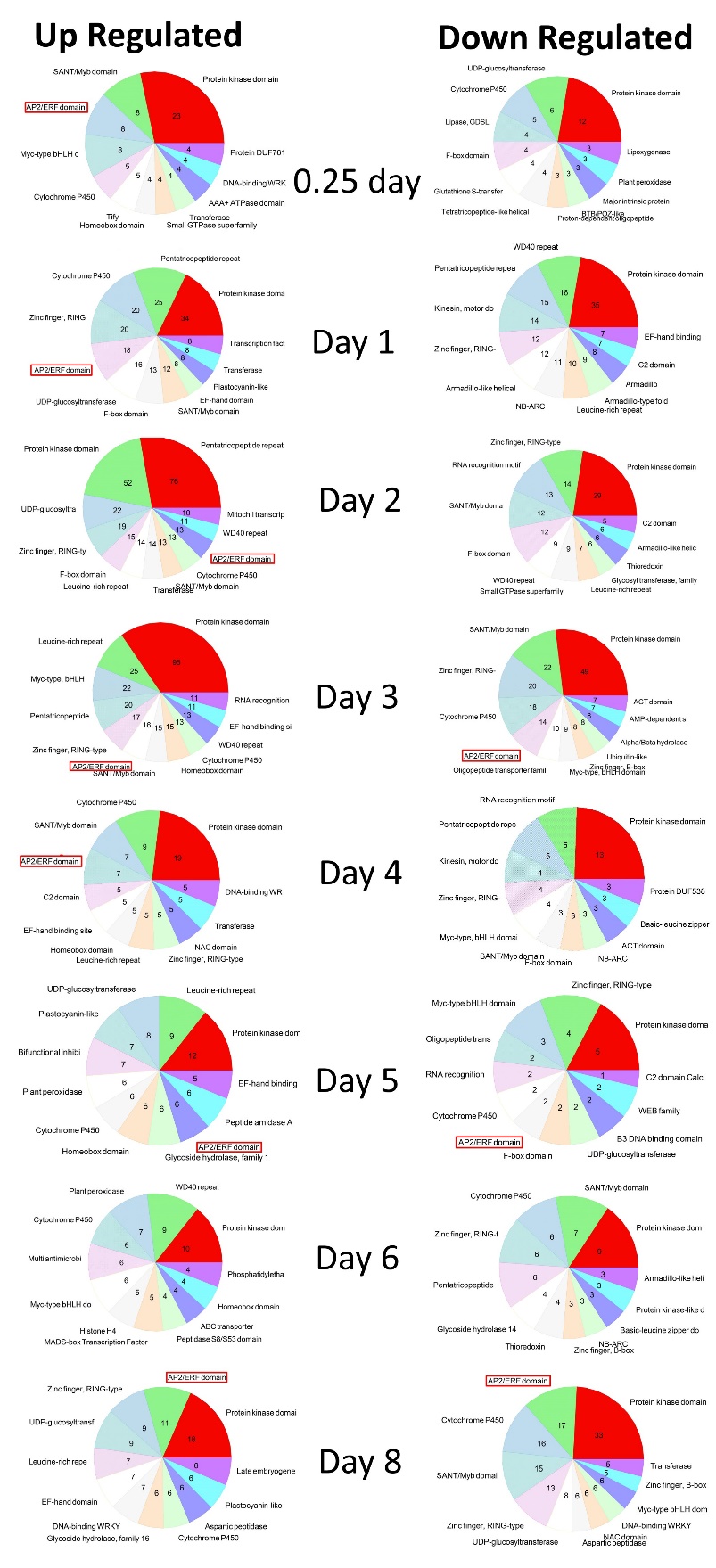
**Supplementary Figure SF1.** InterPro domain motifs of the top twelve up and down-regulated differentially expressed genes. The AP2-ERTF genes in the individual Pie Charts are boxed in a red rectangle. The number in each pie slice are the number of genes that were differential expressed at that sampling. Full data available in Supplementary Table ST2.

**Supplementary Figure SF2.** **Pineapple flowering transcriptome library quality A. Total number of r**eads, B. Normalized Log_2_ counts per million, and C. Assignment of total reads as percent of total from control and treated apex and leaf bases at different sampling times**.**

**
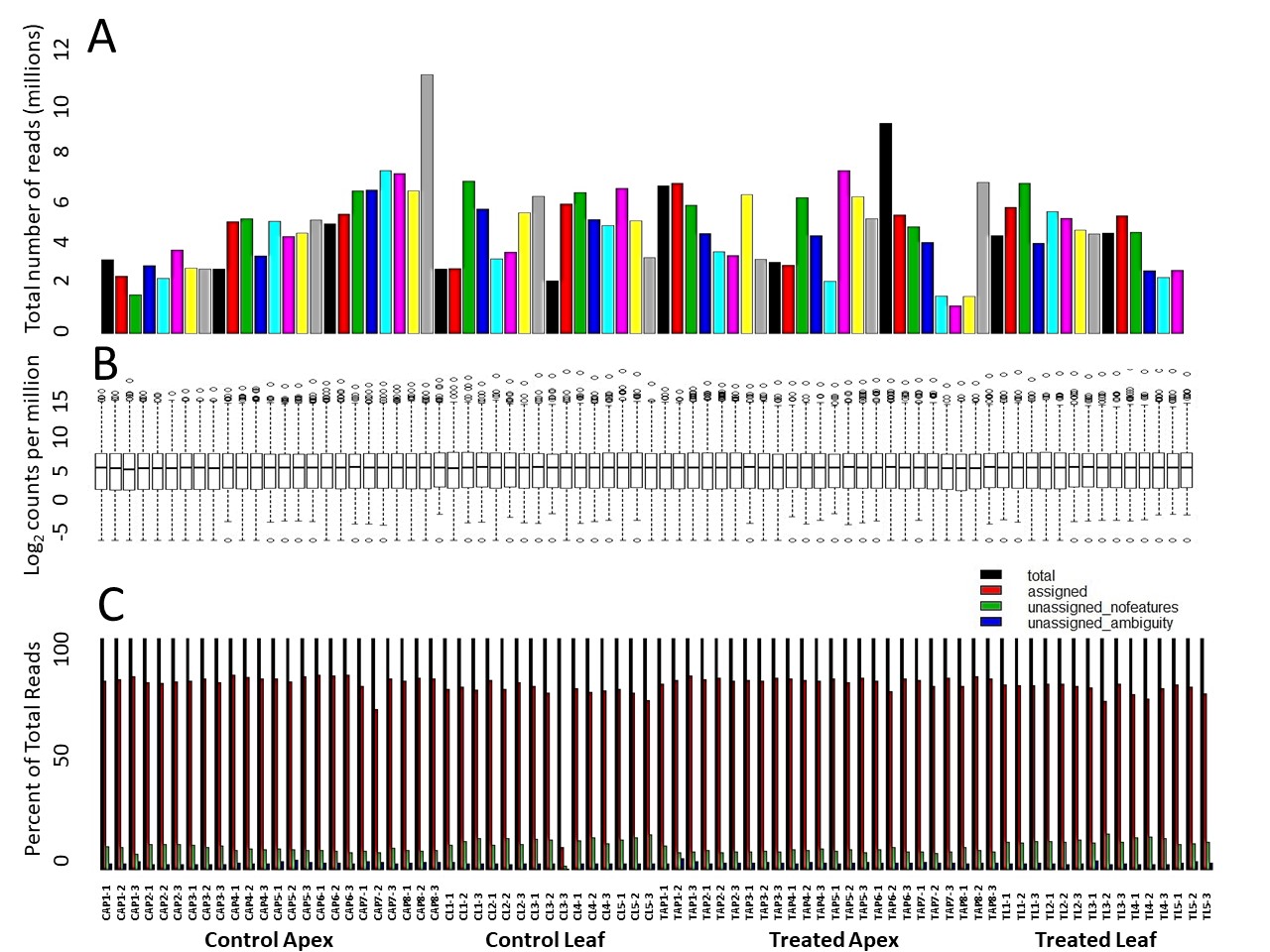
**

**Supplementary Figure SF3.** Feature map of the predicted DNA-binding sites. DNA motifs were grouped based on their Pfam domains. The x-axis corresponds to upstream length [-1500 bp, +200 bp] around the TSS. The y-axis corresponds to the density of captured sites with P-value <10 e−4. Black dots correspond the occurrence of each captured site.


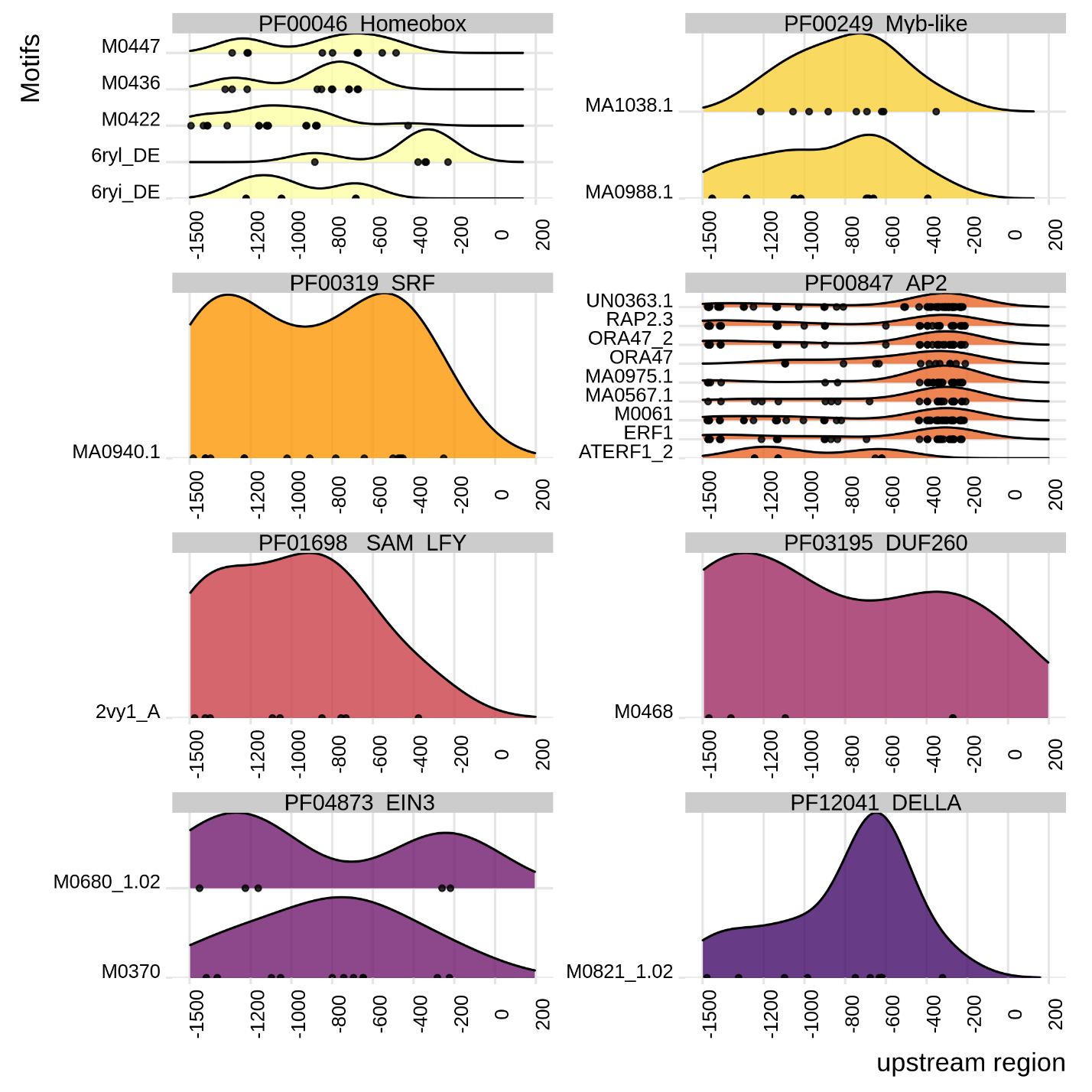


**Supplementary Figure SF4**. Expression patterns of target genes in broad categories with cis-domains to the transcription factors given in Figure 9 following ethephon treatment in the apex and leaf base.


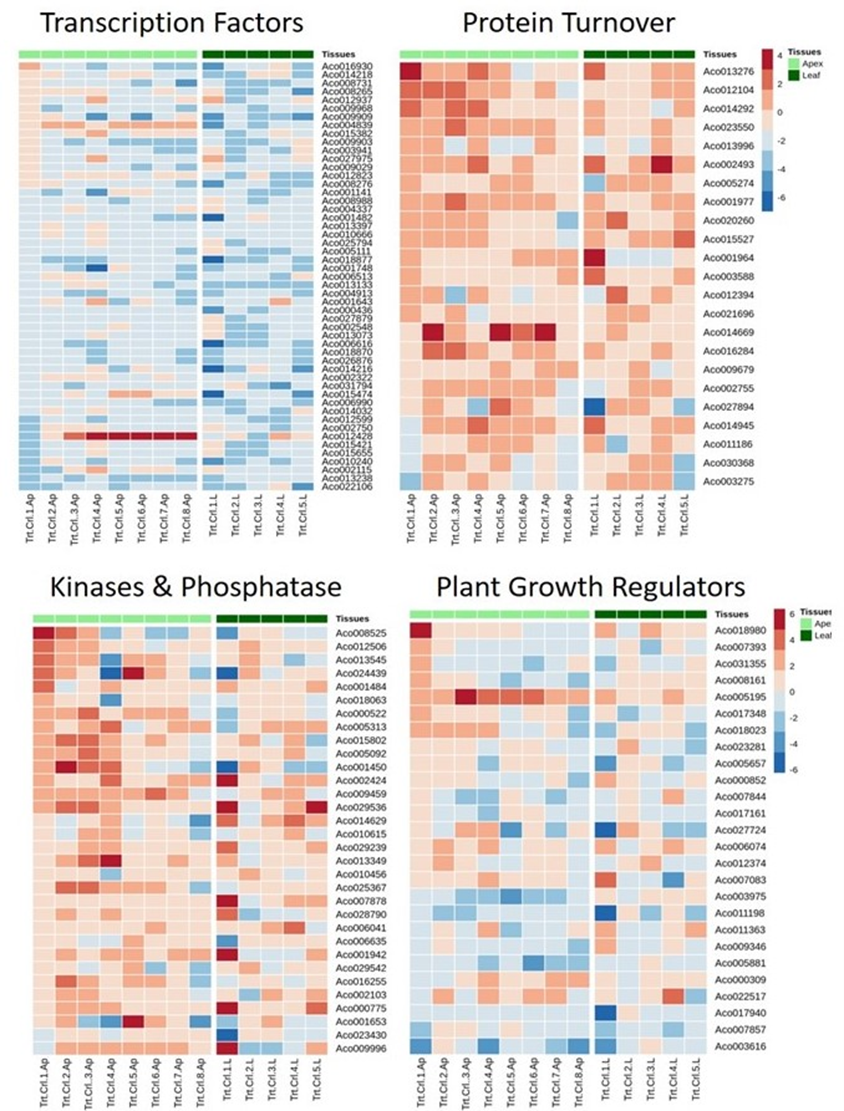


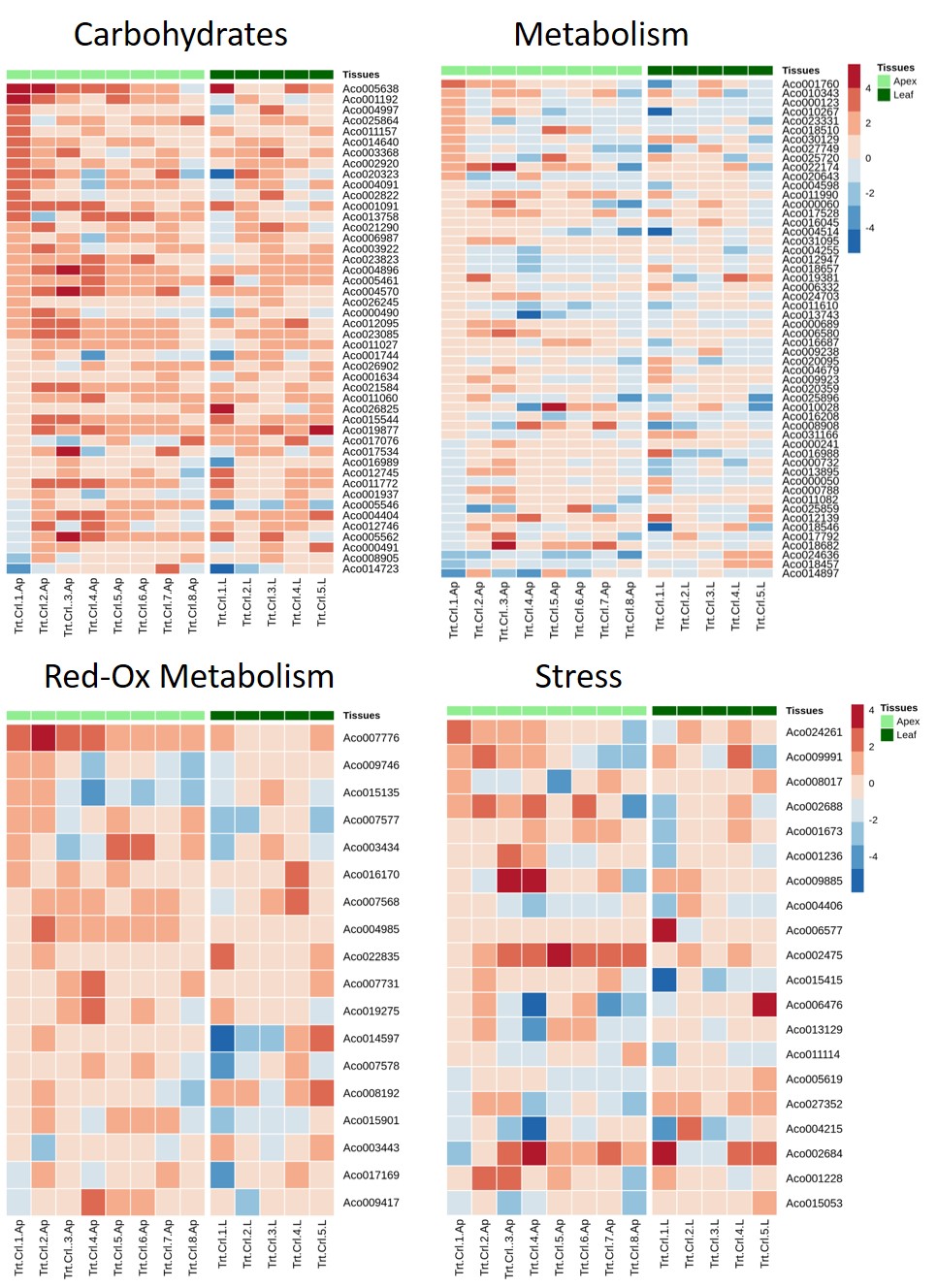


**Supplementary Figure SF5.** Gene expression network (GWENA) for treated apex at the same first sampling 6 hr after ethylene treatment. Ethylene responsive transcription factors were in all cluster except #4 and #14.


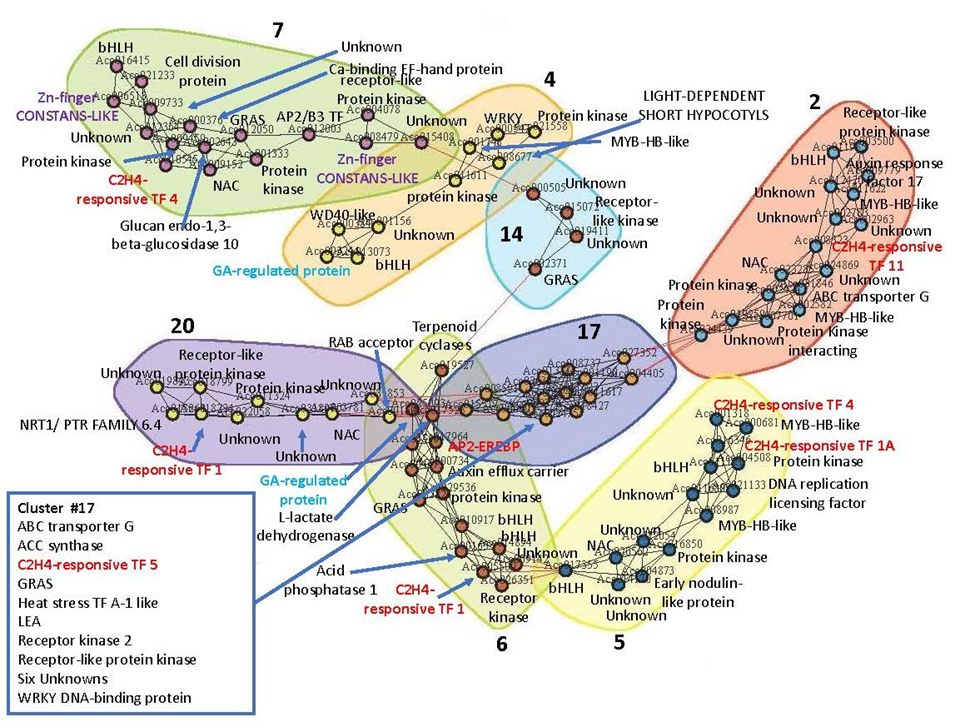

Supplement: Supplementary file 3 — Figure S1. InterPro domain motifs of the top twelve up‐ and down‐regulated differentially expressed genes. The AP2‐ERTF genes in the individual Pie Charts are boxed in a red rectangle. The number in each pie slice are the number of genes that were DE at that sampling. The complete list of InterPro domains and GO differentially expressed are found in Supplementary Table ST2. Figure S2. Pineapple flowering transcriptome library quality. A. Total number of reads, B. Normalized Log2 counts per million, and C. Assignment of total reads as percent of total from control and treated apex and leaf bases at different sampling times. Figure S3. Feature map of the predicted DNA‐binding sites. DNA motifs were grouped based on their Pfam domains. The x‐axis corresponds to upstream length [−1,500 bp, +200 bp] around the TSS. The y‐axis corresponds to the density of captured sites with P‐value <10 e−4. Black dots correspond the occurrence of each captured site. Figure S4. Expression patterns of target genes in broad categories with cis‐domains to the transcription factors given in Figure 9 following ethephon treatment in the apex and leaf base. Figure S5. Gene expression network (GWENA) for treated apex at the same first sampling 6 hr after ethylene treatment. Ethylene responsive transcription factors were in all cluster except #4 and #14. [file PLD3-7-e541-s003.docx]
